# Mechanism of hedysari radix praeparata cum melle and curcumae rhizoma herb pair in colitis-associated colorectal cancer through the MAPK/NF-κB signaling pathway: an investigation *in vivo* and *in vitro*

**DOI:** 10.3389/fchem.2025.1551722

**Published:** 2025-05-08

**Authors:** Ting Liu, Yugui Zhang, Feiyun Gao, Zhuanhong Zhang, Maomao Wang, Cui Ma, Yanjun Wang, Dingcai Ma, Zhe Wang, Xingke Yan, Yuefeng Li

**Affiliations:** ^1^ College of Pharmacy, Gansu University of Chinese Medicine, Lanzhou, China; ^2^ Scientific Research and Experimental Center, Gansu University of Chinese Medicine, Lanzhou, China; ^3^ College of Acupuncture-Moxibustion and Tuina, Gansu University of Chinese Medicine, Lanzhou, China

**Keywords:** hedysari radix praeparata cum melle-curcumae rhizoma (vinegar processed), colitis-associated colorectal cancer, drug-containing intestinal absorption solution, network analysis, intestinal permeability, MAPK/NF-kB signaling pathways

## Abstract

**Introduction:**

Astragali Radix (AR) - Curcumae Rhizoma (vinegar processed, CR) herb pair was recorded in ‘YIXUE ZHONGZHONG CANXILU’ to treat colitis-associated colorectal cancer (CAC). Hedysari Radix (HR) was categorized under the AR entry in ‘SHENNONG BENCAO JING’. HR is still an alternative to AR paired with CR clinically in northwest China. Hedysari Radix Praeparata Cum Melle (HRPCM) is a product that HR fries with honey to enhance the therapeutic effect. However, the mechanism of HRPCM paired with CR (HRCR) in CAC needs to be further elucidated.

**Methods:**

HRCR-MIAS were prepared using the eversion intestinal sac method. UHPLC Q-Exactive-MS investigated the compositions in HRCR-MIAS. Then, the mechanism of HRCR in CAC mice was predicted based on network pharmacology analysis in combination with the compositions in HRCR-MIAS. The pharmacodynamic effects of HRCR-MIAS for SW620 colon cancer cells were invested in vitro. The efficacies of HRCR low-, middle-, and high-dose groups (HRCR-L, 3.413 g/kg; HRCR-M, 6.825 g/kg; HRCR-H, 13.650 g/kg) in CAC mice were explored. Enzyme-linked immunosorbent assay (ELISA) kits were employed to assay The inflammatory factors levels, like IL-1β, IL-6, IL-10, and TNF-α in serum. The expressions of the intestinal permeability proteins, such as Claudin-1, Occludin, and ZO-1, were detected via immunohistochemical (IHC) analysis. Finally, the predicted signalling was verified by Western blot (WB).

**Results:**

855 common components were identified in HRCR and HRCR-MIAS, and 25 specific components in HRCR-MIAS were pointed out. Based on network pharmacology analysis, the inflammatory response and the cross-linked MAPK signalling and NF-kB signalling were predicted to be the main reasons for HRCR in CAC. HRCR-MIAS inhibited the proliferation, induced apoptosis, regulated the cell cycle progression, and restrained the SW620 cells’ ability to migrate and invade in vitro. The outcomes of the WB experiment exhibited that HRCR-MIAS inhibited the expression of key proteins such as MEKK1, RAS, ERK, IKB and NF-kB in the MAPK/NF-kB signalling pathway of SW620 cells. The study in vivo found that the different doses of HRCR recovered the loss of body weight, the shortened colon length, the increased tumour counts, the abnormal changes in spleen and thymus indices, the colonic lesions, the unbalanced inflammatory factors levels like IL-10, IL-6, IL-1β, and TNF-α in serum, and the down-regulated intestinal permeability proteins such as Claudin-1, Occludin, and ZO-1. Experimental validation by WB confirmed that HRCR inhibited the expression of the key proteins, including MEKK1 RAS, ERK, IKB, and NF-kB, in the MAPK/NF-kB signalling in CAC mice.

**Discussion:**

HRCR not only suppresses the process of colonic inflammation and improves intestinal permeability but also relieves CAC by inhibiting the activated MAPK/NF-kB signalling cascade to alleviate CAC.

## Highlights

• The active ingredients in HRCR-MIAS have been identified.
• The inhibitory effect of HRCR-MIAS on SW620 colon cancer cells was elucidated *in vitro*.
• The mechanism of HRCR in treating CAC mice by inhibiting the MAPK/NF-kB signaling cascade was demonstrated both *in vitro* and *in vivo*.

## 1 Introduction

Colorectal cancer (CRC) is recognized as the third leading cause of cancer-related mortality. Early screening and diagnosis are essential for effectively managing colorectal cancer (Fabregas et al., 2022). Inflammatory bowel disease (IBD), which includes ulcerative colitis (UC) and Crohn’s disease (CD), is a significant risk factor for the development of CRC. The long-term, persistent inflammation associated with IBD heightens the risk of colorectal carcinogenesis (Shawki et al., 2018). The progression to colitis-associated colorectal cancer (CAC) follows the ‘inflammation-dysplasia-carcinoma’ sequence (Majumder et al., 2022), which involves mutations in oncogenes such as TP53 and K-RAS (Alpert et al., 2019) and is linked to pathological increases in mucosal permeability (Lavoie et al., 2020; Xu et al., 2021). Various molecular pathways and mechanisms regulate CAC carcinogenesis, including the NF-kB pathway, the Wnt pathway, and the STAT3 and IL-6/p-STAT3 pathways (Hirano et al., 2020; Majumder et al., 2022). Furthermore, the transformation process of CAC is associated with immune and inflammatory responses, DNA damage and repair, cell proliferation, invasion and migration, and cellular metabolism (Xue et al., 2018).

Early intervention in colonic inflammation is one of the most effective methods for preventing colorectal cancer (CAC). Traditional Chinese Medicine (TCM) has demonstrated significant potential in CAC prevention due to its multifaceted characteristics, including various components, multiple targets, and the ability to act on diverse biological pathways (Duan et al., 2023). A recent review has adequately elucidated the advantages of TCM in the context of CAC (Wei et al., 2023). It was found that the impact of TCM on CAC is primarily manifested in detrimental biological processes, including inflammation-mediated oxidative stress, cell apoptosis, and cell proliferation, as well as the improvement of the tumor microenvironment and the imbalance of intestinal microecology (Wei et al., 2023). These harmful processes are mainly associated with several signaling pathways, including NF-κB, STAT3, Wnt/β-catenin, HIF-1α, and Nrf2 (Wei et al., 2023). Astragali Radix (AR) is derived from the dehydrated root of either Astragalus membranaceus (Fisch.) Bge. var. mongholicus (Bge.) Hsiao, or A. membranaceus (Fisch.) Bge (Fu et al., 2014). Hedysari Radix (HR) stems from the dried root of Hedysarum polybotrys Hand.-Mazz (Mo et al., 2022). AR and HR belong to the Leguminosae family but are classified under different genera (Zhang et al., 2022). According to the ‘SHENNONG BENCAO JING’ record, HR was initially categorized under the AR content. However, Hedysarum polybotrys was first listed separately as HR in the 1985 edition of the Chinese Pharmacopoeia. In clinical practice, both AR and HR can be used interchangeably (Tan et al., 2019). Notably, HR and AR share similar pharmaceutical properties, including anti-inflammatory, anti-cancer, immunoregulatory, anti-gastric ulcer, hypoglycemic, and hepatoprotective effects (Fu et al., 2014; Mo et al., 2022; Tang and Huang, 2022). Curcumae Rhizoma (CR) is derived from the dehydrated rhizome of Curcuma kwangsiensis S. G. Lee et C. F. Liang, Curcuma phaeocaulis Valeton, and Curcuma wenyujin Y. H. Chen et C. Ling (Zhao P. et al., 2023). Previous studies have demonstrated that CR has a significant therapeutic impact on various cancers, including colon (Teng et al., 2022), gastric (Zu et al., 2023), liver (Gao et al., 2022), and ovarian cancers (Gao et al., 2022). CR can notably delay cell cycle progression, induce apoptosis, and inhibit tumor metastasis and invasion (Lu et al., 2012).

AR-CR herb pair is primarily utilized in clinical settings to manage gastrointestinal diseases, such as gastrointestinal inflammation and cancer (Ji et al., 2021; Sun et al., 2021; Guo et al., 2022; Tan et al., 2023). In China, the AR-CR herb pair was first documented in ‘YIXUE ZHONGZHONG CANXILU’ by Xichun Zhang. Notably, in northwest China, particularly in Longnan, Gansu Province, AR is often replaced with HR for clinical use, as HR is considered a genuine medicinal material in Longnan City (Zhang et al., 2022). Previous studies have confirmed that HR is more effective than AR in regulating gastrointestinal inflammation and immunity (Yang et al., 2018; Zhang et al., 2022). In clinical practice, HR and AR are often processed with honey to improve anti-inflammatory and immune-regulating properties, while immune regulation (Wu et al., 2021; Zhang et al., 2022), CR is typically treated with vinegar to boost its anti-cancer activity (Gu et al., 2016; Zhang et al., 2017). These processed products are referred to as Astragali Radix Praeparata Cum Melle (ARPCM), Hedysari Radix Praeparata Cum Melle (HRPCM), and Vinegar-Processed Curcumae Rhizomaon (VPCR), respectively, in TCM. Consequently, the efficacy of HRPCM as an alternative to ARPCM paired with VPCR in CAC therapy was investigated preferentially. Our previous study optimized the pharmacodynamic effects of different compatibility ratios of HR and VPCR on CAC based on our clinical experience. We found that the ratio of 4:1 between HR and VPCR was optimal for anti-CAC efficacy. We further investigated the effects of HR-VPCR and HRPCM-VPCR (HRCR) in treating CAC on the same basis and found that HRCR was better than HR-VPCR in preventing and controlling CAC. Therefore, we further investigated the effects of different administration doses of HRCR on CAC mice on this basis and studied the mechanism of action of HRCR in treating CAC from the perspective of *in vivo* and *in vitro*.

Network analysis is a multidisciplinary analytical method based on systems biology theory, which comprehensively evaluates the relationships among drugs, active ingredients, targets, pathways, and diseases through biological network models (Jiashuo et al., 2022; Yuan et al., 2022). Medicated intestinal absorption solution (MIAS)makes up for the shortcomings of the complex composition of TCM compounds, which cannot be directly subjected to pharmacological experiments *in vitro*. *In vitro*, MIAS is increasingly being utilized in scientific research as a novel technique for assessing the pharmacological effects of TCM (Zhang et al., 2018). The eversion intestinal sac method is the main production method for MIAS. This method allows for the rapid determination of vegetable drug’s efficacy and material basis in disease management (Guo et al., 2018). Consequently, the components of MIAS, when combined with network analysis, facilitate the prediction and exploration of the potential mechanisms and material foundations of TCM in managing diseases. In the present study, we first prepared the MIAS of HRCR using the eversion intestinal sac method. We then assessed the inhibitory effect of the MIAS of HRCR on colon cancer SW620 cells *in vitro*. Finally, the components of MIAS from HRCR, in conjunction with network analysis, were employed to predict the potential mechanisms, and experimental verification *in vivo* was conducted to confirm the mechanisms of HRCR in CAC.

## 2 Materials and methods

### 2.1 Preparation of HRCR extracts

*Hedysarum polybotrys* (Batch No. 20211021) was received in Micang Mountain District, Longnan City, Gansu Province, China. Vinegar-processed Curcumae Rhizoma (*Curcuma wenyujin*. Batch No. 2301012) was obtained from Longxi County Zhengxin Pharmaceutical Co. LTD, China. These medicinal materials were identified as genuine by Professor Mingwei Wang from the Authentication Department of Chinese Medicines, College of Pharmacy, Gansu University of Chinese Medicine. The HRPCM was created based on our prior investigation (Zhang et al., 2022). The specific preparation method of HRPCM can be found in Supplementary Method S1. The preparation of HRCR extracts was performed based on our pre-experiments. The extract was collected by refluxing Hedysari Radix Praeparata Cum Melle (36 g) and Vinegar-processed Curcumae Rhizoma (9 g) with 6-fold volume of pure water for 1 h. Volatile oils were also gathered. This stage was performed two times. Then, the extracts were combined, filtered, and concentrated. Lastly, all concentrates were lyophilized, sealed, and kept at 4°C in a refrigerator.

### 2.2 Preparation and qualitative analysis of medicated intestinal absorption solution for HRCR

#### 2.2.1 Preparation of medicated intestinal absorption solution for HRCR

A medicated intestinal absorption solution for HRCR was prepared according to the methods described in the literature (Li et al., 2020). The lyophilized powder of HRCR was redissolved with a tyrode’s solution (Nos. WH2923Z031, Procell Life Science& Technology Co., Ltd.) and then prepared as a tyrode’s compound solution containing crude drugs at 0.945 g/mL for spare. SPF-grade SD rats were fasted for 12 h prior to the investigation and allowed to drink water unrestrictly. 2% pentobarbital sodium (Nos. 211004, Fujian Mindong Lijiexun Pharmaceutical Co. Ltd.) was administered intraperitoneally to anesthesia the rats. After sampling, the mesentery was stripped, the contents of the intestinal canal were cleared, and four segments of intestinal segments were taken from the pylorus to the ileocecal valve, each measuring 14 cm long. The four intestinal segments taken were rinsed with the tyrode solution, then flipped over and ligated to form a capsule. The tyrode compound solution of HRCR was reconstituted by adding 90 mL into Mai’s bath tubes, and 95% O2 and 5% CO2 gases were passed through the tube, which was equilibrated at 37°C for 5 min. The intestinal tubes were injected with 2 mL of the tyrode solution and then placed in Mai’s bath tube for 2 h later. Then, the contents obtained from 4 sections of intestinal tubes were collected and filtered through 0.22 μm cellular filters to remove bacteria. In this way, the medicated intestinal absorption solution of HRCR was obtained and stored at 4°C for spare use.

#### 2.2.2 Composition analysis of medicated intestinal absorption solution for HRCR

800 μL of methanol was added into 100 μL of medicated intestinal absorption solution for HRCR, vortexed, and mixed for 60 s. The sample was allowed to stand at −20°C for half an hour and underwent 20-min centrifuging at 16000 g at 4°C. The supernatant was taken and dried by vacuum. The residue was added 100 μL of 40% aqueous methanol solution, vortexed, and underwent 15 min centrifuging at 4°C at 16000 g, and the supernatant was taken and obtained. Qualitative analysis of aqueous extract solution for HRCR (**HRCR**), control intestinal absorption solution (**Con-IAS**), and medicated intestinal absorption solution of HRCR (**HRCR-MIAS**) were implemented by UHPLC Q-Exactive-MS. The chromatographic condition, Mass Spectrometry Conditions, and the method of operation were provided in Supplementary Method S2.

### 2.3 Target prediction based on network analysis combined with the results of UHPLC Q-Exactive-MS analysis

The detected components of HRCR-MIAS employing UHPLC-Q-Exactive MS were introduced into PubChem (https://pubchem.ncbi.nlm.nih.gov/) and retrieved to acquire the Canonical SMILES constitutional formula. From this, the targets of the above active components were predicted on the SwissTarget website (http://swisst.argetprediction.ch/). The targets were required to have the parame-ters ‘*Mus musculus*’ and ‘Probability>0’, and the duplicate targets were removed to get the ultimate targets. Meanwhile, the targets of ‘colitis-associated colorectal cancer,’ ‘colorectal cancer,’ and ‘colitis’ were collected from Gene Cards (http://www.genecards.org), OMIM Database (https://omim.org/), Database of Therapeutic Target (https://db.idrblab.net/ttd/), respectively. The acquired targets were intersected, and the duplicates were deleted. After that, component and disease targets were intersected to obtain the “common targets” in venny 2.1.0 (https://bioinfogp.cnb.csic.es/tools/venny/index.html).

The common targets were submitted to the database of STRING 11.0 (https://cn.string-db.org/) to construct the PPI network diagram according to the ‘*Homo sapiens*’ of the biological species. Then, the “TSV.” format file was downloaded and plotted in Cytoscape software. Moreover, GO and KEGG enrichment analyses were conducted utilizing the common targets. Finally, a Components-targets network diagram was generated employing the Cytoscape program.

### 2.4 Study on the efficacy of HRCR-MIAS on SW620 cells

#### 2.4.1 Cell culture

SW620 cells line, provided by Cell bank, Chinese Academy of Sciences, underwent culturing with L-15 (HyClone. No. AJ30725631) medium treated with 10% and 1% of fetal bovine serum and penicillin-streptomycin liquid (100X, No. 20221012, Solarbio), respectively. An incubator with 37°C and 5% CO_2_ was utilized to facilitate the growth of the culture medium in a no-vent T25 culture flask.

#### 2.4.2 Proliferation assays and screening of the optimal intervention concentration

In this study, 5-fluorouracil (5-Fu, No. FA211108, Shanghai Xudong Haipu Pharmaceutical Co., Ltd.) was adopted as a positive control drug due to due to the significant anti-colorectal cancer activity (Andre et al., 2004; Ghosh et al., 2022). SW620 cells in logarithmic growth stages were taken, trypsin-digested, and measured, and the cell density was set to 6 × 103 cells/well. Each well was inoculated with 100 μL of PBS solution in a 96-well plate, and the outermost wells were all added with PBS solution and underwent incubation in a 5% CO_2_ incubator. Following the attachment of cells to the wall for 24 h, the supernatant was eliminated, and 100 μL of all the medium containing the medication was supplemented. Then 25, 50, 100, 200, 400, 800, 1600, 3200, and 6400 μM concentrations of 5-FU and 4%, 6%, 8%, 10%, 12%, and 14% concentrations of HRCR-MIAS were added respectively to intervene the SW620 cells, and each group was set up with 3 compound wells. The culture system was terminated after 24, 48, and 72 h of intervention. Typically, 10 μL CCK-8 was introduced to each culture well. (No. C6005, New Cell & Molecular Biotech Co., Ltd.). solution and cultured at 37°C for 2 h. An automatic enzyme labeling instrument was employed to detect the optical density value of every well, and the cell growth rate was calculated. Cell growth rate (%) = (OD value of drug intervention group - OD value of blank well no cell group/ OD value of cell culture no intervention group - OD value of blank well no cell group) × 100%. The half maximal inhibitory concentration (IC50) of HRCR-MIAS and 5-Fu on SW620 cells at 24, 48, and 72 h were detected utilizing GraphPad Prism 8 (GraphPad program, La Jolla, CA), respectively, to determine the optimal intervention concentration. Whereafter, the proliferative effect of 5-Fu and HRCR-MIAS were assayed based on the optimal concentration on SW620 cells, respectively. In this step, 100 μL of complete medium was employed to incubate the control group (Control). 5-Fu administration group was used as a positive control group. The groups of HRCR-MIAS with high-, medium- and low-dosage (HRCR-ML, HRCR-MM, and HRCR-MH) were also set based on the optimal concentration.

#### 2.4.3 Detection of cell apoptosis and cell cycle via flow cytometry

The cultured cells underwent resuspension with 500 μL apoptosis kit binding solution added 5 L Annexin V-FITC, stained at 4°C for 15 min avoiding light, added 10 μL PI to stain for 15 min, and detected cell apoptosis. Meanwhile, the cultured cells underwent half an hour of incubation at room temperature with 500 and 5 μL of DNA Staining and Permeabilization solutions without light to detect the cell cycle. Flow cytometry was employed to identify the apoptosis and cell cycle.

#### 2.4.4 Detection of Cell migration by cell scratch test

When the cells were cultured to a density of 80%–90%, a horizontal line was drawn on the inferior part of the 6-well plate. The tip of the 200 μL pipette was employed to perform a scratch perpendicular to the horizontal line in the wells and washed twice with sterile PBS to remove dead cells. The culture system was continued by re-adding the drug-containing serum-free medium. The changes in the scratched area were observed at 0 h and 24 h utilizing an inverted microscope, respectively. The scratch distance was measured with ImageJ 1.53t software (Java 1.8.0_322, 64-bit). Three sites were randomly taken in each well to determine the growth motility of the cells. Cells scratch healing rate (%) = [(scratch area in 0 h - scratch area in 48 h)/ scratch area in 0 h] × 100%.

#### 2.4.5 Detection of cell invasion by transwell assay

Matrigel matrix gel was diluted at 4°C with a pre-cooled medium free of serum at a 1:8 ratio. 50 μL of the above diluent was spread evenly in the superior Transwell chamber without air bubbles and solidified overnight in an incubator at 37°C. The SW620 cells were inoculated at 1 × 105 cells/well in the superior Transwell chamber with an 8 μm pore. The chamber was inserted into the plate wells during the experiments, and 800 μL of a medium, including 10% serum, was introduced to the inferior chamber. Following 48 h, the cells were treated for 15 min with 4% paraformaldehyde and then subjected for 15 min to 0.1% crystal violet stain and then inverted. An inverted microscope was employed to photograph and observe the SW620 cells. The cells were counted and evaluated for invasive ability using ImageJ 1.53t software. Invasion inhibition rate (%) = [(number of cells in the control group - number of cells in the experimental group)/number of cells in the control group] × 100%.

#### 2.4.6 Western blot

SW620 cells were lysed using RIPA lysis (R0010, Beijing Solarbio Science & Technology Co., Ltd., China) to extract the total proteins. A BCA Concentration Assay Kit (R0020, Beijing Solarbio Science & Technology Co., Ltd.) was utilized to determine the content of the extracted proteins. Proteins were divided by SDS-PAGE. PVDF membrane was used to transfer the proteins. The primary antibodies including RAS antibody (21 kDa, 1:5000, ab52939, No. 1014133-14, Abcam, UK), ERK antibody (42, 44 kDa, 1:1000, ab201015, No. 1001375-16, ab184699, No. 1000861-11, Abcam, UK), p-ERK antibody (42, 44 kDa, 1:1000, No. #4695, No. 4370T, Cell Signaling Technology, America), MEKK1 antibody (195 kDa, 1:200, No. I1710, Santa Cruz Biotechnology, Inc., America), IkB antibody (36 kDa, 1:10000, ab32518, No. GR275907-51, Abcam, UK), NF-kB antibody (65 kDa, 1:10000, ab32536, No. GR3422076-12, Abcam, UK), GAPDH antibody (37 kDa, 1:10000, No. YM3092, Immunoway, America), and β-actin antibody (43 kDa, 1:3000, No. #AF7018, Affinity, China) were incubated followed by incubation of the secondary antibody, washed, and exposed with the gel imaging system. ImageJ software was adopted to analyze the expressions of these target proteins.

### 2.5 Study on the efficacy of HRCR on CAC mice

#### 2.5.1 Animal model and treatment

The male C57/6J mice were provided and authorized by the animal experimental center of Gansu University of Chinese Medicine (animal permit No. SCXK (Gan) 2015-0002). 60 C57/6J mice were raised in an animal laboratory of specific pathogen-free (SPF) standards at Gansu University of Chinese Medicine. Azoxymethane and Dextran Sulfate Sodium (AOM/DSS) induced CAC model mice. After 1 week of adaptive feeding, 60 C57/6J mice were distributed into six groups at random: Control group (Control, n = 10), AOM/DSS model group (AOM/DSS, n = 10), AOM/DSS + SASP (SASP, positive control group, 0.455 g/kg, n = 10). SASP is used to treat IBD based on the current guidelines (Sasson et al., 2021), AOM/DSS + HRCR low dose group (HRCR-L, 3.413 g/kg, n = 10), AOM/DSS + HRCR middle dose group (HRCR-M, 6.825 g/kg, n = 10), and AOM/DSS + HRCR high dose group (HRCR-H, 13.650 g/kg, n = 10). The animal intervention method was mentioned in our prior investigation (Zhang et al., 2023). The specific intervention procedures for HRCR in CAC mice were described in Supplementary Method S3. The mice were weighed every 7 days, and the status of diarrhea and fecal bleeding was documented throughout the investigation. The Disease Activity Index (DAI) score was reckoned during the experiment according to the rules in Supplementary Table S1. Colon tissue, serum, spleen, and thymus were obtained after the investigation.

#### 2.5.2 Enzyme-linked immunosorbent assay

The Enzyme-linked immunosorbent assay (ELISA) kits were utilized to assay the TNF-α, IgA, IL-6, IL-10, IL-1β, and COX-2 (No. 20230629-20852A, 20230629-20174A, 20230629-20188A, 20230629-20162A, 20230629-20506A, and 20230629-20978A. Shanghai Enzyme-linked Biotechnology Co., Ltd.) expressions in serum. The determination method was carried out based on the manufacturer’s directions.

#### 2.5.3 H&E pathological observation

Colonic tissues soaked with 4% paraformaldehyde underwent embedding and sectioning, then were subjected to hematoxylin-eosin (H&E) stains, dehydrated, and sliced to prepare H&E pathology sections. H&E pathological scores were evaluated according to the rules in Supplementary Table S2.

#### 2.5.4 Immunohistochemical (IHC) analysis

Embedded colon tissues were sectioned and blocked with primary antibody (claudin-1 antibody, No. 64f7244, Affinity, China; occludin antibody, No. GR3243495-28, Abcam, America; ZO-1 antibody, No. 44h7470, Affinity, China), and then incubated with secondary antibody. After DAB color development, the nuclei were counterstained with hematoxylin and examined microscopically. ImageJ software was utilized to analyze the claudin-1, occludin, and zonula occludens-1 (ZO-1) expressions.

#### 2.5.5 Western blot

Colon tissue was lysed using RIPA lysis to extract the total proteins. A BCA Concentration Assay Kit was employed to determine the content of the extracted proteins. SDS-PAGE was utilized to divide the proteins. PVDF membrane was used to transfer the proteins. Western blot experiments were carried out using the method in item 2.4.6, and the experimental data were examined using the ImageJ program.

### 2.6 Statistical analysis

This investigation exhibited all data as mean ± standard deviation (SD). *p* < 0.05 represents statistically significant. Paragraph Prism 8.0.2 (Paragraph Prism Program, La Jolla, CA) was employed to process the data.

## 3 Results

### 3.1 Composition analysis of HRCR-MIAS

**FIGURE 1:**
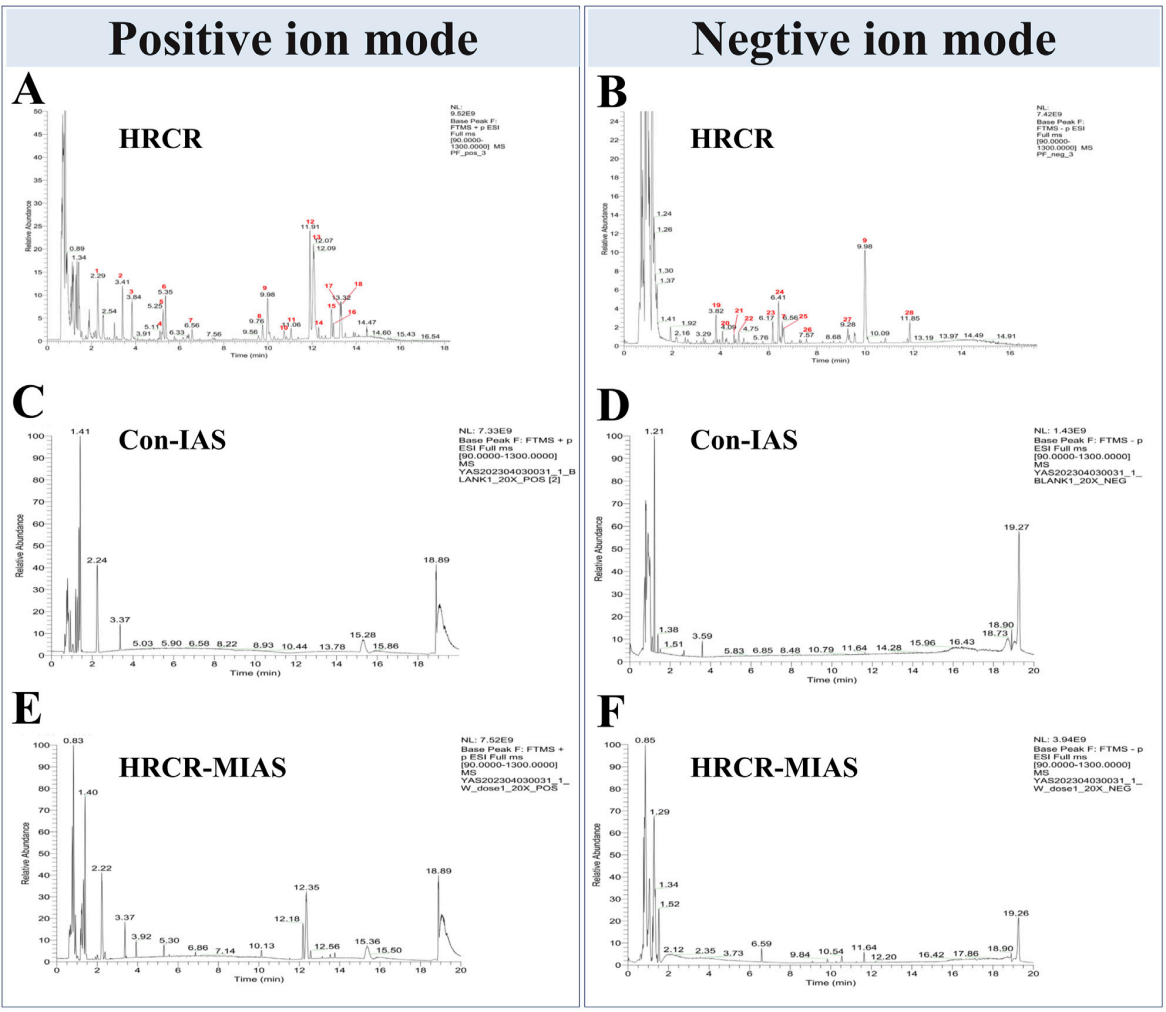
BPC diagrams of HRCR, Con-IAS, and HRCR-MIAS. **(A)** Positive ion mode diagram of HRCR. **(B)** Negative ion mode diagram of HRCR. **(C)** Positive ion mode diagram of Con-IAS. **(D)** Negative ion mode diagram of Con-IAS. **(E)** Positive ion mode diagram of HRCR-MIAS. **(F)** Negative ion mode diagram of HRCR-MIAS. HRCR, aqueous extract solution for HRCR. Con-IAS, control intestinal absorption solution. HRCR-MIAS is a medicated intestinal absorption solution for HRCR.

**FIGURE 2:**
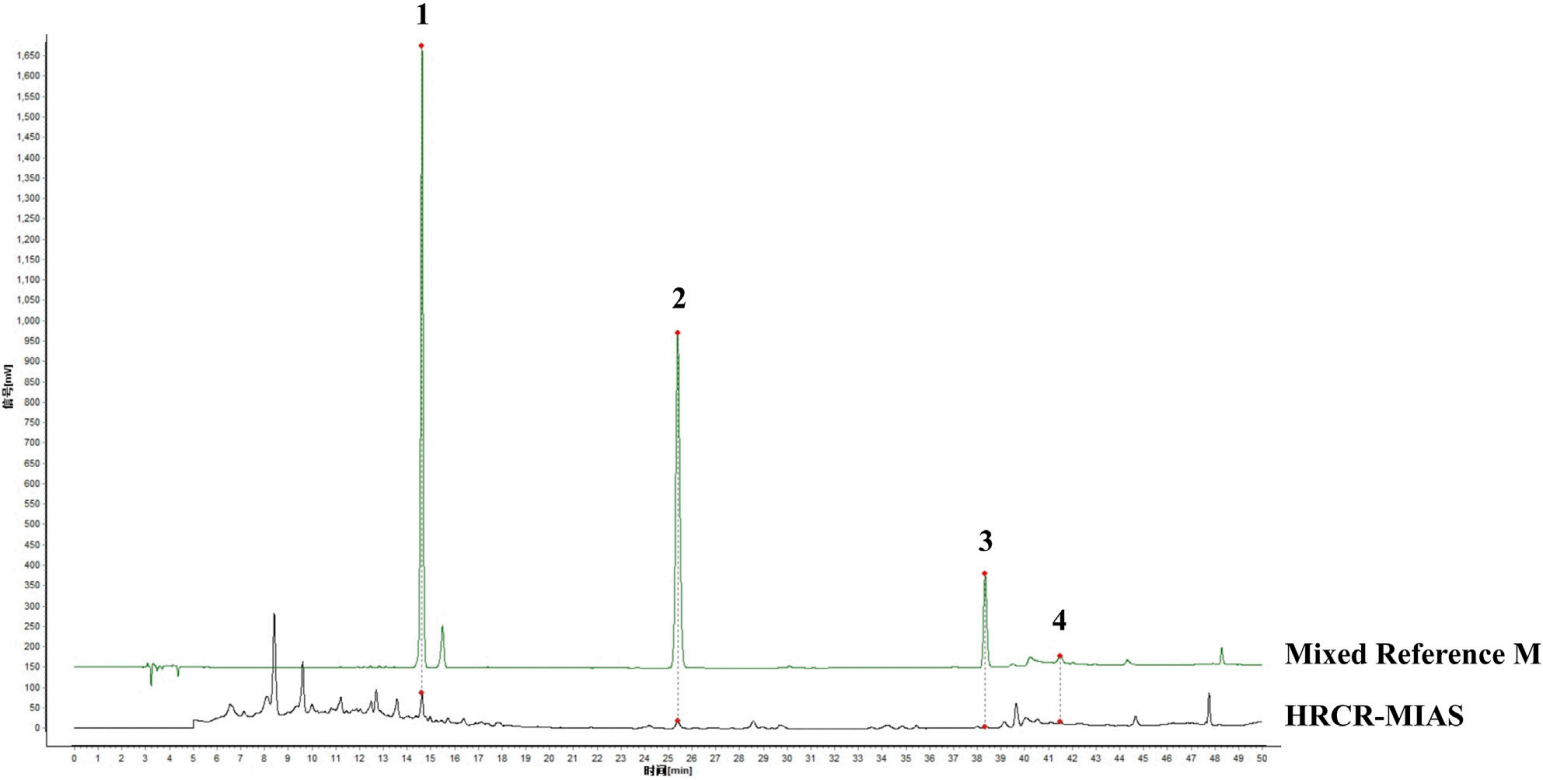
HPLC plot of HRCR-MIAS and mixed controls. 1-Ononin; 2-Formononetin; 3-Curdione; 4-Curcumenol.

The qualitative analysis of HRCR-MIAS was carried out by Shanghai Applied Protein Technology Co., Ltd. Figure 1 exhibited the Base Peak Chromatogram (BPC) of HRCR, Con-IAS, and HRCR-MIAS after UHPLC Q-Exactive-MS analysis. 855 common components were identified in HRCR and HRCR-MIAS (Supplementary Table S3). The qualitative and quantitative results among HRCR, Con-IAS, and HRCR-MIAS were compared, and 28 peaks were identified and accurately labeled. 27 peaks (Peak 2- peak 28) were identified as the compounds in HRCR-MIAS. This result was obtained according to the requirement that the peak response intensity in HRCR was more than three times higher than that in HRCR-MIAS (Zu et al., 2023). However, no specific names were identified for Peak 5 and Peak 24. Hence, we identified 25 specific components in HRCR-MIAS, detailed in Table 1. Using Ultra-Performance Liquid Chromatography (UPLC), we quantified the concentrations of Ononin, Formononetin, Curdione, and Curcumenol in HRCR-MIAS as follows: 0.4643 ± 0.009 μg/mL, 0.1508 ± 0.012 μg/mL, and 0.5106 ± 0.003 μg/mL, respectively. Figure 2 presents the chromatograms of HRCR-MIAS alongside the mixed control chromatograms.

**TABLE 1:** Chemical composition identification of BPC icon peak.

No.	m/z	RT (min)	ppm	Adduct	Score	Compound name	Class	Into dose or None
1	166.0863	2.30	17.7	[M + H]+	0.9997	Phenylalanine	Carboxylic_acids_ and_derivatives	None
2	188.0707	3.42	0.4	[M + H-H_2_O]+	0.9741	3-Indolyllactic acid	Indoles_and_ derivatives	Into_Dose
3	247.1439	3.86	1.3	[M + H]+	0.9707	Lenticine	Carboxylic_acids_ and_derivatives	Into_Dose
4	235.1690	5.12	1.1	[M + H]+	0.8316	Artemisinate	Prenol_lipids	Into_Dose
5	685.8634	5.25	NA	NA	NA	Unidentified name	NA	Into_Dose
6	267.1586	5.37	1.1	[M + H]+	0.9457	5,8-Dihydroxy-1,5,8-trimethyl-4,5a,6,7,8a,9-hexahydro-3aH-azuleno [6,5-b]furan-2-one	Prenol_lipids	Into_Dose
7	431.1334	6.57	1.1	[M + H]+	0.9998	Ononin	Isoflavonoids	Into_Dose
8	235.1692	9.76	0.5	[M + H]+	0.8883	Isopetasol	Prenol_lipids	Into_Dose
9	269.0805	10.00	0.3	[M + H]+	0.9974	Formononetin	Isoflavonoids	Into_Dose
10	249.1484	10.74	0.8	[M + H]+	0.9431	Parthenolide	Prenol_lipids	Into_Dose
11	271.0965	11.06	0.4	[M + H]+	0.9968	(−)-Medicarpin	Isoflavonoids	Into_Dose
12	235.1696	11.91	0.9	[M + H-H_2_O]+	0.8167	Ageratriol	Prenol_lipids	Into_Dose
13	235.1692	12.08	0.1	[M + H-C9H10O3]+	0.8637	10-Hydroxy-1,6-dimethyl-9-(propan-2-yl)-5,12-dioxatricyclo [9.1.0.04,6]dodecan-8-yl (2E)-3-phenylprop-2-enoate	Prenol_lipids	Into_Dose
14	235.1691	12.07	0.2	[M + H]+	0.9810	Curcumenol	Prenol_lipids	Into_Dose
15	237.1847	12.88	0.7	[M + H]+	0.9844	Curdione	Prenol_lipids	Into_Dose
16	229.1225	12.97	0.6	[M + H-C4H10O5]+	0.9913	Innuchenenolide C	Prenol_lipids	Into_Dose
17	215.1431	13.28	0.3	[M + H-2H_2_O]+	0.9257	Nardosinon	Prenol_lipids	Into_Dose
18	217.1587	13.33	0.8	[M + H-C5H10O4]+	0.9466	2,3-Dimethyloxirane-2-carboxylic acid [(1S,2R,4aR,8aR)-1-hydroxy-7-isopropylidene-6-keto-1,4a-dimethyldecalin-2-yl] ester	Prenol_lipids	Into_Dose
19	175.0604	3.82	4.7	[M-H]-	0.9975	alpha-Isopropylmalate	Fatty_Acyls	Into_Dose
20	281.1393	4.09	0.8	[M-H]-	0.834	1H-3,9a-Methanocyclopent [c]oxocin-4-carboxylic acid, octahydro-4-hydroxy-7-(2-hydroxy-1-methylethylidene)-	Hydroxy_acids_and_derivatives	Into_Dose
21	281.0665	4.56	6.1	[M-H]-	0.9468	Feruloyl Lactate	NA	Into_Dose
22	121.0283	4.76	8.3	[M-H]-	0.9987	4-Formylphenol	Organooxygen_compounds	Into_Dose
23	187.0968	6.17	4.2	[M-H]-	0.9875	Azelaic acid	Fatty_Acyls	Into_Dose
24	391.1762	6.42	NA	NA	NA	Unidentified name	NA	Into_Dose
25	137.0234	6.63	7.5	[M-H]-	0.9987	4-Hydroxybenzoic acid	Benzene_and_substituted_derivatives	Into_Dose
26	283.0611	7.57	0.9	[M-H]-	0.9959	Calycosin	Isoflavonoids	Into_Dose
27	329.2332	9.28	0.4	[M-H]-	0.9862	9-Octadecenoic acid, 5,8,11-trihydroxy-	Fatty_Acyls	Into_Dose
28	311.2227	11.85	0.5	[M-H]-	0.9853	(9Z,12E)-15,16-Dihydroxyoctadeca-9,12-dienoic acid	Fatty_Acyls	Into_Dose

Note: “Into Dose or None” represents the compound that enters or disappears into the intestine.

### 3.2 Signaling prediction depended on the pharmacology of the network in combination with the results of UHPLC Q-Exactive-MS analysis

**FIGURE 3:**
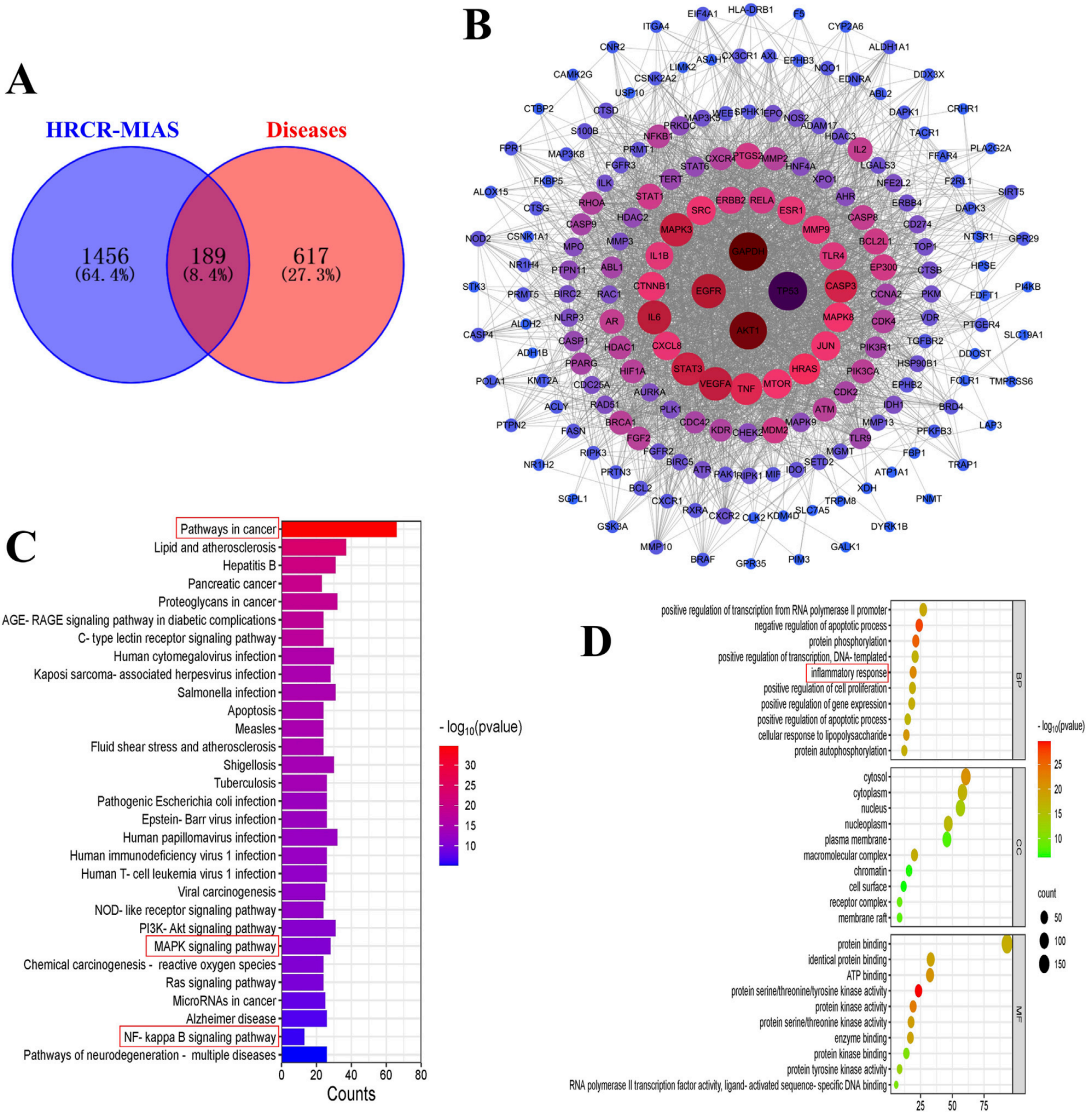
Network analysis of HRCR-MIAS in CAC. **(A)** Venn analysis of the targets of the component and disease. **(B)** PPI network diagram of the common targets. **(C)** KEGG enrichment. **(D)** GO enrichment.

**FIGURE 4:**
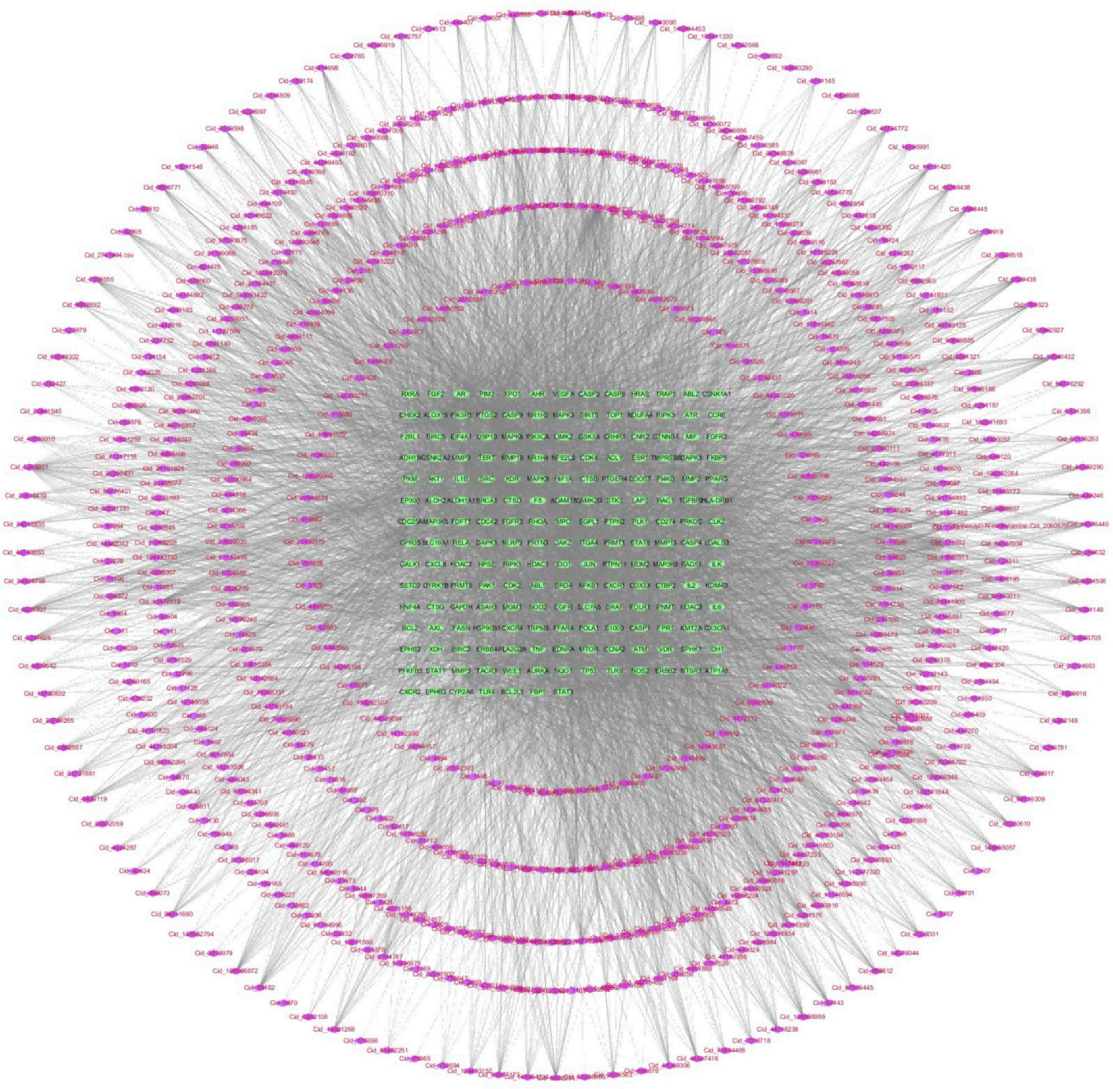
855 Components-189 targets network diagram in Network analysis.

In 855 common components of HRCR and HRCR-MIAS, 1645 targets were predicted in the SwissTarget (Supplementary Table S4). Meanwhile, 806 targets in ‘colitis-associated colorectal cancer’, ‘colorectal cancer’, and ‘colitis’ were retrieved (Supplementary Table S5). 189 common targets were acquired after Venn analysis (Figure 3A, Supplementary Table S6). Figure 3B lists the PPI interaction network of 189 common targets. The components-targets network diagram is arranged in Figure 4. The graphical results of KEGG enrichment and GO enrichment were exhibited in Figure 3C, D. Their specific content is illustrated in Supplementary Tables S7, S8. In the results of KEGG enrichment, we found that the cross-linked MAPK signaling and NF-kB signaling were the potential pathways for HRCR treatment of CAC (Figure 3C). Interestingly, previous studies have already demonstrated the role of cross-linked MAPK signaling and NF-kB signaling in CAC development (Hardwick et al., 2001; Zou et al., 2016; Lu et al., 2022). Meanwhile, the inflammatory response in GO enrichment was considered the main reason for HRCR in CAC (Figure 3D).

### 3.3 The suppressive HRCR-MIAS impact on SW620 cells

**FIGURE 5:**
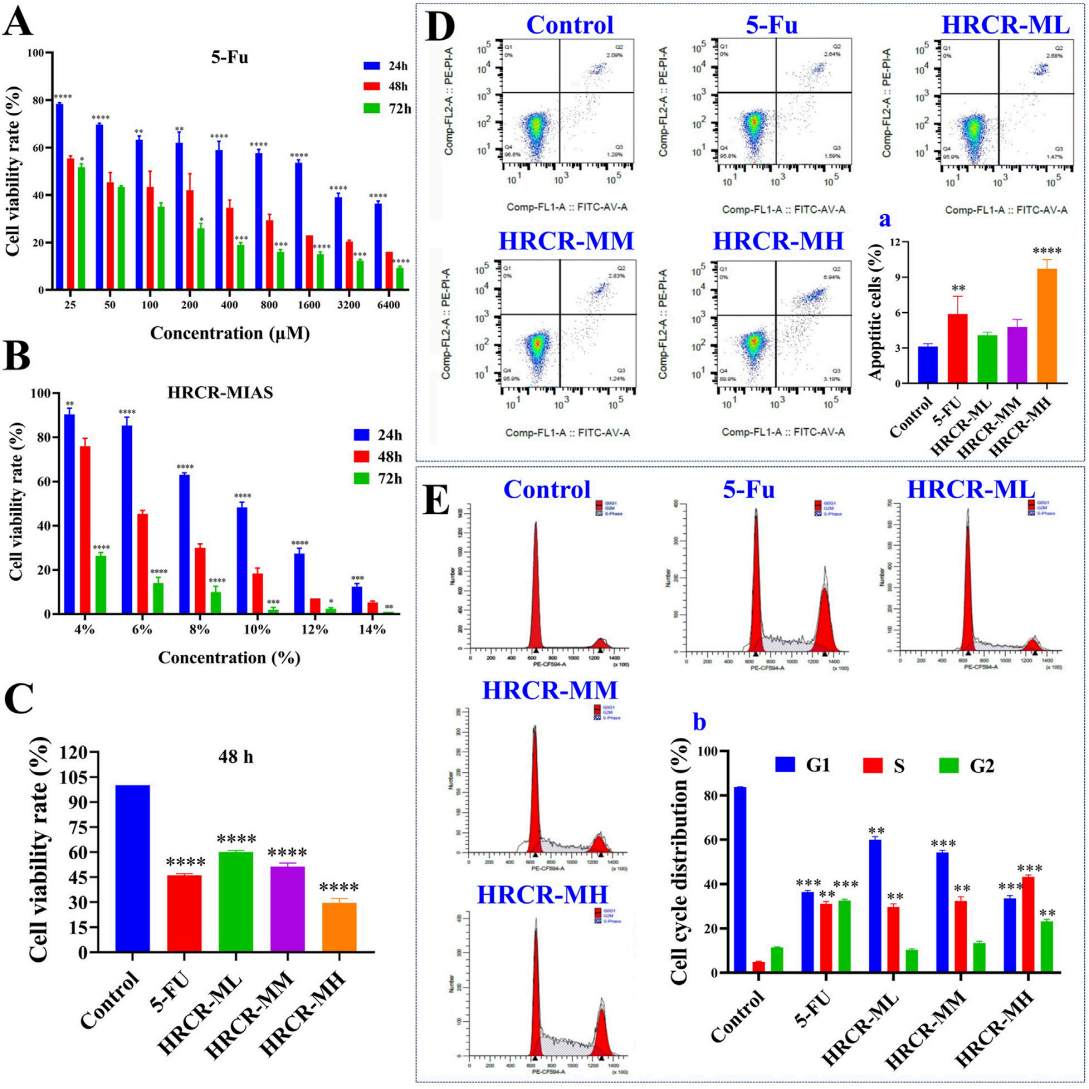
The inhibitory effect of HRCR-MIAS on SW620 cells. **(A)** The screen of the optimal intervention concentration for 5-Fu on SW620 cells after 24, 48, and 72 h of intervention. **(B)** The screen of the optimal intervention concentration for HRCR-MIAS on SW620 cells after 24, 48, and 72 h of intervention. **(C)** Cell proliferation assays. **(D)** Cell apoptosis, a is the number of apoptotic cells. **(E)** Cell cycle, b is cell cycle distribution. This experiment was performed three times in parallel. *p < 0.05, **p < 0.01, ***p < 0.001, ****p < 0.0001 vs the Control group.

**FIGURE 6:**
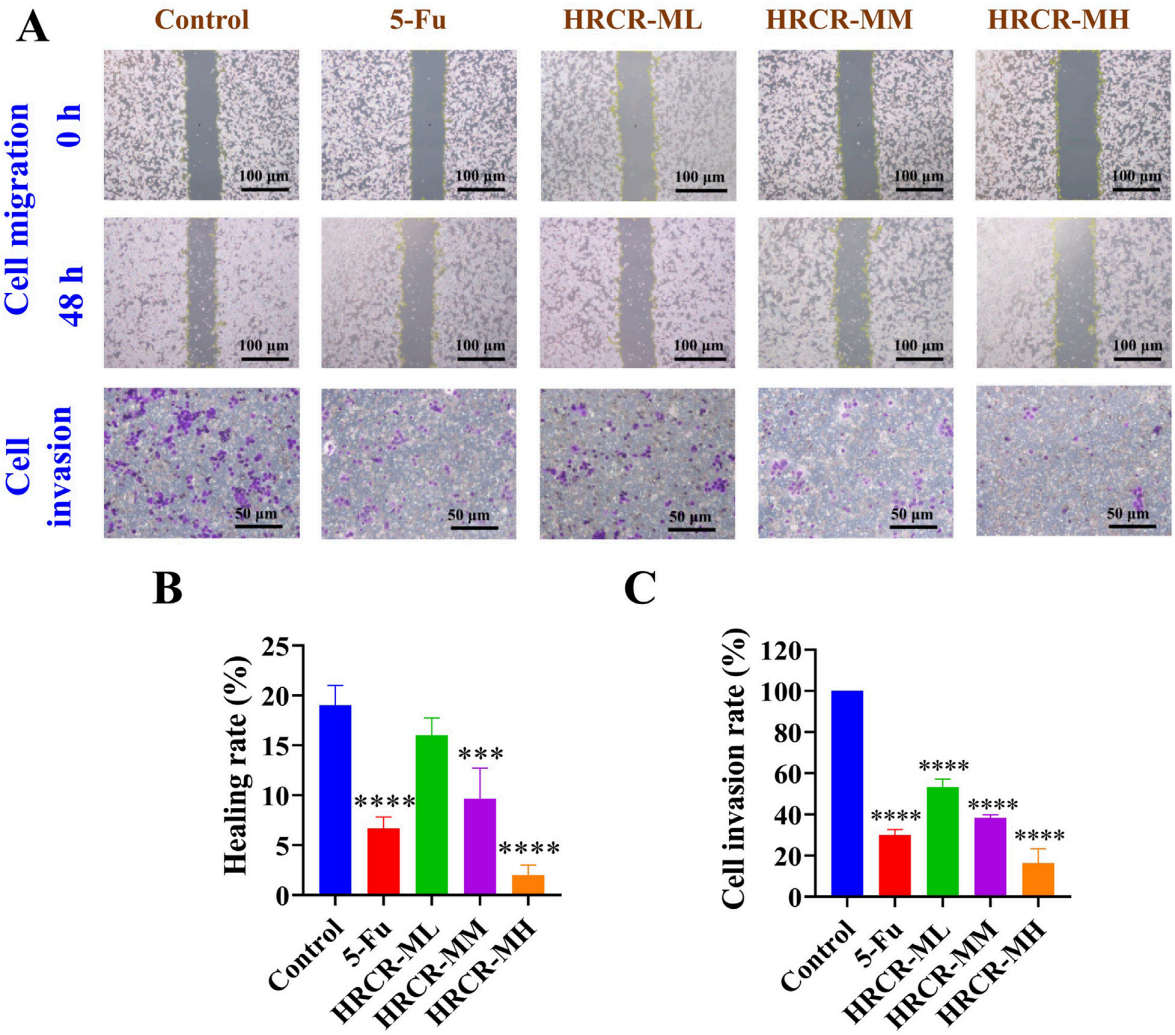
The HRCR-MIAS regulator impact on SW620 cells in the invasion and migration. **(A)** Representative diagram of cell migration and invasion (the panel magnification of cell migration is ×10, and the panel magnification of cell invasion is ×20). **(B)** Healing rate. **(C)** Cell invasion rate.

In this study, we screened the optimal intervention concentration and intervention time of 5-Fu and HRCR-MIAS on SW620 cells (Figures 5A,B). The IC_50_ values of 5-Fu acting on SW620 cells at one, two, and 3 days were 1119μM, 43.7μM, and 26μM, respectively. The IC_50_ values of HRCR acting on SW620 cells at one, two, and 3 days were 9.42%, 5.74%, and 4.01%, respectively (Figures 5A,B). The outcomes showed that 5-Fu and HRCR significantly inhibited SW620 cell proliferation at 48 h of intervention (Figure 5C). Based on the IC_50_ of 48h, 50 μM 5-Fu was selected as the positive control group. 3%, 6%, and 9% were selected as HRCR-ML, HRCR-MM, and HRCR-MH, respectively. Flow cytometry, cell scratch test, and Transwell test results showed that 5-FU and HRCR-MIAS induced apoptosis of SW620 cells (Figure 5D) and regulated cell cycle progression (Figure 5E). In the S phase, the proportion of cells arrested is elevated with increased HRCR-MIAS concentration. What’s more, the SW620 cells’ capability to migrate and invade was significantly inhibited in a way that was based on the dosage (Figures 6A–C).

### 3.4 HRCR-MIAS intervention inhibited the MAPK/NF-kB signaling pathway on SW620 cells

**FIGURE 7:**
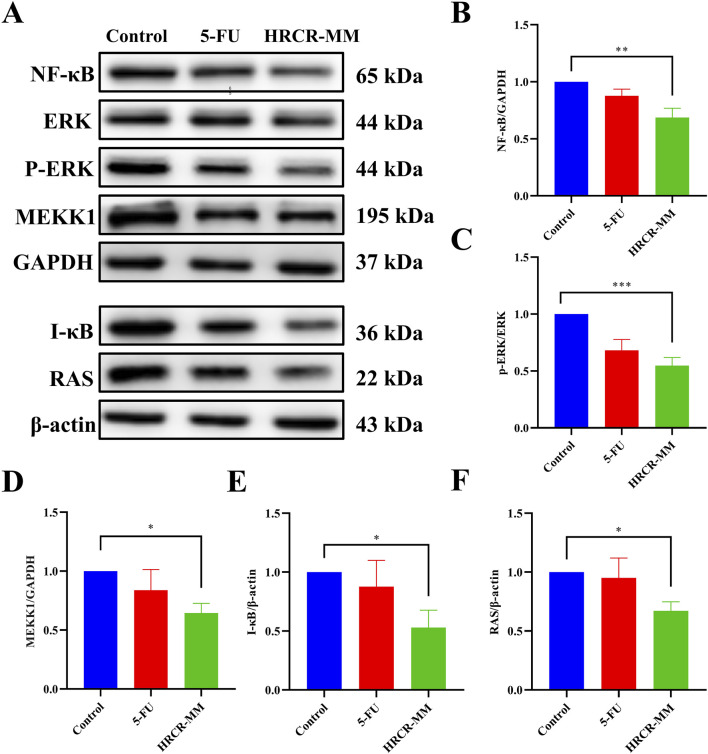
HRCR-MIAS intervention suppressed MAPK/NF-kB signaling pathway in SW620 cells. **(A)** Representative gel. Relative protein expressions of **(B)** NF-kB, **(C)** ERK/p-ERK, **(D)** MEKK1, **(E)** I-kB, and **(F)** RAS. n = 3. *p < 0.05, **p < 0.01, ***p < 0.001, ***p < 0.0001 vs the AOM/DSS group.

To additionally clarify the pathway of HRCR-MIAS inhibiting SW620 cells, the expressions of the major proteins, including RAS, MEKK1, ERK, IKB, and NF-kB in the MAPK/NF-kB signaling pathway were detected via WB. Based on the IC_50_ of 48h, we selected 50 μM-5-Fu as the positive control group and the 6% HRCR-MIAS group as the centerpiece of the mechanism study. Our outcomes exhibited that contrasted with the control group, NF-kB, ERK, MEKK1, IKB, and RAS levels were significantly decreased in the 5-FU group and the HRCR-MM group (Figures 7B–F, p < 0.001, p < 0.05, p < 0.01, p < 0.0001, p < 0.001). After HRCR-MM treatment, the NF-kB, ERK, MEKK1, IKB, and RAS expression levels were significantly inhibited in contrast with those in the 5-FU group (Figures 10B–F, p < 0.001, p < 0.05, p < 0.01, p < 0.0001, p < 0.001). This evidence supports HRCR-MIAS intervention to suppress signaling pathways of MAPK/NF-kB in SW620 cells.

### 3.5 HRCR intervention improved the general state in CAC mice

**FIGURE 8:**
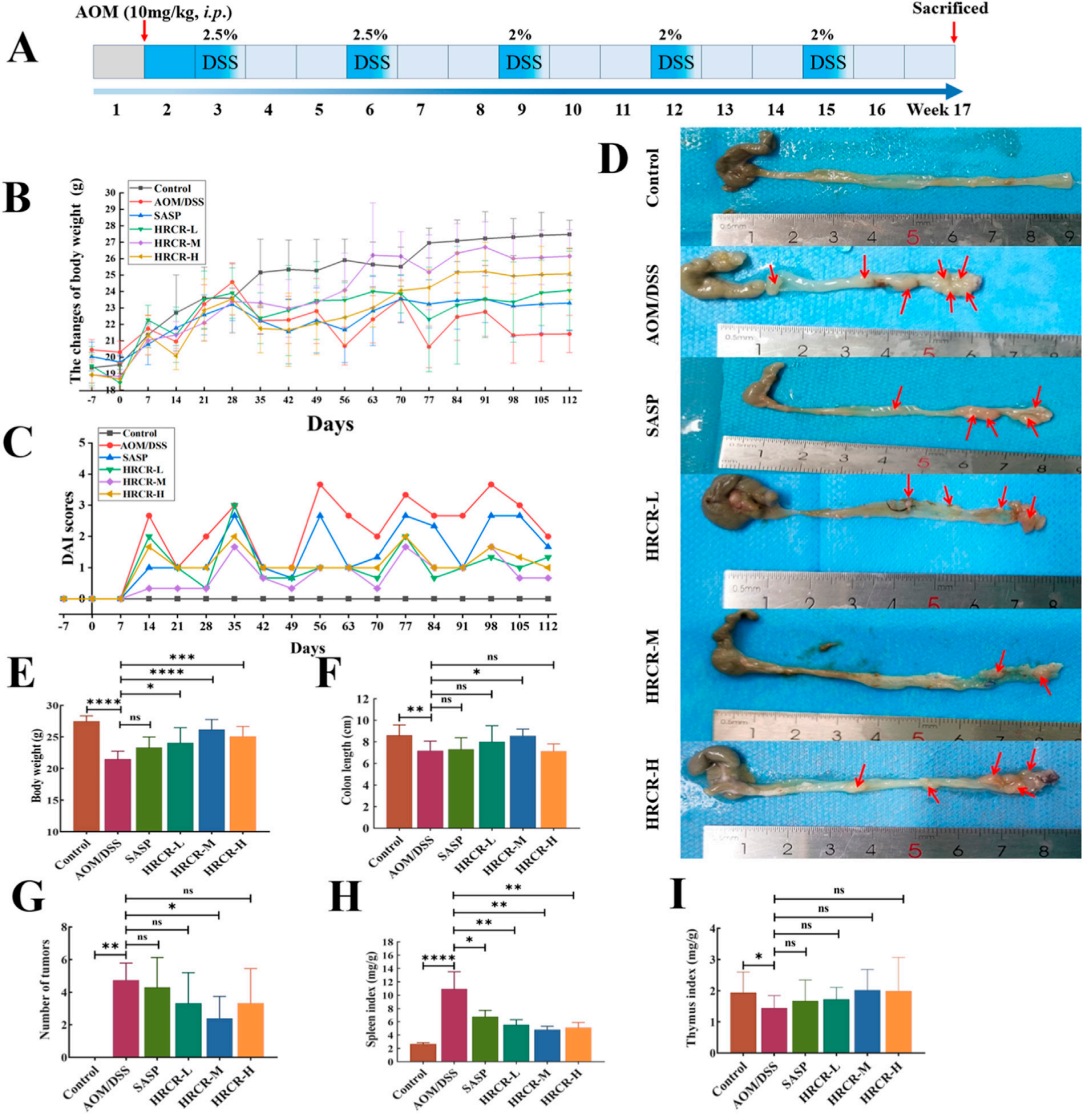
HRCR intervention improved the general state in CAC mice. **(A)** The experimental procedure in this study. **(B)** The alterations in weight of the body. **(C)** DAI scores. **(D)** Representative diagram of colorectum. **(E)** Body weight on the last day of treatment. **(F)** Colon length after sampling. **(G)** Number of tumors. **(H)** Spleen index. **(I)** Thymus index. n = 8-10. *p < 0.05, **p < 0.01, ***p < 0.001, ****p < 0.0001 vs the AOM/DSS group.

Based on the above studies, we verified the HRCR impact on CAC caused by AOM/DSS in mice *in vivo*. The alterations in weight of body and DAI scores were exhibited in Figures 8B,C. After 112 days of AOM/DSS induction, the body weight decreased significantly, and the DAI score increased significantly in mice in comparison to the other groups, which indicated the CAC mouse model was successful replication. In contrast to the AOM/DSS group, it was also found that the DAI score decreased to different extents (Figure 8C), The body weight increased significantly after intervention by low-, medium-, and high-dose groups (Figure 8C,D, *p* < 0.05, *p* < 0.0001, *p* < 0.001). Contrasted to the AOM/DSS group, the colon length was increased dramatically after HRCR-M intake (Figure 8F, *p* < 0.05), but no significant variations were identified in HRCR-H and HRCR-L groups (Figure 8F, *p* > 0.05); tumor counts in the colon were significantly reduced after HRCR-M intake (Figure 8G, *p* < 0.05), but no significant variations were identified in HRCR-H and HRCR-L groups (Figure 8G, *p* > 0.05); the spleen index was downregulated markedly after HRCR-L, HRCR-M, and HRCR-H groups intervention (Figure 8H, *p* < 0.01), whereas no significant variations were detected for the thymus index (Figure 8I, *p* > 0.05).

### 3.6 HRCR intervention recovered colonic lesions and alleviated the inflammatory process in CAC mice

**FIGURE 9:**
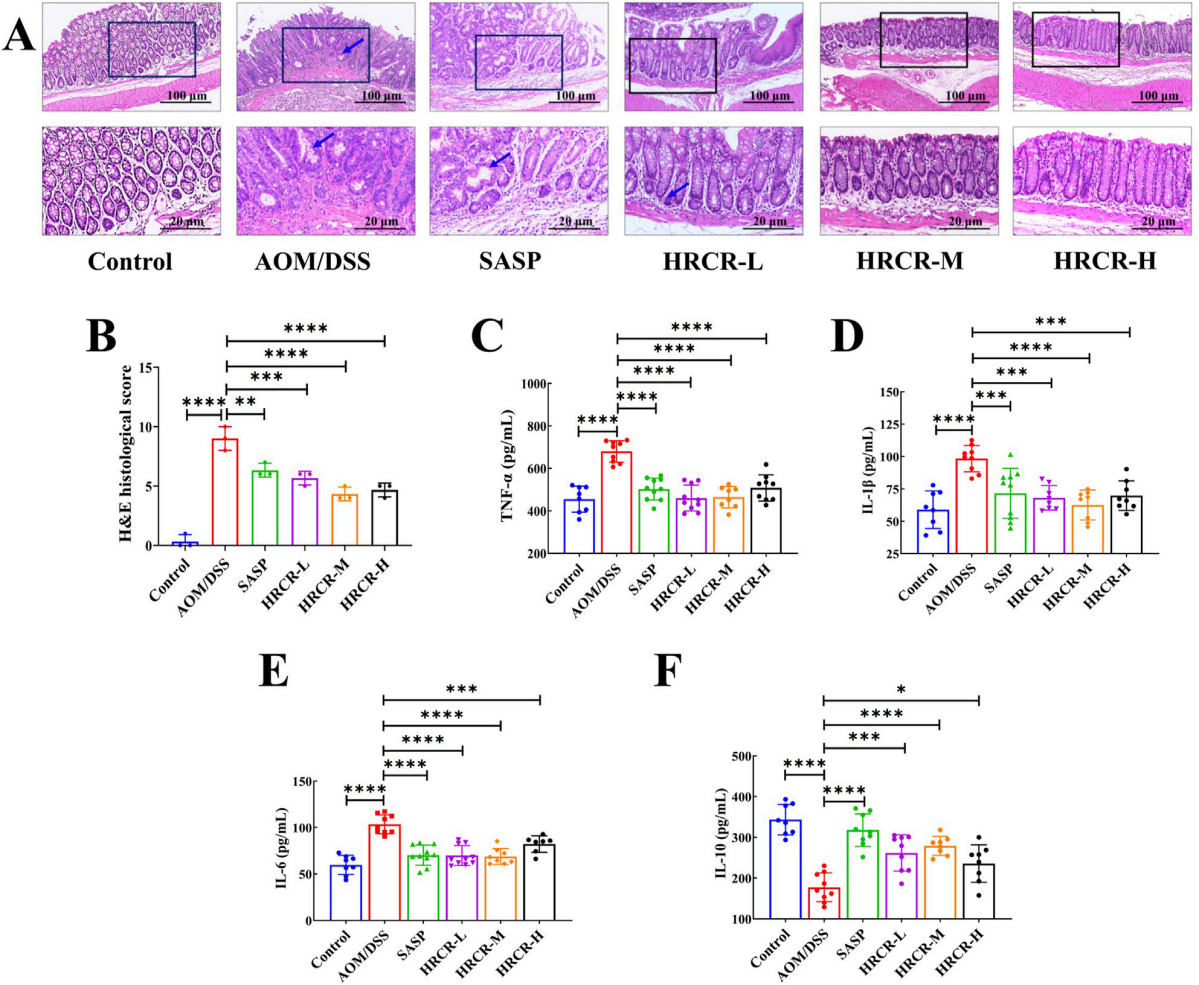
HRCR intervention recovered colonic lesions and cytokine expression in CAC mice. **(A)** Representative diagram of H&E pathological segments (Upper panel magnification is ×10 and lower panel magnification is ×40). **(B)** H&E pathological scores (n = 3). **(C)** TNF-α. **(D)**IL-1β. **(E)** IL-6. **(F)** IL-10. n = 8-10. *p < 0.05, **p < 0.01, ***p < 0.001, ****p < 0.0001 vs the AOM/DSS group.

After that, H&E pathological sections and cytokines were observed and detected. The lesions, including the defect of the crypt, goblet cell depletion, cell infiltrate, and edema, as well as cancerization such as dysplasia, were observed in the AOM/DSS group, indicating that the model of CAC induced by AOM/DSS was established successfully. We also found that contrasted to the AOM/DSS group, the lesions and cancerization of the colon were alleviated memorably (Figures 9A,B, *p* < 0.001, *p* < 0.0001, *p* < 0.0001), pro-inflammatory factors like IL-1β, IL-6, and TNF-α were downregulated obviously (Figures 9C–E, *p* < 0.001 or *p* < 0.0001), and anti-inflammatory factor, like IL-10, was upregulated noticeably (Figure 9F, *p* < 0.001, *p* < 0.0001, *p* < 0.05) after intervention by HRCR-L, HRCR-M, and HRCR-H, respectively. Our outcomes confirmed that HRCR intervention alleviated the inflammatory process in CAC mice to prevent colorectal carcinogenesis.

### 3.7 HRCR intervention restored the impaired intestinal permeability in CAC mice

**FIGURE 10:**
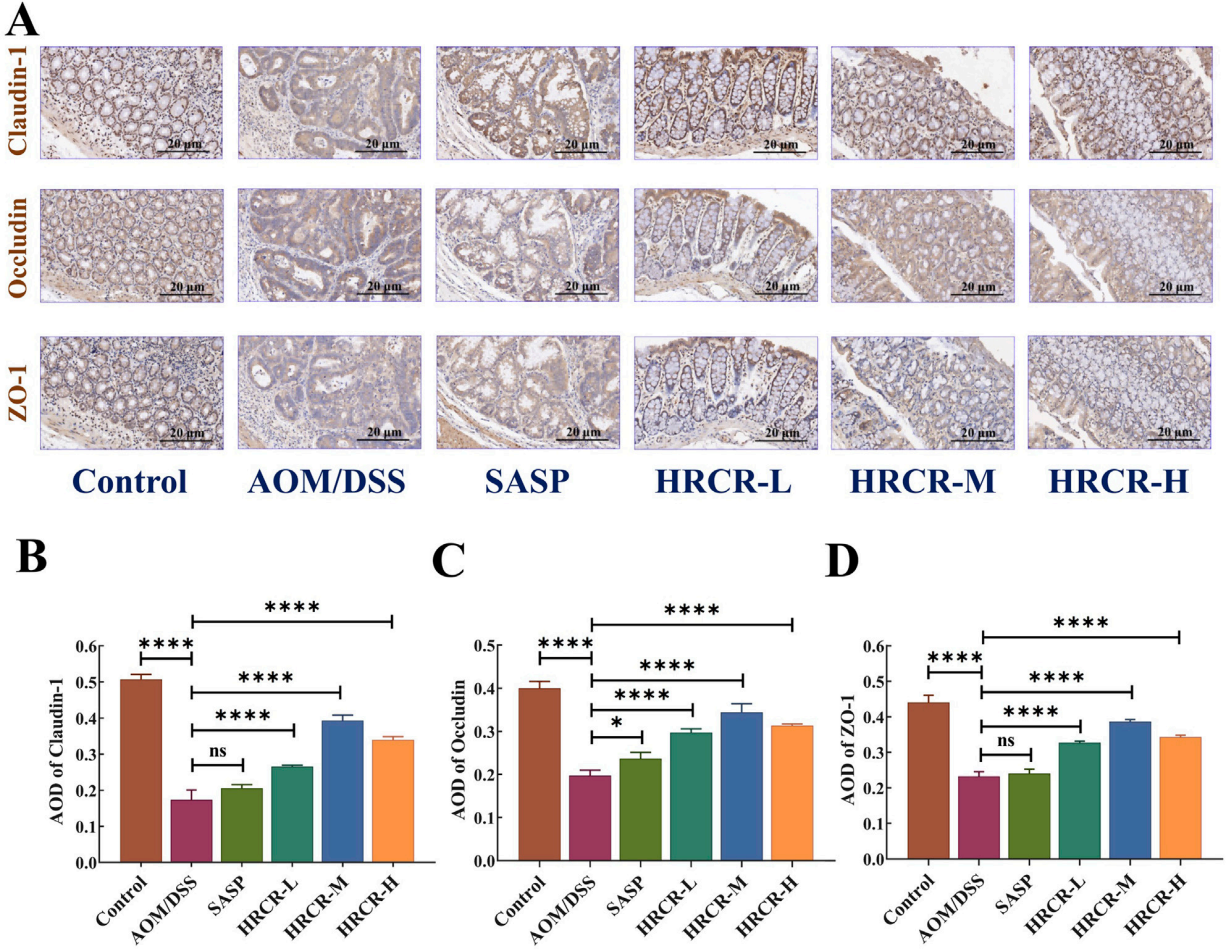
HRCR intervention restored the impaired intestinal permeability in CAC mice. **(A)** Representative diagram of IHC (Panel magnification is ×40). **(B)** AOD of Claudin-1. **(C)** AOD of Occludin. **(D)** AOD of ZO-1. n = 3. ****p < 0.0001 vs the AOM/DSS group.

The function of the mucosal barrier of the intestine and intestinal permeability is often impaired when inflammatory infiltration and inflammatory carcinoma transform into atypical dysplasia (Gadaleta et al., 2011; Landy et al., 2016). The expressions of the intestinal permeability proteins, such as Claudin-1, Occludin, and ZO-1, were determined via IHC (Figure 10A). According to the immunohistochemical results, it is not difficult to find that in AOM/DSS-induced CAC mice, the expressions of Claudin-1, Occludin, and ZO-1 were obviously downregulated. Our outcomes affirmed that intestinal permeability was impaired in the current CAC mouse model. Interestingly, the Claudin-1, Occludin, and ZO-1 expressions were significantly elevated after intervention by HRCR-L, HRCR-M, and HRCR-H in contrast to the AOM/DSS group (Figures 10B–D, *p* < 0.0001). This result indicated that HRCR intervention restored the impaired permeability of the intestine in CAC mice.

### 3.8 HRCR intervention inhibited the MAPK/NF-kB signaling pathway in CAC mice

**FIGURE 11:**
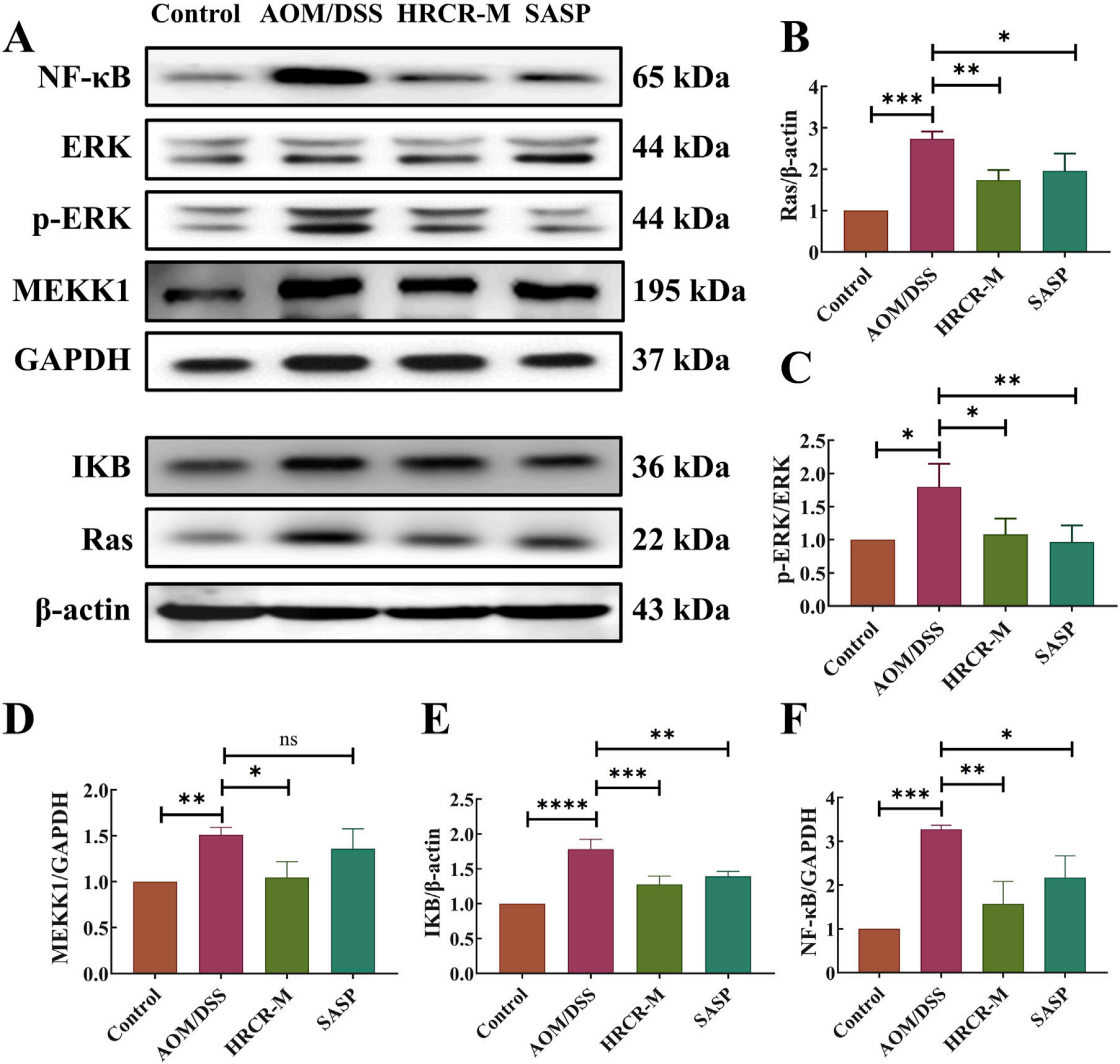
HRCR intervention inhibited CAC mice’s MAPK/NF-kB signaling pathway. **(A)** Representative gel. Relative protein expression of **(B)** RAS, **(C)** ERK/p-ERK, **(D)** MEKK1, **(E)** IkB, and **(F)** NF-kB. n = 3. *p < 0.05, **p < 0.01, ***p < 0.001, ***p < 0.0001 vs the AOM/DSS group.

To additionally elucidate the pathway of HRCR in alleviating CAC, the expressions of the major proteins, including RAS, MEKK1, ERK, IKB, and NF-kB in the ERK/NF-kB signaling pathway were detected via WB (Figure 11A). We took the optimal mid-dose (HRCR-M) measure from the above pharmacodynamic study as the centerpiece of the mechanistic study. Our outcomes revealed that the levels of RAS, MEKK1, ERK, IKB, and NF-kB in the AOM/DSS group were significantly upregulated in contrast to the control group (Figures 11B–F, *p* < 0.001, *p* < 0.05, *p* < 0.01, *p* < 0.0001, *p* < 0.001). After HRCR-M treatment, the significant suppression of the expression levels of RAS, ERK, IKB, MEKK1, and NF-kB was identified in comparison to the AOM/DSS group (Figures 11B–F, *p* < 0.001, *p* < 0.05, *p* < 0.01, *p* < 0.0001, *p* < 0.001). This evidence supported that HRCR intervention inhibited CAC mice’s MAPK/NF-kB signaling pathway.

## 4 Discussion

Colitis-associated colorectal cancer (CAC) belongs to a particular sub-group of colorectal cancers that develop in patients with IBD as a result of prolonged colitis (Dan et al., 2023). A chronic inflammatory environment occurs in the intestinal mucosa when the inflammatory factors accumulate, causing oxidative stress and DNA damage, which ultimately causes the development of CAC(Yin et al., 2023). The early control of inflammation is effective in reducing the incidence of CAC. TCM has an excellent advantage in the prophylaxis and treatment of CAC (Duan et al., 2023). This study investigated the pharmacological effects *in vitro* and *in vivo*, as well as the pathways of HRCR intervention in CAC mice.

Drug-containing intestinal absorption solution is a method to simulate the intestinal environment *in vitro* and collect the absorbed intestinal fluid for experimental studies, and this method has significant advantages in studying the effects of Chinese medicines on intestinal diseases *in vitro* (Zhang et al., 2018). Compared with commonly used biologics for the treatment of CAC, such as anti-tumor necrosis factor-α or small molecule kinase inhibitors, TCM has the advantages of multi-components, multi-targets, and fewer side effects for the treatment of CAC, and the study using HRCR-MIAS can better reflect the chemical composition of HRCR into the body, which makes up for the shortcoming of complex composition of TCM and the difficulty of direct drug delivery. In our study, we focused on the *in vitro* effects of HRCR-MIAS on colon cancer SW620 cells. In the present study, 855 common components were identified in HRCR and HRCR-MIAS (Supplementary Table S3) by UHPLC Q-Exactive-MS analysis, and 25 specific components were identified in HRCR-MIAS (Table 1). These components may be the active ingredients responsible for the pharmacological effects of HRCR. The metabolic components in the body and network analysis can be used to screen for specific targets, discover multi-target mechanisms of composite TCM, and rapidly assess the activity of Chinese medicinal compounds, which improves the accuracy of prediction results compared with the traditional composition databases (Jiashuo et al., 2022; Wang et al., 2022; Zhao L. et al., 2023). To further seek a potential mechanism for HRCR in CAC, MIAS components of HRCR and network analysis were analyzed in combination. 189 common targets were obtained after taking the intersection of component and disease targets (Figure 3A, Supplementary Table S6). They may be possible targets of HRCR in managing CAC (Liu et al., 2022). Afterward, GO and KEGG enrichment analyses were conducted employing 189 common targets. We targeted the cross-linked MAPK signaling and NF-kB signaling in KEGG enrichment and the ‘inflammatory response’ in GO enrichment as the study subjects. Although network pharmacology provides important insights for multi-target drug research, the methodological limitations of this study need to be emphasized. First, target prediction tools based on reverse docking (Swiss Target Prediction) may produce false-positive results; to address this issue, we will combine multiple tools and more experimental studies to cross-validate the prediction results in future studies to confirm our findings further.

Long-term persistent and chronic inflammation is the main reason for colorectal carcinogenesis (Shawki et al., 2018). Mobilizing the MAPK signaling pathway facilitates the activation of the NF-KB signaling pathway during the CAC development, which enhances the transcription of NF-KB target genes (Xin et al., 2021). Moreover, the NF-KB signaling pathway can also function in inflammation-related responses by activating the signaling pathway of MAPK (Piechota-Polanczyk and Fichna, 2014; Zhao et al., 2021). To investigate the efficacy of HRCR for colon cancer, we experimented with HRCR-MIAS intervention in SW620 colon malignancy cells *in vitro*. Our outcomes pinpointed that HRCR-MIAS intervention inhibited the proliferation (Figure 5C), induced the apoptosis (Figure 5D), regulated the cell cycle progression (Figure 5E), and restrained the SW620 cells ability to migrate and invade (Figures 6A–C), and significantly reduce the expression levels of MEKK1, ERK, P-ERK, RAS, I-κB, NF-κB proteins in MAPK/NF-κB signaling pathway (Figure 7). These results provided a reference for the HRCR mechanism inhibiting colon cancer cell activity and were based on the reported literature (Zheng et al., 2019; Zhou et al., 2020).

**FIGURE 12:**
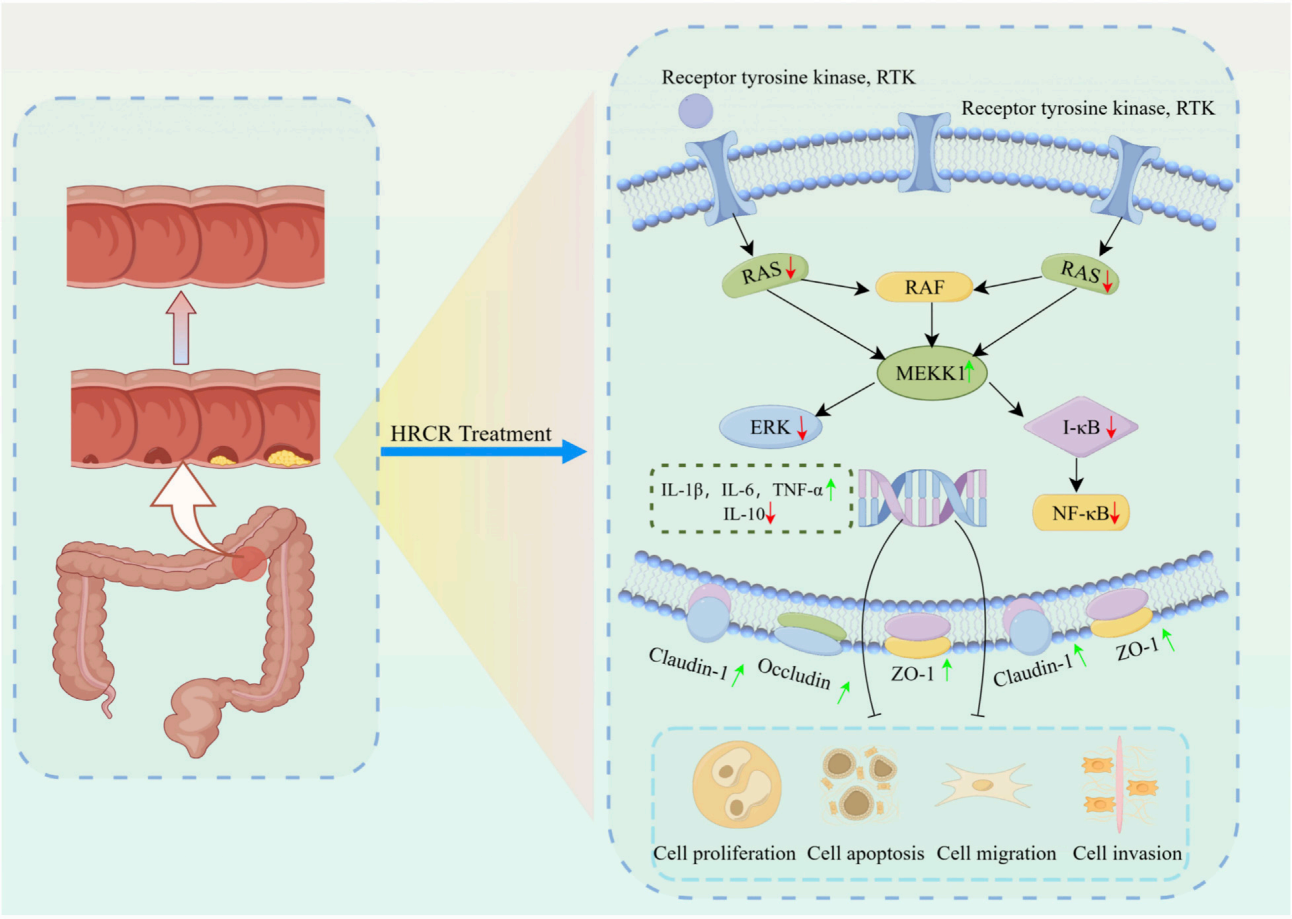
The mechanism of HRCR in CAC mice by inhibiting MAPK/NF-kB signaling cascade. The mechanism diagram was drawn at Figdraw 2.0 online drawing platform in ‘Home for Researcher’ (https://www.home-for-researchers.com). The copyright number is IAUSWf8fff.

The above molecular pathway of HRCR in CAC was further validated by *in vivo* experiments in our study. Firstly, the efficacy of low-, medium-, and high-dose in HRCR for CAC was explored. We found that the AOM/DSS group not only showed abnormal general states, including the loss of body weight, the shortened colon length, the increased tumor counts, and the abnormal changes in spleen and thymus indices, in contrast to the control group (Figure 8) but also observed the severe lesions in the colon from the results of H&E stainings (Figure 9A,B), which indicated that the CAC model mice were successfully duplicated. These outcomes aligned with previous investigations (Zhang et al., 2020; Zeng et al., 2021; Duan et al., 2023). Fortunately, the above indicators were reversed after treatment with the different doses of HRCR. Long-term aberrant expression of inflammatory factors is the risk factor for accelerating the process of colorectal inflammation and eventually inducing carcinogenesis (Yao et al., 2019; De Matteis et al., 2022). Then, the inflammatory factors were assayed in serum, and it was found that the different doses of HRCR downregulated the elevated pro-inflammation factors levels, including IL-6, IL-1β, and TNF-α (Figures 9C–E), whereas upregulated the decreased anti-inflammatory factors levels, such as IL-10 (Figure 9F). These findings confirmed that the different doses of HRCR were conducive to slowing down the inflammatory process to guard against colorectal carcinogenesis in different degrees. As previously mentioned, the transition from colonic inflammation to cancer is often accompanied by a pathologic decrease in intestinal permeability (Lavoie et al., 2020; Xu et al., 2021). Therefore, the expressions of the intestinal permeability-related proteins such as Claudin-1, Occludin, and ZO-1 were incorporated as indicators for examination in this study. It was found that the different doses of HRCR significantly elevated the decreased Claudin-1, Occludin, and ZO-1 expressions in CAC mice to varying degrees (Figure 10). Claudin-1 is a member of membrane proteins. Claudin-1, also a tight junction protein, is vital in regulating the extent of colitis and colon cancer (Bhat et al., 2020). Occludin is responsible for sealing intercellular junctions and maintaining cell permeability (Feldman et al., 2005). The loss of Occludin from tight junctions in intestinal mucosa leads to barrier loss and exacerbates the inflammatory process (Kuo et al., 2019). ZO-1 has a critical function of dissociation from tight junctions (Kuo et al., 2021). It also has a core function in the modulation of mechanosensation and force transmission at tight junctions (Haas et al., 2022). Our study affirmed that HRCR intake recovered the impaired intestinal permeability. Finally, the mechanism of HRCR in CAC mice was verified *in vivo* by western blot based on the outcomes of network analysis in combination with UHPLC Q-Exactive-MS analysis. In the MAPK/NF-kB signaling pathway, HRCR intake inhibited the expression of the essential proteins, including RAS, ERK, MEKK1, IKB, and NF-kB (Figures 11B–F). MAPK and NF-kB signaling pathways often interact with each other in cells and regulate the regulation of cells’ physiological and pathological processes (Yang et al., 2022). MAPK signaling pathway can indirectly affect the activity of the NF-kB pathway by phosphorylating and activating I-kB kinase. Conversely, NF-kB signaling modulates MAPK signaling to regulate the downstream effects by affecting the expression or activation status of MAPK kinases (Lai et al., 2017). Our results were consistent with this relationship and provided a potential pathway for HRCR in CAC therapy (Figure 12).

Our studies have explored the effects and mechanisms of HRCR in treating CAC from *in vivo* and *in vitro* perspectives, respectively, which will support our later studies; however, the key active substances of HRCR in treating CAC are still unclear and need to be further investigated. Diarylheptanoids are an important class of plant secondary metabolites, which are structurally characterized by a seven-carbon chain linked by two aromatic rings. These secondary metabolites are mainly found in Curcuma longa and have been found to have strong antitumor effects (Sudarshan et al., 2018; Sudarshan et al., 2024). On this basis, we will apply primary colon cancer cell lines and organoid models to investigate the role of HRCR in the treatment of CAC and elucidate the key active substances and related molecular mechanisms of HRCR in the treatment of CAC, with a view to providing references for clinical applications.

## 5 Conclusion

In conclusion, HRCR-MIAS’s *in vitro* effect against SW620 colon cancer cells mainly showed that it inhibited proliferation, induced apoptosis, regulated cell cycle progression, and restrained migration and invasion. Moreover, HRCR intervention not only suppresses colonic inflammation and improves intestinal permeability but also relieves CAC by suppressing the activated MAPK/NF-kB signaling cascade.

## Data Availability

The original contributions presented in the study are included in the article/Supplementary Material, further inquiries can be directed to the corresponding authors.

## Abbreviations

AR, Astragali Radix; HR, Hedysari Radix; HRPCM, Hedysari Radix Praeparata Cum Melle; VPCR, Curcumae Rhizoma (vinegar processed); HRCR, Hedysari Radix Praeparata Cum Melle and Curcumae Rhizoma (vinegar processed) herb pair; IBD, inflammatory bowel disease; CRC, colorectal cancer; CAC, colitis-associated colorectal cancer; UC, ulcerative colitis; TCM, Traditional Chinese medicine; CD, Crohn’s disease; HRCR aqueous extract solution for HRCR; Con-IAS, control intestinal absorption solution; HRCR-MIAS, medicated intestinal absorption solution of HRCR; ZO-1, zonula occludens-1; AOM/DSS, azoxymethane/dextran sulfate sodium; SPF, specific pathogen-free; SASP, salazosulfapyridine; DAI, disease activity index; HE, hematoxylin–eosin; ELISA, enzyme-linked immunosorbent assay; WB, western blot; IHC, immunohistochemical;
