# Supplementary material for: Mechanism of hedysari radix praeparata cum melle and curcumae rhizoma herb pair in colitis-associated colorectal cancer through the MAPK/NF-κB signaling pathway: an investigation in vivo and in vitro
Source: Front Chem. 2025 May 8;13:1551722. doi: 10.3389/fchem.2025.1551722 (PMC12095293; doi:10.3389/fchem.2025.1551722)
Supplement: Supplementary file 1 [file DataSheet1.docx]

Supplementary Material

1. **Methods**
2. **The preparation method of HRPCM**

A certain number of HR pieces added the refined honey infiltration for 1 h. Refined honey was diluted with distilled water (20% of honey). Approximately 25 g of refined honey was added to 100 g of HR pieces. The oven temperature was set to 70℃, the baking time was set to 2.5 h and the HR tablets were laid to a thickness of 3 cm in a rectangular porcelain plate. After baking in the oven, the tablets were removed, cooled and set aside.

1. **The** **chromatographic condition, Mass Spectrometry Conditions, and the method of operation**
   1. **The chromatographic condition**

Ultra-high performance liquid chromatography was Thermo UHPLC vanquish. ACQUITY UPLC HSS T3 (2.1 mm X 100 mm, 1.8 µm) was used as the chromatographic column. Mobile phase: A was 0.1% formic acid aqueous solution. B was 0.1% formic acid acetonitrile solution. Flow rate was was set in 0.3 mL/min. column temperature was set at 35℃. The following **Table M-S1** provided the gradient elution.

**Table M-S1丨The gradient elution program**

| Time (min) | Mobile phase A (%) | Mobile phase B (%) |
| --- | --- | --- |
| Initial | 95 | 5 |
| 17.0 | 2 | 98 |
| 17.2 | 95 | 5 |
| 20.0 | 95 | 5 |

- 1. **The Mass spectrum condition**

The mass spectrum parameters are shown in **Table M-S2.**

**Table M-S2丨**The mass spectrum parameters

| The Mass spectrum | Thermo Q-Exactive HFX mass spectrometer. | |
| --- | --- | --- |
| Collection mode | Positive and negative ion collection mode. | |
| Source parameters | Spray Voltage: 3800, -3000; Shealth Gas: 45; Aux Gas: 20; Spare Gas:0;  Capillary Temperature: 320℃; Probe Heater Temp: 370℃. | |
| **Full MS** | Resolution | Resolution |
|  | Full MS | Full MS |
| **dd-MS^2^** | Resolution | dd-MS^2^ |
|  | Loop count | 10 |
|  | TopN | 10 |

- 1. **Preparation of sample**
     1. Preparation of intestinal fluid sample

The sample of intestinal fluid was taken as 100 μL. 800 μL of methanol was added, vortexed and mixed for 60 s. The sample was allowed to stand at -20℃ for 30 min and centrifuged at 16000 g for 20 min at 4℃. The supernatant was dried under vacuum. The residue was added with 100 μL of 40% aqueous methanol, vortexed, and centrifuged at 16000 g for 15 min at 4℃. The residue was added 100 μL of 40% aqueous methanol solution, vortexed and centrifuged at 16000 g for 15 min at 4℃, and the supernatant was taken and obtained.

- - 1. Mensuration

The aqueous extract solution of HRCR, the blank intestinal absorbent sample, and the medicated intestinal absorption solution of HRCR were sucked up 6 μL each, and analyzed by LC-MS. Each sample was injected into 1 needle.

- - 1. Identification of compounds

The raw data files in .raw format were imported into proteoWizard and converted to .mzXML format.XCMS software was used for peak alignment, retention time correction and peak extraction. The XCMS software was used for peak alignment, retention time correction and peak extraction.The data extracted by XCMS were then matched with the standard spectral database for structural identification.

The match of the secondary mass spectrometry (MS2) was mainly reflected in the secondary fragmentation Score, which totaled 1. The higher the score, the more reliable the identification result. The higher the score, the more reliable the identification results. It was generally recognized that if the score was above 0.7, the identification results would be more reliable.

1. **The specific intervention procedures** **for HRCR in CAC mice**

After the end of the adaptive feeding, the control mice were intraperitoneally injected with saline, whereas the mice in the other groups were administered a 10 mg/kg dose of AOM by intraperitoneal injection on the first day of the experiment. After one week, the mice were given a 2.5%-2% (w/v) DSS aqueous solution to drink at liberty for 1 week in addition to the control group. Sterile water was then given for drinking ad libitum for 2 weeks. The specific experimental details were adjusted according to the specific trial and the whole experiment lasted for 17 weeks. All intervention mice were given 0.2 mL/20g drug by gavage once a day until the day before the end of the experiment.

1. **Tables**

**Table S1│The DAI scoring** **criterion**

| Body weight loss | Stool consistency | Presence of Gross bleeding or bloodstain | Scores |
| --- | --- | --- | --- |
| No loss | Normal | Negative | 1 |
| 1%~5% |  |  | 2 |
| 6%~10% | Loose stools | Positive | 3 |
| 11%~15% |  |  | 4 |
| Over 15% | Diarrhea | Gross rectal bleeding | 5 |

**Table S2│The evaluation criterion of the H&E histological score.**

|  | | |
| --- | --- | --- |
| State of lesion | Scores | Criteria |
| Mucosal epithelium | 0 | No mucosa inflammation |
|  | 1 | Loss of <5% of the epithelial surface |
|  | 2 | Loss of 5-10% of the epithelial surface |
|  | 3 | Loss of >10% of the epithelial surface |
| Integrity of crypts | 0 | Intact crypts |
|  | 1 | Loss of <10% crypts |
|  | 2 | Loss of 10-20% crypts |
|  | 3 | Loss of >20% of crypts |
| Cell infiltrate and edema | 0 | None |
|  | 1 | Mild |
|  | 2 | moderate |
|  | 3 | severe |
| Goblet cells depletion | 0 | Absent |
|  | 1 | Present |

**Table S3│** **Components in HRCR-MIAS identified by UHPLC Q-Exactive-MS**

|  | Compound name | PubCHEM CID |
| --- | --- | --- |
|  | Chamissonolide | Cid_10019140 |
|  | Verrucarol | Cid_10038552 |
|  | Carviolin | Cid_10040432 |
|  | Methyl 2-((3aS,4R,5S,6S)-4-hydroxy-3-(hydroxymethyl)-6-(((4-hydroxyphenyl)acetyl)oxy)-8-methyl-1-oxo-1,3a,4,5,6,7-hexahydroazulen-5-yl)prop-2-enoate | Cid_100622832 |
|  | Dihydrogingerenone D | Cid_100642075 |
|  | Wistin | Cid_10095770 |
|  | (1S,4R)-7-Isopropyl-1,4-dimethyl-2,3,3a,5,6,8a-hexahydroazulene-1,4-diol | Cid_10105632 |
|  | ONO-AE3-208 | Cid_10111831 |
|  | N-Acetyl-D-phenylalanine | Cid_101184 |
|  | N-Acetyl-D-phenylalanine | Cid_101184 |
|  | Jaeskeanadiol | Cid_10125228 |
|  | cis-Pinonic acid | Cid_10130 |
|  | 2-Formamidobenzoic acid | Cid_101399 |
|  | Propenyl-L-NIO | Cid_10198252 |
|  | 1-(3,4-Dimethoxyphenyl)-2-(ethylamino)pentan-1-one | Cid_102144588 |
|  | 2'-O-Methyladenosine | Cid_102213 |
|  | Fluoren-9-one | Cid_10241 |
|  | (-)-Kainic acid | Cid_10255 |
|  | Indole-3-carboxaldehyde | Cid_10256 |
|  | 1,2-Benzenediol, 5-[3-(acetyloxy)-7-(3,4-dihydroxyphenyl)heptyl]-3-methoxy- | Cid_102571606 |
|  | 1-Naphthalenepropanoic acid, 1,2,3,5,6,7,8,8a-octahydro-2-methyl-3-oxo- | Cid_102571644 |
|  | [5-Formyl-2-(2-hydroxypropan-2-yl)-4a,6-dimethyl-2,3,4,7,8,8a-hexahydrochromen-4-yl] acetate | Cid_102571648 |
|  | 3-(Hepta-1,3-dienyl)adipic acid | Cid_102571693 |
|  | O6, N-Diacetylnorcodeine | Cid_102591297 |
|  | 3-Furoic acid | Cid_10268 |
|  | Thymoquinone | Cid_10281 |
|  | Methylsuccinic acid | Cid_10349 |
|  | Ile-Tyr | Cid_103870710 |
|  | (E)-3-(3,4-Dimethoxyphenyl)-N-homoveratrylacrylamide | Cid_1042026 |
|  | Phe-Thr | Cid_10445608 |
|  | Garcinoic acid | Cid_10455173 |
|  | Suberic acid | Cid_10457 |
|  | Rhyncophylline | Cid_10475115 |
|  | 2-(4-Allyl-2,6-dimethoxyphenoxy)-1-(3,4,5-trimethoxyphenyl)propan-1-ol | Cid_10477119 |
|  | Pyridoxine | Cid_1054 |
|  | Stanolone | Cid_10635 |
|  | Parietin | Cid_10639 |
|  | Parietin | Cid_10639 |
|  | 1H-Indole-3-ethanol | Cid_10685 |
|  | o-Xylorcinol | Cid_10697 |
|  | Hovetrichoside C | Cid_10789705 |
|  | (+)-catechin hydrate | Cid_107957 |
|  | Daidzin | Cid_107971 |
|  | Mefrosol | Cid_108312 |
|  | 3-Deoxy-D-glycero-D-galacto-2-nonulosonic acid | Cid_10869261 |
|  | .alpha.,.beta.-Trehalose | Cid_10871590 |
|  | Sucrose 6-benzoate | Cid_10906400 |
|  | Artemisinate | Cid_10922465 |
|  | 3-Isobutylglutaric acid | Cid_10976240 |
|  | 5-Aminohexylamine | Cid_11007871 |
|  | Spermidine | Cid_1102 |
|  | (3.beta.,6.alpha.,12.beta.,20Z)-3,12-Dihydroxydammara-20(22),24-dien-6-yl 2-O-(6-deoxy-.alpha.-L-mannopyranosyl)-.beta.-D-glucopyranoside | Cid_110208996 |
|  | [(3aR,4S,6R,11aR)-6,9-dihydroxy-6,10-dimethyl-3-methylidene-2,7-dioxo-3a,4,5,8,9,11a-hexahydrocyclodeca[b]furan-4-yl] 2-methylprop-2-enoate | Cid_110281983 |
|  | Graveolide | Cid_11043090 |
|  | 2'-Hydroxy-4'-methoxyacetophenone | Cid_11092 |
|  | 3-Ureidopropionic acid | Cid_111 |
|  | Succinic acid | Cid_1110 |
|  | .alpha.,.beta.-glucooctanoic .gamma.-lactone | Cid_111248 |
|  | bk-EABDI | Cid_112477320 |
|  | vitamin B1 | Cid_1130 |
|  | N-(2-Hydroxyethyl)-3-nitro-1H-1,2,4-triazole-1-acetamide | Cid_113639 |
|  | Mellein | Cid_114679 |
|  | Encecalin | Cid_114703 |
|  | Liquiritigenin | Cid_114829 |
|  | Stachydrine | Cid_115244 |
|  | Pro-Pro | Cid_11622593 |
|  | Isopetasol | Cid_11746594 |
|  | Gentianose | Cid_117678 |
|  | Antibiotic NFAT 133 | Cid_11778214 |
|  | Vanillin | Cid_1183 |
|  | (1aR,2S,2aS,5R,5aS,6S,7aS)-2-(Acetyloxy)-5-hydroxy-2a,7a-dimethyl-5-(propan-2-yl)decahydroazuleno[5,6-b]oxiren-6-yl (2Z)-2-methylbut-2-enoate | Cid_11876148 |
|  | Ilicic acid | Cid_11876195 |
|  | 5-Fluoro ABICA | Cid_118796498 |
|  | 9-.beta.-D-Ribofuranosylxanthine | Cid_1189 |
|  | 4-Hydroxymephenytoin | Cid_119507 |
|  | Veliparib | Cid_11960529 |
|  | 4-Hydroxy-3-methoxyphenyl .beta.-D-glucopyranoside | Cid_11962143 |
|  | Isocitric acid | Cid_1198 |
|  | Dihydrophaseic acid | Cid_11988273 |
|  | Dihydrophaseic acid | Cid_11988273 |
|  | (-)-Myrtenal | Cid_1201529 |
|  | Carbamylglutamic acid | Cid_121396 |
|  | Nortramadol | Cid_12149038 |
|  | 5-Fluoro ADB-PINACA | Cid_121491656 |
|  | D-Arabinonic acid | Cid_122045 |
|  | (2E)-5-Hydroxyundec-2-enoic acid | Cid_12227668 |
|  | U 80278A | Cid_122334 |
|  | Fustin | Cid_12310641 |
|  | (3aS,6S,7R,8aR)-8-Hydroxy-6,8-dimethyl-3-methylidenehexahydro-3'H-spiro[cyclohepta[b]furan-7,2'-furan]-2,5'(3H,4'H)-dione | Cid_12314731 |
|  | Propylurofuranic acid | Cid_123979 |
|  | Fraxinellone | Cid_124039 |
|  | Glu-Leu | Cid_124433150 |
|  | Thr-Leu | Cid_124433152 |
|  | N-(5-Chloro-2-pyridyl)-5-propylthiophene-3-carboxamide | Cid_1246828 |
|  | 6-O-((2Z)-3-(4-Hydroxy-3-methoxyphenyl)prop-2-enoyl)-.beta.-D-fructofuranosyl 6-O-acetyl-.alpha.-D-glucopyranoside | Cid_125416603 |
|  | 5'-Adenylic acid, 2'-deoxy- | Cid_12599 |
|  | 4-Formylphenol | Cid_126 |
|  | L-Homoserine | Cid_12647 |
|  | Dodecanedioic acid | Cid_12736 |
|  | Dodecanedioic acid | Cid_12736 |
|  | Isoflupredone | Cid_127516 |
|  | Galactonic acid | Cid_128869 |
|  | 1,2,3-Cyclohexanetriol, 5-(1,3-heptadien-1-yl)- | Cid_128964467 |
|  | (E)-3,10-Dihydroxy-4,9-dimethyldodec-6-enedioic acid | Cid_129008872 |
|  | 1'R,3'S,5'R,8'S,2E,4E)-Dihydrophaseic acid 3'-O-.beta.-D-glucopyranoside | Cid_129008896 |
|  | (2E,4E)-5-(3,8-Dihydroxy-1,5-dimethyl-7-oxo-6-oxabicyclo[3.2.1]octan-8-yl)-3-methylpenta-2,4-dienoic acid | Cid_129008948 |
|  | 2-(Hydroxy(7a-hydroxy-3-oxo-5-(propan-2-yl)octahydro-2-benzofuran-4-yl)methyl)prop-2-enoic acid | Cid_129008965 |
|  | 1-O-((2E,4E)-9-Carboxy-8-hydroxy-2,7-dimethylnona-2,4-dienoyl)hexitol | Cid_129008967 |
|  | 8-Hydroxy-3-(7-hydroxyheptyl)-6-oxo-3,4-dihydroisochromene-7-carboxylic acid | Cid_129008974 |
|  | Methyl (9Z,14Z)-12,13,16-trihydroxyoctadeca-9,14-dienoate | Cid_129008998 |
|  | 12,15-Octadecadienoic acid, 7,9,10-trihydroxy- | Cid_129008999 |
|  | 12,15-Octadecadienoic acid, 7,9,10-trihydroxy- | Cid_129008999 |
|  | Ethyl 4-O-(4-carboxy-3-hydroxy-3-methylbutanoyl)-.beta.-D-glucopyranoside | Cid_129009008 |
|  | (-)-Tamsulosin | Cid_129211 |
|  | Symplocosin | Cid_129316934 |
|  | Koninginin D | Cid_12971673 |
|  | 3-Aminopropyl(cyclohexylmethyl)phosphinic acid | Cid_130022 |
|  | Ala-Tyr | Cid_13017548 |
|  | Auranthine | Cid_130919 |
|  | Cimetidine amide | Cid_131743 |
|  | 4-Oxododecanedioic acid | Cid_13213508 |
|  | Aegineoside | Cid_132353271 |
|  | L-NG-Monomethylarginine | Cid_132862 |
|  | Ivabradine | Cid_132999 |
|  | PyroGlu-Pro | Cid_13306588 |
|  | Soyasaponin II | Cid_13326381 |
|  | 3-Hydroxypicolinic acid | Cid_13401 |
|  | Trigonelline hcl | Cid_134606 |
|  | (3Z,6E)-2,2,6-trimethyl-12-oxabicyclo[8.2.1]trideca-3,6,10(13)-triene-5,11-dione | Cid_134723072 |
|  | Prespatane | Cid_134745718 |
|  | 4-Hydroxybenzoic acid | Cid_135 |
|  | 3',5'-Cyclic GMP | Cid_135398570 |
|  | Guanosine | Cid_135398635 |
|  | Guanosine | Cid_135398635 |
|  | Hypoxanthine | Cid_135398638 |
|  | Inosine | Cid_135398641 |
|  | Inosine | Cid_135398641 |
|  | Folinic acid | Cid_135403648 |
|  | 2-o-methylguanosine | Cid_135406950 |
|  | 2-[2-(2-Quinolinyl)ethenyl]phenol | Cid_135411330 |
|  | AL 34662 | Cid_135453290 |
|  | 3-(1H-benzimidazol-2-yl)-2H-indazole | Cid_135464491 |
|  | N,N-Dimethylguanosine | Cid_135501639 |
|  | 6-Isopropylpyrimidin-4-ol | Cid_135591005 |
|  | TC-E 5008 | Cid_13590964 |
|  | Cyclic GMP | Cid_136239427 |
|  | 2'-Deoxyadenosine | Cid_13730 |
|  | Cyclopropylacetic acid | Cid_138440 |
|  | Ethyl 4-bromo-3,5-dimethyl-1H-pyrrole-2-carboxylate | Cid_138486 |
|  | Shanziside methyl ester | Cid_13892722 |
|  | Isoflavone base + 1O, 1MeO, O-Hex+C7H12NO | Cid_139292014 |
|  | Isoflavanone base + 3O, O-Hex | Cid_139292064 |
|  | N-Fructosyl pyroglutamate | Cid_139292107 |
|  | N-fructosyl isoleucine | Cid_139292115 |
|  | Feruloyl Lactate | Cid_139292180 |
|  | PyroGlu-Phe | Cid_13961383 |
|  | Odoratin | Cid_13965473 |
|  | Odoratin | Cid_13965473 |
|  | Galdosol | Cid_13966127 |
|  | Epipinoresinol-4,4'-di-O-.beta.-D-glucopyranoside | Cid_13991182 |
|  | 5,9-Dihydroxy-5,7,7-trimethyl-4,5a,6,8,8a,9-hexahydro-1H-azuleno[5,6-c]furan-3-one | Cid_13995409 |
|  | 8-Hydroxy-5,8a-dimethyl-3-methylidene-3a,4,4a,8,9,9a-hexahydrobenzo[f][1]benzofuran-2,7-dione | Cid_14021268 |
|  | a-Santal-10-en-12-oic acid | Cid_14059029 |
|  | 2-Acetylpyrrole | Cid_14079 |
|  | Benzyl alcohol + hex-pen | Cid_14079044 |
|  | 2-(3,4-Dihydroxyphenyl)-5,7-dihydroxy-3,4-dihydro-2H-chromen-3-yl .beta.-D-glucopyranoside | Cid_14104308 |
|  | Curdione | Cid_14106072 |
|  | 4-(Hexopyranosyloxy)-3-methoxybenzoic acid | Cid_14132336 |
|  | Benzoic acid + 1o, 2meo, o-hex | Cid_14132345 |
|  | 10-Hydroxy-1,6-dimethyl-9-(propan-2-yl)-5,12-dioxatricyclo[9.1.0.04,6]dodecan-8-yl (2E)-3-phenylprop-2-enoate | Cid_14155777 |
|  | 9-Phenyl-1-(2,4,6-trihydroxyphenyl)nonan-1-one | Cid_14186891 |
|  | (9a-Hydroxy-3,8a-dimethyl-5-methylidene-2-oxo-4,4a,6,7,8,9-hexahydrobenzo[f][1]benzofuran-8-yl) acetate | Cid_14258971 |
|  | Urospermal | Cid_14287067 |
|  | 3a,8-Dihydroxy-3,5a,9-trimethyl-4,5,6,7,8,9b-hexahydro-3H-naphtho[6,5-d]furan-2-one | Cid_14312974 |
|  | 9,13,15-Trihydroxy-5-methyl-4-oxabicyclo[10.4.0]hexadeca-1(12),13,15-triene-3,11-dione | Cid_14314902 |
|  | Loliolide | Cid_14334 |
|  | 6-Hydroxy-5-methoxyflavone | Cid_14349485 |
|  | 4-(.beta.-D-Glucopyranosyloxy)benzeneacetic acid | Cid_14352568 |
|  | Hannokinol | Cid_14427394 |
|  | 3,8a-Dimethyl-5-methylene-4a,6,7,8-tetrahydro-4H-benzo[f]benzofuran-2-one | Cid_14448072 |
|  | 1-(3,4-Dihydroxyphenyl)-2,3-dimethyltetralin-6,7-diol | Cid_14462042 |
|  | Valylphenylalanine | Cid_144746 |
|  | 2',6'-Glycoloxylidide | Cid_14480057 |
|  | 3-(4-Hydroxyphenyl)-3-oxopropanoic acid | Cid_14496584 |
|  | Fenchone | Cid_14525 |
|  | 3,7-Dihydroxy-9,11-eremophiladien-8-one | Cid_14543486 |
|  | L-Proline | Cid_145742 |
|  | Coumaroyl + c6h9o8 (isomer of 844, 845, 846) | Cid_145994453 |
|  | Galangin-5,7-dimethyl ether | Cid_14606540 |
|  | Galangin-5,7-dimethyl ether | Cid_14606540 |
|  | Oroxyloside | Cid_14655552 |
|  | [(E)-3-Acetyloxy-6,7-dihydroxy-7-(6-oxo-2,3-dihydropyran-2-yl)hept-4-en-2-yl] acetate | Cid_14757875 |
|  | Platyphylloside | Cid_14833392 |
|  | .beta.-Resorcylic acid | Cid_1491 |
|  | Aloin | Cid_14989 |
|  | 3-Methylisoleucine | Cid_15042684 |
|  | Pentalenolactone D | Cid_15127669 |
|  | Altamisic acid | Cid_15143694 |
|  | Pro-Val | Cid_152307 |
|  | N-[4-(2-Aminoethyl)phenyl]benzenesulfonamide | Cid_15281645 |
|  | 5-Allylvanillin | Cid_1533806 |
|  | Cinnarizine | Cid_1547484 |
|  | Coniferyl alcohol | Cid_1549095 |
|  | DL-Laudanosine | Cid_15548 |
|  | Atractylenolide III | Cid_155948 |
|  | Methyl ursolate acetate | Cid_15598285 |
|  | ((1S,5Z,9R,10R)-6-(Hydroxymethyl)-10-methyl-2-methylidene-7-oxobicyclo[7.2.0]undec-5-en-10-yl)methyl acetate | Cid_15601259 |
|  | Soyasaponin V | Cid_15608234 |
|  | Lactarolide A | Cid_156145 |
|  | Ligustrazine hcl | Cid_156709 |
|  | Tetranor-12(S)-HETE | Cid_15730832 |
|  | Scropolioside F | Cid_15736670 |
|  | Undecanedioic acid | Cid_15816 |
|  | Nepsilon,nepsilon,nepsilon-trimethyllysine | Cid_159659 |
|  | Butyl terephthalate | Cid_16066 |
|  | Hemiphloin | Cid_160711 |
|  | Maesopsin | Cid_160803 |
|  | Glycan 1,4-D-Xylobiose | Cid_160873 |
|  | 1-O-((2E,4E)-9-Carboxy-8-hydroxy-2,7-dimethylnona-2,4-dienoyl)-.beta.-D-glucopyranose | Cid_16095194 |
|  | L-Kynurenine | Cid_161166 |
|  | N6-threonylcarbamoyladenosine | Cid_161466 |
|  | 3-Hydroxyacetaminophen | Cid_161950 |
|  | (3E)-4-(1-Hydroxy-2,2,6-trimethyl-4-oxocyclohexyl)but-3-en-2-yl hexopyranoside | Cid_16196969 |
|  | 3,3-Diphenyl-L-alanine | Cid_162977 |
|  | 4',6-Dihydroxy-3'-methoxyaurone | Cid_16376440 |
|  | (1S,5S)-2,4,9-Trimethyl-6-[(E)-prop-1-enyl]-7-oxabicyclo[3.3.1]non-3-ene | Cid_16395092 |
|  | ((1S)-5-Methyl-1-(4-methylpent-3-en-1-yl)-2-oxabicyclo[2.2.2]octan-4-yl)methanol | Cid_16395956 |
|  | [(9R)-8-Isopropyl-2,4,9-trimethyl-7-oxabicyclo[3.3.1]non-2-en-5-yl]methanol | Cid_16395991 |
|  | Phyllodulcin | Cid_164615 |
|  | Carabrone | Cid_164879 |
|  | Isosulochrin | Cid_16681742 |
|  | 3-Methoxytyrosine | Cid_1670 |
|  | (+)-Carvone | Cid_16724 |
|  | Nardosinon | Cid_168136 |
|  | N4-acetylsulfadimethoxine | Cid_168167 |
|  | Argininosuccinic acid | Cid_16950 |
|  | Hypolide | Cid_173273 |
|  | (-)-Caryophyllene oxide | Cid_1742210 |
|  | Malioxamycin | Cid_175396 |
|  | 3,4',5-Trihydroxy-7-methoxyflavanone | Cid_181132 |
|  | Maltose | Cid_181526 |
|  | Ageratriol | Cid_181557 |
|  | 4-Acetamidobutanoate | Cid_18189 |
|  | Benahorin | Cid_182169 |
|  | Gly-Phe | Cid_182174 |
|  | Asn-Pro-Arg | Cid_18219245 |
|  | Gln-Glu-Lys | Cid_18220610 |
|  | N-Acetyl-L-glutamate | Cid_185 |
|  | N-[2-(1H-Imidazol-5-yl)ethyl]-N'-(2-phenylethyl)thiourea | Cid_1858147 |
|  | 2-Ethyl-1,2,3,4-tetrahydro-5-isoquinolinamine | Cid_18687359 |
|  | Cirsimaritin | Cid_188323 |
|  | His-Leu | Cid_189008 |
|  | Loureirin b | Cid_189670 |
|  | Cyclopropyl(3-pyridyl)methanol | Cid_19374862 |
|  | Phellamurin | Cid_193876 |
|  | 3-[(3-Butyl-6-methoxy-2-methyl-4-quinolyl)thio]propionitrile | Cid_1942420 |
|  | Adipic acid | Cid_196 |
|  | 2-[(4-Amino-6-anilino-1,3,5-triazin-2-yl)methoxy]benzaldehyde | Cid_1972117 |
|  | Agmatine | Cid_199 |
|  | Cyclopentaneacetic acid, 1-phenyl- | Cid_19966045 |
|  | Haploside d | Cid_20106108 |
|  | Butyrophenone, 2-piperidino- | Cid_20461540 |
|  | Asarylaldehyde | Cid_20525 |
|  | N-(4-Ethylbenzyl)-N-methylamine | Cid_2060575 |
|  | Eglumetad | Cid_213056 |
|  | 5-Hydroxy-7-(4-hydroxy-3-methoxyphenyl)-1-(4-hydroxyphenyl)heptan-3-one | Cid_21588208 |
|  | 2-(2-Carbomethoxy-3-hydroxy-5-methylphenoxy)-5-hydroxy-3-methoxybenzoic acid | Cid_21629518 |
|  | Zizanoic acid | Cid_21764437 |
|  | Innuchenenolide C | Cid_21769986 |
|  | Anabasine | Cid_2181 |
|  | Fumagillol | Cid_222778 |
|  | 3,4-Dimethylbenzaldehyde | Cid_22278 |
|  | 4-[3,5-Dihydroxy-7-(4-hydroxyphenyl)heptyl]pyrocatechol | Cid_22297431 |
|  | 4-[3,5-Dihydroxy-7-(4-hydroxyphenyl)heptyl]pyrocatechol | Cid_22297431 |
|  | Licoagroside B | Cid_22297602 |
|  | Licoagroside B | Cid_22297602 |
|  | Linalool oxide | Cid_22310 |
|  | 3-Hydroxysuberic acid | Cid_22328017 |
|  | Phenylalanine, 3-methyl- | Cid_225441 |
|  | Fludrocortisone acetate | Cid_225609 |
|  | Azelaic acid | Cid_2266 |
|  | N-Acetylvaline | Cid_227752 |
|  | N-Acetylvaline | Cid_227752 |
|  | 2-(3-Methylbenzyl)succinic acid | Cid_228104 |
|  | Asp-Val | Cid_22824556 |
|  | Asp-Val | Cid_22824556 |
|  | Bisphenol G | Cid_228537 |
|  | 3-Cyclopentylalanine | Cid_229579 |
|  | DL-Arginine | Cid_232 |
|  | Melilotocarpan B | Cid_23259931 |
|  | 5-Ethyl-5-isopropylhydantoin | Cid_234513 |
|  | Talopram | Cid_23573 |
|  | 2,3-Dimethyl-6-quinoxalinecarboxylic acid | Cid_236268 |
|  | Lotaustralin | Cid_23638286 |
|  | 3-(4-Methoxyphenyl)-4-oxo-4H-chromen-7-yl 6-O-(carboxyacetyl)-.beta.-D-glucopyranoside | Cid_23724663 |
|  | (3E)-4-((1S)-1-Hydroxy-2,6,6-trimethyl-4-oxocyclohex-2-en-1-yl)but-3-en-2-yl .beta.-D-glucopyranoside | Cid_23757167 |
|  | (E)-5-(3-Hydroxy-2,3-dimethylnorbornan-2-yl)-2-methylpent-2-enoic acid | Cid_23786265 |
|  | 4-((2S,3S)-3-(Hydroxymethyl)-5-((1E)-3-hydroxyprop-1-en-1-yl)-7-methoxy-2,3-dihydro-1-benzofuran-2-yl)-2-methoxyphenyl hexopyranoside | Cid_23786437 |
|  | 4-((2S,3S)-3-(Hydroxymethyl)-5-((1E)-3-hydroxyprop-1-en-1-yl)-7-methoxy-2,3-dihydro-1-benzofuran-2-yl)-2-methoxyphenyl hexopyranoside | Cid_23786437 |
|  | 1H-3,9a-Methanocyclopent[c]oxocin-4-carboxylic acid, octahydro-4-hydroxy-7-(2-hydroxy-1-methylethylidene)- | Cid_23815256 |
|  | 2,3-Dimethyloxirane-2-carboxylic acid [(1S,2R,4aR,8aR)-1-hydroxy-7-isopropylidene-6-keto-1,4a-dimethyldecalin-2-yl] ester | Cid_23815419 |
|  | Cumanin | Cid_23844091 |
|  | 6-O-Hexopyranosyl-1-O-(19-hydroxy-28-oxo-3-(pentopyranosyloxy)olean-12-en-28-yl)hexopyranose | Cid_23846524 |
|  | Acremin F | Cid_23846553 |
|  | (11E,15Z)-9,10,13-Trihydroxy-11,15-octadecadienoic acid | Cid_23872026 |
|  | 4-Dec-9-enyl-3-hydroxy-5-oxooxolane-2,3-dicarboxylic acid | Cid_23872057 |
|  | 2-(6-Hydroxyhexyl)-3-methylenesuccinic acid | Cid_23872059 |
|  | 2-Glucosyloxy-4-methoxycinnamic acid | Cid_23872107 |
|  | ACon1_000569 | Cid_23874473 |
|  | Eupassopilin | Cid_23900111 |
|  | 4-(2,7-Dihydroxy-6-methylheptan-2-yl)-3-hydroxybenzoic acid | Cid_23928030 |
|  | 5-(Hydroxymethyl)-3-(1-hydroxy-4-methylhexyl)oxolan-2-one | Cid_23930375 |
|  | 5-O-Methylsulochrin | Cid_23983701 |
|  | 2,4-Tetradecadienedioic acid, 12-hydroxy-13-(hydroxymethyl)-3,5,7-trimethyl-, 14-methyl ester | Cid_23983705 |
|  | 5-Methyl-1-(2-methylpropyl)pyrimidine-2,4(1H,3H)-dione | Cid_240106 |
|  | Sydonol | Cid_24011597 |
|  | (6E,10Z,14E)-2-Keto-6,14-dimethyl-3-methylene-3a,4,5,8,9,12,13,15a-octahydrocyclotetradeca[b]furan-10-carboxylic acid | Cid_24011680 |
|  | 1,7-Bis(3,4-dihydroxyphenyl)heptan-3-yl acetate | Cid_24013835 |
|  | Tuberonic acid glucoside | Cid_24066896 |
|  | 15-Octadecenoic acid, 9,12,13-trihydroxy- | Cid_24066906 |
|  | Rupestonic acid | Cid_24094149 |
|  | (Z)-2-.beta.-D-Glucopyranosyloxy-4-methoxycinnamic acid | Cid_24094157 |
|  | 9-Octadecenoic acid, 5,8,11-trihydroxy- | Cid_24096399 |
|  | 9-Octadecenoic acid, 5,8,11-trihydroxy- | Cid_24096399 |
|  | 2-Furanacetic acid, 5-(2,12-dihydroxy-3,6-dodecadien-1-yl)tetrahydro- | Cid_24150636 |
|  | 1,3-Bis(2-benzimidazolyl)propane | Cid_242973 |
|  | 3-Hydroxy-5-methoxy-2,2-dimethyl-7-phenethylchroman-8-carboxylic acid | Cid_24739240 |
|  | CAY10581 | Cid_24824610 |
|  | Iclusig | Cid_24826799 |
|  | 4-Hydroxy-5-(4-hydroxy-1-methylbutyl)-6-methyl-3-methylene-3a,4,7,7a-tetrahydrobenzofuran-2-one | Cid_25018670 |
|  | Aureonitol | Cid_25064137 |
|  | 9,10-DiHOME | Cid_25320858 |
|  | 9,10-DiHOME | Cid_25320858 |
|  | 12,13-DiHOME | Cid_25320868 |
|  | 2',3'-cAMP | Cid_25796534 |
|  | Ononetin | Cid_259632 |
|  | Cedrin | Cid_26087902 |
|  | 8-Hydroxyageraphorone | Cid_26297667 |
|  | 3-Hydroxycaprylic acid | Cid_26613 |
|  | 4-Methyl-2-oxo-1(2H)-quinolineacetic acid | Cid_268297 |
|  | 1,5-Pentanediamine | Cid_273 |
|  | 2,3-Dihydro-3-oxo-4H-1,4-benzoxazine-4-acetic acid | Cid_2735705 |
|  | L-Canavanine | Cid_275 |
|  | L-.beta.-Homolysine | Cid_2761529 |
|  | 4-(5-Oxazolyl)benzoic acid | Cid_2776414 |
|  | Benzotript | Cid_2787526 |
|  | 3-(Methylsulfonamido)benzaldehyde | Cid_2794787 |
|  | PyroGlu-Val | Cid_28191825 |
|  | 2,4-Dihydroxy-6-pentylbenzoate | Cid_2826719 |
|  | 4-(4-Hydroxyphenyl)-6-methyl-3-oxabicyclo[3.3.1]non-6-ene-1-methanol | Cid_2828563 |
|  | 9-Nitro-3a,4,5,9b-tetrahydro-3H-cyclopenta[c]quinoline-4,6-dicarboxylic acid | Cid_2872935 |
|  | N-[2-(5-Methoxy-1H-indol-3-yl)ethyl]cyclopropanecarboxamide | Cid_2877 |
|  | 1-(2,5-Dimethylphenoxy)-3-morpholinopropan-2-ol | Cid_2882685 |
|  | Pyrocatechol | Cid_289 |
|  | N-Methylanabasine | Cid_29758 |
|  | Gabapentin related compound D | Cid_29980616 |
|  | Gly-Pro | Cid_3013625 |
|  | 3-Hydroxysebacic acid | Cid_3017884 |
|  | N-(1-Naphthyl)-N'-(2-pyridinyl)urea | Cid_302856 |
|  | Dicamba | Cid_3030 |
|  | D-Glucosamine | Cid_3034366 |
|  | 3-Pyrrolidinecarboxylic acid | Cid_3034645 |
|  | NAD 299 | Cid_3055171 |
|  | Fallacinol | Cid_3083633 |
|  | Dipivefrine | Cid_3105 |
|  | Diprotin A | Cid_3107 |
|  | Citric acid | Cid_311 |
|  | Citric acid | Cid_311 |
|  | 1-Naphthyl-N-(tetrahydro-2-furanylmethyl)methanamine | Cid_3156809 |
|  | (.+/-.)-Dropropizine | Cid_3169 |
|  | PRIMA-1 | Cid_322968 |
|  | Methyl morpholine-3-carboxylate | Cid_3286806 |
|  | Ncgc00385871-01_c15h22o4_2,8-dihydroxy-5,5,8-trimethyl-11-oxatetracyclo[7.3.1.0~1,9~.0~3,7~]tridecan-10-one | Cid_329432 |
|  | Syringaresinol | Cid_332426 |
|  | Indole-4-carboxaldehyde | Cid_333703 |
|  | (-)-Medicarpin | Cid_336327 |
|  | Salicylic acid | Cid_338 |
|  | Psicose | Cid_3426 |
|  | 1-{[4-(Dimethylamino)benzyl]amino}-2-propanol | Cid_344187 |
|  | 1-[(Dimethylamino)carbonyl]-3-piperidinecarboxylic acid | Cid_3470698 |
|  | Sakuranetin | Cid_348130 |
|  | Butin | Cid_3496769 |
|  | Val-Leu | Cid_352039 |
|  | Val-Leu | Cid_352039 |
|  | 3'-Hydroxyflavanone | Cid_3534982 |
|  | N-Ethyl-4-(dimethylamino)benzylamine | Cid_3542461 |
|  | Ferutinin | Cid_354654 |
|  | [3-Me-His2] TRH | Cid_3548445 |
|  | 4-Methylcyclohexaneacetic acid | Cid_3616945 |
|  | Isopropalin | Cid_36606 |
|  | Cyclo(prolyltyrosyl) | Cid_371682 |
|  | Ferocaulidin | Cid_3725594 |
|  | 2-Hydroxy-2-(2-keto-2-methoxyethyl)succinic acid | Cid_378532 |
|  | 6-Piperazino-7H-purine | Cid_3810979 |
|  | Glucoside, 3-hydroxy-5-(p-hydroxyphenethyl)phenyl | Cid_38348878 |
|  | Hydroheptelidic acid | Cid_38354714 |
|  | Hydroheptelidic acid | Cid_38354714 |
|  | 2-((1S,2S,4aR,8aS)-1-hydroxy-4a-methyl-8-methylenedecahydronaphthalen-2-yl)acrylic acid | Cid_38357975 |
|  | (E)-1,7-Bis(4-hydroxyphenyl)hept-1-en-3-one | Cid_38361084 |
|  | Pimelic acid | Cid_385 |
|  | 7-Acetonyloxy-6-methyl-2,3-dihydro-1H-cyclopenta[c]chromen-4-one | Cid_3852139 |
|  | Provitamin B3 | Cid_3856251 |
|  | Lenticine | Cid_3861164 |
|  | (2R)-6-Ketopipecolinic acid | Cid_40424009 |
|  | Val-Tyr | Cid_40492428 |
|  | Val-Glu | Cid_40520338 |
|  | Virginiaebutanolide C | Cid_40565489 |
|  | (.+/-.)-Mephenesin | Cid_4059 |
|  | Trolox | Cid_40634 |
|  | rac-4-(Methylamino)-1-(3-pyridyl)-1-butanol | Cid_4071159 |
|  | 4-(2-Phenoxyethoxy)aniline | Cid_4177311 |
|  | 6-(Hydroxymethyl)pyridin-3-ol | Cid_419490 |
|  | 5-[(4-Bromo-2-chlorophenoxy)methyl]furfural | Cid_4250163 |
|  | 6-(4-hydroxy-6-methoxy-7-methyl-3-oxo-1H-isobenzofuran-5-yl)-4-methyl-4-hexenoic acid | Cid_4272 |
|  | 2-(Dimethylamino)-N-(1,5-dimethyl-3-oxo-2-phenyl-2,3-dihydro-1H-pyrazol-4-yl)acetamide | Cid_42905 |
|  | N-(4-Methylpentanoyl)phenylalanine | Cid_43183355 |
|  | 4-Ethyl-N,N-dimethylcathinone | Cid_43794772 |
|  | 5'-S-Methyl-5'-thioadenosine | Cid_439176 |
|  | Guanosine 5'-diphospho-.beta.-L-fucose | Cid_439211 |
|  | L-Carnosine | Cid_439224 |
|  | L-Homoproline | Cid_439227 |
|  | L-Ornithine, N2-acetyl- | Cid_439232 |
|  | Naringenin | Cid_439246 |
|  | sn-Glycero-3-phosphocholine | Cid_439285 |
|  | beta-D-Galactose | Cid_439353 |
|  | Glycine, N-(aminocarbonyl)-N-methyl- | Cid_439375 |
|  | D-Asparagine | Cid_439600 |
|  | D-Pyroglutamic acid | Cid_439685 |
|  | N-Acetyl-D-tryptophan | Cid_439917 |
|  | L-2-Hydroxyglutaric acid | Cid_439939 |
|  | N6,N6,N6-Trimethyl-L-lysine | Cid_440120 |
|  | N,N-Dimethylhistidine | Cid_440274 |
|  | 8-Deoxylactucin | Cid_442196 |
|  | (-)-Salsoline | Cid_442356 |
|  | 7,8-dihydroxy-6-methoxy-3-(4-methoxyphenyl)-4H-chromen-4-one | Cid_44257265 |
|  | [6]-Gingerol | Cid_442793 |
|  | Ononin | Cid_442813 |
|  | Ononin | Cid_442813 |
|  | 1-[4-Hydroxy-3-(3-methylbut-2-enyl)phenyl]ethanone | Cid_442916 |
|  | Ile-Thr | Cid_44298493 |
|  | (-)-Syringaresinol-4-O-.beta.-D-glucopyranoside | Cid_443024 |
|  | (-)-Syringaresinol-4-O-.beta.-D-glucopyranoside | Cid_443024 |
|  | Vinpocetine | Cid_443955 |
|  | Pro-Leu | Cid_444109 |
|  | Maleic acid | Cid_444266 |
|  | cis-ACCP | Cid_44457233 |
|  | L-(-)-3-Phenyllactic acid | Cid_444718 |
|  | (+)-2-Phenyllactic acid | Cid_445144 |
|  | MMV023233 | Cid_44525694 |
|  | TCMDC-138924 | Cid_44533020 |
|  | Ferulic acid | Cid_445858 |
|  | 3,5-Dihydroxycapric acid | Cid_44715204 |
|  | 3',4'-Dihydroinfectopyrone | Cid_44715238 |
|  | 3',4'-Dihydroinfectopyrone | Cid_44715238 |
|  | 6-Hydroxy-3,8a-dimethyl-5-methylene-4a,6,7,8,9,9a-hexahydro-4H-benzo[f]benzofuran-2-one | Cid_44715317 |
|  | 6-[(E)-But-2-en-2-yl]-4-[6-[(Z)-but-2-en-2-yl]-2,4-dihydroxy-3-methylbenzoyl]oxy-2-hydroxy-3-methylbenzoic acid | Cid_44715333 |
|  | Pentalenolactone D | Cid_44715352 |
|  | (E)-2-Decylpent-2-enedioic acid | Cid_44715396 |
|  | MCULE-7407861812 | Cid_44715452 |
|  | MCULE-7407861812 | Cid_44715452 |
|  | 1-[4-[2-(7-Methoxy-1,3-benzodioxol-5-yl)ethyl]phenoxy]-3-methylbutane-2,3-diol | Cid_44715781 |
|  | Adenosine | Cid_447270 |
|  | 1-Acetylpipecolinic acid | Cid_4472712 |
|  | 8-Amino-7-oxononanoic acid | Cid_448124 |
|  | 3,4-Dimethyl-2,5-bis(3,4,5-trimethoxyphenyl)oxolane | Cid_4485701 |
|  | Ile-Val | Cid_449407 |
|  | Avermitilol | Cid_45036058 |
|  | L-.gamma.-Glutamyl-L-leucine | Cid_4524287 |
|  | 7-Oxabicyclo[4.1.0]hept-3-en-2-one, 5-hydroxy-3-(hydroxymethyl)-1-(1-hydroxy-3-methyl-2-buten-1-yl)-4-(1-propen-1-yl)- | Cid_45359217 |
|  | 3-(1,2-Dihydroxypropyl)-1,6,8-trihydroxy-9,10-anthraquinone | Cid_45359290 |
|  | 3-Heptyl-3,6-dihydro-1H-furo[3,4-c]furan-4-one | Cid_45359295 |
|  | 9-Hydroxy-9-[[(E)-2-(hydroxymethyl)-3-[3-(hydroxymethyl)-6-propan-2-ylcyclohex-2-en-1-yl]prop-2-enoyl]oxymethyl]-1-oxo-6-propan-2-yl-3,5a,6,7,8,9a-hexahydro-2-benzoxepine-4-carboxylic acid | Cid_45359306 |
|  | 4-(5,6-Dihydroxyheptyl)-3-methylfuran-2(5H)-one | Cid_45359400 |
|  | 7-Hydroxy-9,10,14-trimethoxy-2-methyl-3,4,5,6-tetrahydro-2H,8H-2,6-epoxyoxocino[3,2-b]xanthen-8-one | Cid_45359438 |
|  | 3,8,9-Trihydroxy-10-propyl-3,4,5,8,9,10-hexahydro-2H-oxecin-2-one | Cid_45359453 |
|  | [4,5-Dihydroxy-3,4-bis(hydroxymethyl)-4a,8,8-trimethyl-5,6,7,8a-tetrahydro-1H-naphthalen-1-yl] hexanoate | Cid_45359477 |
|  | 4-(Hydroxymethyl)-3,4a,8,8-tetramethyl-1,2,5,6,7,8a-hexahydronaphthalene-1,2-diol | Cid_45359517 |
|  | 1-O-(3-Hydroxy-4,5-dimethoxybenzoyl)hexopyranose | Cid_45359580 |
|  | Aromadendrin 6-C-glucoside | Cid_45359599 |
|  | 3',5a'-Dimethyl-2'-oxodecahydro-2'H-spiro[oxirane-2,8'-oxireno[6,7]naphtho[1,2-b]furan]-6'-yl (2Z)-2-methylbut-2-enoate | Cid_45359674 |
|  | 3-((3aS,6S,8aR)-8-Hydroxy-6,8-dimethyl-3-methylidene-2-oxooctahydro-2H-cyclohepta[b]furan-7-yl)propanoic acid | Cid_45359685 |
|  | 2,2a,6,6a,7a,8,9,9a-Octahydro-1a,5,7a-trimethylbisoxireno[4,5:8,9]cyclodeca[1,2-b]furan-4(1aH)-one | Cid_45359717 |
|  | 4,11-Dimethyl-8-(propan-2-yl)-5,12-dioxatricyclo[9.1.0.04,6]dodecan-7-ol | Cid_45359817 |
|  | Garbanzol | Cid_45360091 |
|  | 3-(5-(3,4-Dihydroxy-4-methyl-5-oxotetrahydrofuran-2-yl)-2-((6-O-(3-(5-(3,4-dihydroxy-4-methyl-5-oxotetrahydrofuran-2-yl)-2-(.beta.-D-glucopyranosyloxy)-4-methoxyphenyl)propanoyl)-.beta.-D-glucopyranosyl)oxy)-4-methoxyphenyl)propanoic acid | Cid_45360165 |
|  | 2-(2-Hydroxy-5-((1E)-3-hydroxyprop-1-en-1-yl)-3-methoxyphenyl)-3-(4-hydroxy-3-methoxyphenyl)propyl hexopyranoside | Cid_45360204 |
|  | 4-[3-(3,4-Dihydroxyphenyl)acryloyloxy]-2,3-dihydroxy-2-methylbutyric acid | Cid_45360267 |
|  | 6,10a-Dihydroxy-4-(hydroxymethyl)-4,7,11b-trimethyl-1,2,3,4a,5,6,6a,7,11,11a-decahydronaphtho[2,1-f][1]benzofuran-9-one | Cid_45360321 |
|  | Undatuside A | Cid_45360328 |
|  | Norfloxacin | Cid_4539 |
|  | Homopterocarpin | Cid_454892 |
|  | Methyl 4-((4,6-dihydroxy-5-methoxy-2,5-dimethyl-3-oxocyclohex-1-en-1-yl)oxy)-2-hydroxy-3,6-dimethylbenzoate | Cid_45782752 |
|  | (E)-2-(Hydroxymethyl)-3-(3-oxo-5-propan-2-yl-4,5,6,7-tetrahydro-1H-2-benzofuran-4-yl)prop-2-enoic acid | Cid_45782757 |
|  | (3-(2,4'-Dihydroxy-3,6-dimethoxy[biphenyl]-4-yl)-5-oxo-2,5-dihydrofuran-2-yl)acetic acid | Cid_45782803 |
|  | 1-Hydroxy-1-(hydroxymethyl)-8,8-dimethyl-2-oxo-4a,6a,7,9-tetrahydro-4H-pentaleno[1,6a-c]pyran-5-carboxylic acid | Cid_45782878 |
|  | 1,3,4-Trihydroxy-5-(((2E)-3-(2-(4-hydroxy-3-methoxyphenyl)-3-(hydroxymethyl)-7-methoxy-2,3-dihydro-1-benzofuran-5-yl)prop-2-enoyl)oxy)cyclohexanecarboxylic acid | Cid_45782923 |
|  | 3-Hydroxy-3,5,5-trimethyl-4-(3-oxobut-1-en-1-ylidene)cyclohexyl .beta.-D-glucopyranoside | Cid_45783010 |
|  | 8-O-4-Hydroxycinnamoylharpagide | Cid_45783053 |
|  | 3-(Benzoyloxy)-2-hydroxypropyl .beta.-D-glucopyranosiduronic acid | Cid_45783079 |
|  | 1,9b-Dihydroxy-6,6,9a-trimethyl-1,5,5a,7,8,9-hexahydrobenz[e]isobenzofuran-3-one | Cid_45783128 |
|  | 9-Glyceryloxy-9-ketopelargonic acid | Cid_45783154 |
|  | 9-Glyceryloxy-9-ketopelargonic acid | Cid_45783154 |
|  | Methyl 2-(4-ethenyl-2,6-dihydroxy-3-(3-hydroxyprop-1-en-2-yl)-4-methylcyclohexyl)prop-2-enoate | Cid_45783164 |
|  | 5,6-Dimethoxy-3-(4-methoxyphenyl)-4-oxo-4H-chromen-7-yl 6-deoxy-.alpha.-L-mannopyranosyl-(1->6)-[6-deoxy-.alpha.-L-mannopyranosyl-(1->6)-.beta.-D-glucopyranosyl-(1->4)]-.beta.-D-glucopyranoside | Cid_45783184 |
|  | (2E,4E)-5-(6-((.beta.-D-Glucopyranosyloxy)methyl)-1-hydroxy-2,6-dimethyl-4-oxocyclohex-2-en-1-yl)-3-methylpenta-2,4-dienoic acid | Cid_45783222 |
|  | (9Z,12E)-15,16-Dihydroxyoctadeca-9,12-dienoic acid | Cid_45783236 |
|  | (9Z,12E)-15,16-Dihydroxyoctadeca-9,12-dienoic acid | Cid_45783236 |
|  | 2'-Hydroxy-2,4,5,6'-tetramethoxychalcone | Cid_45933916 |
|  | Salicylic acid beta-D-glucoside | Cid_4596190 |
|  | Oxymetazoline | Cid_4636 |
|  | (3aR,4aR,5R,7S,7aS)-5-Hydroxy-5,8-dimethyl-3-methylene-2-oxo-2,3,3a,4,4a,5,6,7,7a,9a-decahydroazuleno[6,5-b]furan-7-yl acetate | Cid_46397418 |
|  | Hippuric acid | Cid_464 |
|  | 7-(1-Hydroxy-1-methylethyl)-1,4a-dimethyldecalin-1-ol | Cid_4655876 |
|  | Provitamin B | Cid_4678 |
|  | 5-{[2-(4-Chlorophenyl)ethyl]amino}-5-oxopentanoic acid | Cid_4685096 |
|  | Glyasperin D | Cid_480860 |
|  | Pindolol | Cid_4828 |
|  | Vitamin B2 | Cid_493570 |
|  | 2-(8-Hydroxy-4a,8-dimethyldecalin-2-yl)acrylic acid | Cid_496073 |
|  | 2-(8-Hydroxy-4a,8-dimethyldecalin-2-yl)acrylic acid | Cid_496073 |
|  | Benedorm | Cid_4994 |
|  | 4-Guanidinobutyric acid | Cid_500 |
|  | Methyl 2,4,6-tri-tert-butylbenzoate | Cid_5059268 |
|  | (4E,6E)-3-(3-Keto-3-methoxypropyl)-2-methyloldeca-4,6-dienoic acid | Cid_51136263 |
|  | 1-[2-Hydroxy-4-(3-hydroxy-5-methylphenoxy)-6-methylphenyl]-3-methylbutane-2,3-diol | Cid_51136316 |
|  | Gallomyrtucommulone C | Cid_51136364 |
|  | Eurycomalactone | Cid_51136366 |
|  | 9.xi.-O-.beta.-D-Glucopyranosyloxy-5-megastigmen-4-one | Cid_51136393 |
|  | 4-[2-[(2-Ethyl-2,3-dihydroxybutanoyl)oxymethyl]anilino]-4-ketobutyric acid | Cid_51136455 |
|  | 3-C-(((2-(3-Carboxypropanamido)benzyl)oxy)carbonyl)-1,5-dideoxypentitol | Cid_51136523 |
|  | 4,4,7a-Trimethyl-3a,5,6,7-tetrahydro-3H-indene-1-carboxylic acid | Cid_51136570 |
|  | Grandidentoside | Cid_51136585 |
|  | 4-(2,6,6-Trimethyl-4-oxocyclohex-2-en-1-yl)butan-2-yl 6-O-(3,4,5-trihydroxybenzoyl)hexopyranoside | Cid_51136595 |
|  | Sebacic acid | Cid_5192 |
|  | 4-Formyl-3-methoxyphenol | Cid_519541 |
|  | N-Malonyltryptophan | Cid_5199636 |
|  | DL-Prolylphenylalanine | Cid_5226097 |
|  | DL-Malic acid | Cid_525 |
|  | N,N'-Bis(4-ethoxyphenyl)formamidine | Cid_525811 |
|  | Neopetasol | Cid_5275908 |
|  | Biochanin A | Cid_5280373 |
|  | Biochanin A | Cid_5280373 |
|  | Formononetin | Cid_5280378 |
|  | Formononetin | Cid_5280378 |
|  | Calycosin | Cid_5280448 |
|  | Calycosin | Cid_5280448 |
|  | Maleamate | Cid_5280451 |
|  | trans-Glutaconic acid | Cid_5280498 |
|  | Sinapyl alcohol | Cid_5280507 |
|  | Sissotrin | Cid_5280781 |
|  | Sissotrin | Cid_5280781 |
|  | trans-3,5-Dimethoxy-4-hydroxycinnamaldehyde | Cid_5280802 |
|  | Isokaempferide | Cid_5280862 |
|  | Genistein | Cid_5280961 |
|  | Genistein | Cid_5280961 |
|  | Jasmonic acid | Cid_5281166 |
|  | Corylifolinin | Cid_5281255 |
|  | Sulfuretin | Cid_5281295 |
|  | Ophiobolin A | Cid_5281387 |
|  | Zearalenone | Cid_5281576 |
|  | Dinatin | Cid_5281628 |
|  | Macluraxanthone | Cid_5281646 |
|  | Wogonin | Cid_5281703 |
|  | Coumestrol | Cid_5281707 |
|  | Coumestrol | Cid_5281707 |
|  | Pterostilbene | Cid_5281727 |
|  | Prunetin | Cid_5281804 |
|  | (E)-.alpha.-Ionone | Cid_5282108 |
|  | trans-EKODE-(E)-Ib | Cid_5283007 |
|  | 9-Oxo-10(E),12(E)-octadecadienoic acid | Cid_5283011 |
|  | trans-Traumatic acid | Cid_5283028 |
|  | trans-Traumatic acid | Cid_5283028 |
|  | .gamma.-Linolenoyl ethanolamide | Cid_5283445 |
|  | 3-(2-Nitro-1-propenyl)indole | Cid_5291187 |
|  | 1,1 Dimethyl 4 Phenylpiperazinium | Cid_5310977 |
|  | EGLU | Cid_5311079 |
|  | Corylin | Cid_5316097 |
|  | Dianthoside | Cid_5316639 |
|  | 6,7-Dihydroxyligustilide | Cid_5316704 |
|  | Calycosin-7-o-beta-d-glucoside | Cid_5318267 |
|  | Calycosin-7-o-beta-d-glucoside | Cid_5318267 |
|  | Ligustilide A | Cid_5319022 |
|  | Methyl 4-coumarate | Cid_5319562 |
|  | Hortensin | Cid_5320438 |
|  | Psilostachyin B | Cid_5320768 |
|  | Sophoricoside | Cid_5321398 |
|  | Lumichrome | Cid_5326566 |
|  | Orantinib | Cid_5329099 |
|  | Thioarginine | Cid_53394341 |
|  | Uralenneoside | Cid_53462251 |
|  | 2-Ethylidene-1,5-dimethyl-3,3-diphenylpyrrolidine | Cid_5352621 |
|  | Zerumbone | Cid_5353004 |
|  | .beta.-Damascone | Cid_5374527 |
|  | 3-Indoleacrylic acid | Cid_5375048 |
|  | trans-Abscisin | Cid_5375188 |
|  | (+)-Abscisic acid | Cid_5375199 |
|  | 4,4'-Dimethoxychalcone | Cid_5377817 |
|  | 3,2'-Dihydroxy-4,4',6'-trimethoxychalcone | Cid_5379071 |
|  | 3,2'-Dihydroxy-4,4',6'-trimethoxychalcone | Cid_5379071 |
|  | 2'-Hydroxy-4'-methoxychalcone | Cid_5380645 |
|  | Pro-Thr | Cid_53860028 |
|  | Helenin | Cid_5386173 |
|  | 7,3'-Dihydroxyflavone | Cid_5391140 |
|  | 7,2'-Dihydroxyflavone | Cid_5391149 |
|  | 7-Hydroxy-3'-methoxyflavone | Cid_5393153 |
|  | 7-Methyl-3-methylidene-6-(3-oxobutyl)-4,7,8,8a-tetrahydro-3aH-cyclohepta[b]furan-2-one | Cid_540288 |
|  | Cys-Val | Cid_54146020 |
|  | Gln-Met | Cid_54307034 |
|  | .gamma.-Mangostin | Cid_5464078 |
|  | 4-Hydroxy-3-[1-(5-hydroxy-2,6,6-trimethyl-tetrahydropyran-2-yl)ethyl]carbostyril | Cid_54680116 |
|  | Antibiotic TAN 1446A | Cid_54742784 |
|  | Tuberonic acid | Cid_5497122 |
|  | H-gamma-glu-phe-oh | Cid_558649 |
|  | N-Phenylacetylaspartic acid | Cid_562665 |
|  | 9,10,11-Trihydroxy-12(Z),15(Z)-octadecadienoic acid | Cid_56671117 |
|  | MCULE-1175390561 | Cid_56773937 |
|  | Arillatose B | Cid_56776292 |
|  | Longipin | Cid_56776401 |
|  | YC-1 | Cid_5712 |
|  | Gln-Val | Cid_57262456 |
|  | 4-Acetoxy-8-(3-keto-2-pent-2-enylcyclopenten-1-yl)caprylic acid | Cid_57481829 |
|  | 4-Methyl-.alpha.-pyrrolidinobutiophenone | Cid_57486975 |
|  | 2-(6-Hydroxy-2-keto-3,8-dimethyl-4,5,6,7,8,8a-hexahydro-1H-azulen-5-yl)acrylic acid | Cid_57509376 |
|  | 7-Hydroxy-9-(hydroxymethyl)-1,4,9-trimethyl-2,4,5,7,8,8a-hexahydro-4,7-methanoazulen-6(1H)-one | Cid_57509414 |
|  | Methyl 6-(acetyloxy)-1,3,7,11-tetrahydroxyabieta-8,11,13-trien-18-oate | Cid_57509420 |
|  | 6-(3-(.beta.-D-Glucopyranosyloxy)butyl)-5,5-dimethyl-3-oxocyclohex-1-ene-1-carboxylic acid | Cid_57509445 |
|  | 5,8-Dihydroxy-1,5,8-trimethyl-4,5a,6,7,8a,9-hexahydro-3aH-azuleno[6,5-b]furan-2-one | Cid_57509469 |
|  | 2-Dec-9-enylglutaric acid | Cid_57509489 |
|  | .beta.-Estradiol | Cid_5757 |
|  | D-Glucitol | Cid_5780 |
|  | 7-hydroxy-2',4'-dimethoxyisoflavone | Cid_5781145 |
|  | 2,3-Divanillyl-1,4-butanediol | Cid_586372 |
|  | (E)-5-((1S,4R)-3,3-Dimethylbicyclo[2.2.1]heptan-2-yl)pent-3-en-2-one | Cid_5937532 |
|  | 6-Methylchromone | Cid_594810 |
|  | Cytosine | Cid_597 |
|  | 3-Methoxy-4,2',5'-trihydroxychalcone | Cid_5976625 |
|  | Levulose | Cid_5984 |
|  | 4-Hydroxy-2',4',6'-trimethoxychalcone | Cid_5986673 |
|  | Sucrose | Cid_5988 |
|  | Sucrose | Cid_5988 |
|  | 3-O-Acetyl-13-deoxyphomenone | Cid_60208854 |
|  | 4-((1S,4R)-4-(4-Hydroxy-3,5-dimethoxyphenyl)tetrahydro-1H,3H-furo[3,4-c]furan-1-yl)-2,6-dimethoxyphenyl 2-O-((2S,3R,4R)-3,4-dihydroxy-4-(hydroxymethyl)tetrahydrofuran-2-yl)-.beta.-D-glucopyranoside | Cid_60208860 |
|  | Spirodionic acid | Cid_60208865 |
|  | Spirodionic acid | Cid_60208865 |
|  | Antibiotic JBIR 27 | Cid_60208893 |
|  | Lasiodiplodin | Cid_602765 |
|  | Uridine | Cid_6029 |
|  | Lychnophoic acid | Cid_6049852 |
|  | 4,9-Dihydroxy-6-methyl-3,10-dimethylene-4,7,8,9,11,11a-hexahydro-3aH-cyclodeca[b]furan-2-one | Cid_6110227 |
|  | Androstenedione | Cid_6128 |
|  | Ala-Ile | Cid_61578913 |
|  | Cytidine | Cid_6175 |
|  | N-Acetyl-L-methionine | Cid_6180 |
|  | Cycloheximide | Cid_6197 |
|  | 2,5'-Dimethoxy-2'-hydroxychalcone | Cid_6211950 |
|  | His | Cid_6274 |
|  | 4-(2,4,6-Trimethyl-3-cyclohexen-1-yl)-3-buten-2-one | Cid_6285057 |
|  | L-Tryptophan | Cid_6305 |
|  | Arginine | Cid_6322 |
|  | Arginine | Cid_6322 |
|  | Pro-Phe | Cid_6351946 |
|  | trans-3-Coumaric acid | Cid_637541 |
|  | p-Coumaric acid | Cid_637542 |
|  | Sinapic acid | Cid_637775 |
|  | Sinapic acid | Cid_637775 |
|  | Isoliquiritigen | Cid_638278 |
|  | 3,4'-Dihydroxypropiophenone | Cid_638759 |
|  | 4-(Cyclohexylcarbamoylamino)butyric acid | Cid_6420119 |
|  | .beta.-Ala-Phe | Cid_6426945 |
|  | Ser-Leu | Cid_6427005 |
|  | Cyclo(isoleucylprolyl) | Cid_6428988 |
|  | 2-Methoxy-3,5-dimethylpyrazine | Cid_6429218 |
|  | cis-Aconitic acid | Cid_643757 |
|  | Citraconic acid | Cid_643798 |
|  | Valerenic acid | Cid_6440940 |
|  | (-)-Dihydrojasmonic acid | Cid_644120 |
|  | Mycophenolic acid .beta.-D-glucuronide | Cid_6442661 |
|  | Echinatin | Cid_6442675 |
|  | N-Lauroyl-L-arginine | Cid_6451888 |
|  | trans-C 75 | Cid_6482234 |
|  | trans-C 75 | Cid_6482234 |
|  | Arctigenin | Cid_64981 |
|  | N-Acetyl-L-aspartic acid | Cid_65065 |
|  | Isopropylfumaric acid | Cid_6508102 |
|  | (-)-Secoisolariciresinol | Cid_65373 |
|  | Valerenolic acid | Cid_6537505 |
|  | Val-Met | Cid_65549302 |
|  | 4-Formyl-2-hydroxybenzoic acid | Cid_656876 |
|  | Glycerophosphocholine | Cid_657272 |
|  | Pantothenate | Cid_6613 |
|  | Piceatannol | Cid_667639 |
|  | 4-Pyridoxic acid | Cid_6723 |
|  | 4-O-Methylpinosylvic acid | Cid_67322417 |
|  | 2-(4-tert-Butylbenzoyl)benzoic acid | Cid_67357 |
|  | L-Arginine, N2-acetyl- | Cid_67427 |
|  | 5-Nitro-N-(2-phenylethyl)-2-furamide | Cid_676060 |
|  | 3,2'-Dimethoxyflavone | Cid_676169 |
|  | Orsellinate | Cid_68072 |
|  | Isoimperatorin | Cid_68081 |
|  | Benzoylmalic acid | Cid_68152266 |
|  | N-Acetyl-L-tyrosine | Cid_68310 |
|  | 6-Hydroxy-4'-methoxyflavone | Cid_688679 |
|  | 2'-Methoxy-7,8-benzoflavone | Cid_688681 |
|  | 4'-Methoxy-6-methylflavone | Cid_688682 |
|  | 3,6,3',4'-Tetramethoxyflavone | Cid_688812 |
|  | N-(4-Phenoxyphenyl)-2-pyrazinecarboxamide | Cid_695409 |
|  | Leu-Ser | Cid_6992130 |
|  | Leu-Ser | Cid_6992130 |
|  | Phe-Gly | Cid_6992304 |
|  | Phe-Gly | Cid_6992304 |
|  | Glu-Val | Cid_6992567 |
|  | Val-Ala | Cid_6992638 |
|  | Val-Gly | Cid_6993111 |
|  | Leu-Val | Cid_6993116 |
|  | N-.delta.-BOC-L-Ornithine | Cid_6993436 |
|  | N-Acetyl-D-norleucine | Cid_6995106 |
|  | Lys-Ile | Cid_7010499 |
|  | Thr-Gly | Cid_7010575 |
|  | Lys-Thr | Cid_7010718 |
|  | Phe-Pro | Cid_7020642 |
|  | Isoephedrine | Cid_7028 |
|  | L,L-Cyclo(leucylprolyl) | Cid_7074739 |
|  | Pro-Asp | Cid_7079450 |
|  | Pro-Ile | Cid_7079601 |
|  | N-Acetyl-L-leucine | Cid_70912 |
|  | N-Acetyl-L-glutamic acid | Cid_70914 |
|  | Hepasil | Cid_71170 |
|  | Andrographolide | Cid_71308151 |
|  | L-Glutarylcarnitine | Cid_71317118 |
|  | Dianisalacetone | Cid_715840 |
|  | 2-Pentenedioic acid, 2-octyl- | Cid_71694435 |
|  | Zederone | Cid_71694446 |
|  | 7-((3-O-.beta.-D-Glucopyranosyl-2-O-((2E)-3-(4-hydroxyphenyl)prop-2-enoyl)-.beta.-D-glucopyranosyl)oxy)-5-hydroxy-2-(4-hydroxyphenyl)-4-oxo-4H-chromen-3-yl 2-O-(6-deoxy-.alpha.-L-mannopyranosyl)-.beta.-D-glucopyranoside | Cid_71694456 |
|  | Curcuminol G | Cid_71694468 |
|  | Curcuminol G | Cid_71694468 |
|  | Propofol glucuronide | Cid_71751823 |
|  | 16.alpha.,17,21-Trihydroxypregna-1,4-diene-3,11,20-trione | Cid_71752789 |
|  | N-Acetyl-D-proline | Cid_719436 |
|  | N-Acetyl-D-proline | Cid_719436 |
|  | Polygodial | Cid_72503 |
|  | Parthenolide | Cid_7251185 |
|  | Coumarin 334 | Cid_72655 |
|  | FA 18:2+3o | Cid_72732143 |
|  | 2-(1H-Pyrrol-1-yl)benzohydrazide | Cid_727889 |
|  | 1-O-Feruloylglucose | Cid_72983454 |
|  | 3-Aminoglutaric acid | Cid_73064 |
|  | (.+/-.)-Medicarpin | Cid_73067 |
|  | Lactobionic acid | Cid_7314 |
|  | Epiligulyl oxide | Cid_73174 |
|  | 6-(2-Hydroxypropan-2-yl)-4,8a-dimethyl-2,3,4,6,7,8-hexahydro-1H-naphthalen-1-ol | Cid_73189537 |
|  | BES | Cid_73243 |
|  | Pinoresinol | Cid_73399 |
|  | 2-Methoxycinnamic acid | Cid_734154 |
|  | Tetrahydroharman-3-carboxylic acid | Cid_73530 |
|  | Phenethylthiourea | Cid_735845 |
|  | Methyl (2E)-3-(3,4,5-trimethoxyphenyl)prop-2-enoate | Cid_735846 |
|  | Pidolic acid | Cid_7405 |
|  | Pro-Asn | Cid_7408192 |
|  | Citrazinic acid | Cid_7425 |
|  | Botran | Cid_7430 |
|  | N-Acetyl-L-phenylalanine | Cid_74839 |
|  | 1-(1,2-Dihydro-6-hydroxy-5-acenaphthylenyl)ethanone | Cid_749958 |
|  | Mmv676588 | Cid_751739 |
|  | N-Phenylglycine ethyl ester | Cid_75190 |
|  | Berkedrimane A | Cid_75368727 |
|  | 8-Methoxyatractylenolide I | Cid_75368731 |
|  | Methyl 3-[4-hydroxy-2-(2-methylpropyl)-1-oxo-3,3a-dihydro-2H-imidazo[1,2-a]indol-4-yl]-2-(4-oxoquinazolin-3-yl)propanoate | Cid_75368782 |
|  | 8-(Hydroxymethyl)-3,8-dimethyl-2-oxooctahydro-3a,7-ethanoindene-4-carboxylic acid | Cid_75368792 |
|  | 1-Dehydroperuvinine | Cid_75368816 |
|  | Peruvinine | Cid_75368817 |
|  | Peruvic acid | Cid_75368818 |
|  | 2-((1S,3aR,4S,6S,7R,8aR)-1-Hydroxy-4,8a-dimethyloctahydro-1H-3a,6-epoxyazulen-7-yl)prop-2-enoic acid | Cid_75368819 |
|  | Isorosmanol | Cid_75411900 |
|  | Mucorisocoumarin B | Cid_75411939 |
|  | 6-Hydroxy-9a-methoxy-3,8a-dimethyl-5-methylene-4,4a,6,7,8,9-hexahydrobenzo[f]benzofuran-2-one | Cid_75528890 |
|  | (Z)-9,10,11-Trihydroxy-12-octadecenoic acid | Cid_75536014 |
|  | 1,3,4,5-Tetrahydrothiopyrano[4,3-b]indole-8-carboxylic acid | Cid_755947 |
|  | 4-(2-Pyridinylmethoxy)benzaldehyde | Cid_759667 |
|  | 2,4,6-Trimethyl-N-[3-(trifluoromethyl)phenyl]benzenesulfonamide | Cid_761523 |
|  | 2-(1-Hydroxy-1-methylethyl)-4a,8-dimethyldecalin-1,5-diol | Cid_76187279 |
|  | Benzoic acid + 2O, O-Hex | Cid_76211590 |
|  | Butyl (S)-3-hydroxybutyrate glucoside | Cid_76463851 |
|  | 4-(2,5-Dimethyl-1-pyrryl)phenylacetic acid | Cid_767161 |
|  | 2,5-Furandicarboxylic acid | Cid_76720 |
|  | Histidine | Cid_773 |
|  | Histamine | Cid_774 |
|  | Dimethyl sebacate | Cid_7829 |
|  | Leu-Gly | Cid_79070 |
|  | N-(3-Nitrobenzyl)cyclohexanamine | Cid_791978 |
|  | 2-(Diphenylphosphoryl)-N-ethylacetamide | Cid_796967 |
|  | 3-Pyridol | Cid_7971 |
|  | N-(5-Phenethyl-[1,3,4]thiadiazol-2-yl)benzamide | Cid_821478 |
|  | 4-Amino-2,3,6-trimethylphenol | Cid_82666 |
|  | Palatinose | Cid_83686 |
|  | D-Aspartic acid | Cid_83887 |
|  | Methionine sulfoxide | Cid_847 |
|  | N-Fructosyl phenylalanine | Cid_85152457 |
|  | 2,2'-Dihydroxy-4,4'-dimethoxybenzophenone | Cid_8570 |
|  | 3,4-Dihydroxymandelic acid | Cid_85782 |
|  | Imazamox | Cid_86137 |
|  | Syringaldehyde | Cid_8655 |
|  | 3,4-Dihydroxybenzaldehyde | Cid_8768 |
|  | N-(4-Methoxyphenyl)cyclohexaneacetamide | Cid_880925 |
|  | ML-099 | Cid_888706 |
|  | Anileridine | Cid_8944 |
|  | D-Proline | Cid_8988 |
|  | D-Psicose | Cid_90008 |
|  | Tryptophan | Cid_9060 |
|  | 3-Hydroxybutyrylcarnitine | Cid_90659885 |
|  | Ethyl 4-(4-methylpiperazin-1-yl)butanoate | Cid_914998 |
|  | 2-Amino-6-(4-methoxyphenyl)-4-phenylnicotinonitrile | Cid_915425 |
|  | 3-Methylbuphedrone | Cid_91696188 |
|  | 3,4-Methylenedioxy-N-benzylcathinone | Cid_91699622 |
|  | 2-Methyl-.alpha.-pyrrolidinobutiophenone | Cid_91704996 |
|  | JWH 251 3-methylphenyl isomer | Cid_91734833 |
|  | N-Acetylglucosaminylasparagine | Cid_91746260 |
|  | Imazapic | Cid_91770 |
|  | Arg-Leu | Cid_92146622 |
|  | Ser-Tyr | Cid_92259189 |
|  | Curcumenol | Cid_92281781 |
|  | Ser-Phe | Cid_92450011 |
|  | (.+/-.)-Vestitol | Cid_92503 |
|  | Leucic acid | Cid_92779 |
|  | Naringenin 7-O-beta-D-glucoside | Cid_92794 |
|  | Melezitose | Cid_92817 |
|  | .gamma.-Glutamylglutamic acid | Cid_92865 |
|  | 3-Indolyllactic acid | Cid_92904 |
|  | L-Arginine, methyl ester | Cid_92932 |
|  | Ile-Leu | Cid_92977173 |
|  | Aspartylphenylalanine | Cid_93078 |
|  | Nicotinic acid | Cid_938 |
|  | Vulgarin | Cid_94253 |
|  | Diffractaic acid | Cid_94870 |
|  | N-(1,1-Dimethylethyl)-3-pyridinemethanamine | Cid_961528 |
|  | tRNA containing 2'-O-methylguanosine | Cid_96373 |
|  | 1-Formylproline | Cid_96434 |
|  | Unguinol | Cid_97042256 |
|  | 3',4'-Dimethoxyflavonol | Cid_97143 |
|  | Tyr-Pro | Cid_9795637 |
|  | Ginsenoside F1 | Cid_9809542 |
|  | ONO-8711 | Cid_9824507 |
|  | L-1,2,3,4-Tetrahydro-beta-carboline-3-carboxylic acid | Cid_98285 |
|  | 1-O-.alpha.-Rhamnopyranosyl-(1''->6')-O-.beta.-D-glucopyranosyl-2-methoxy-4-acetylphenol | Cid_9832854 |
|  | Fructoselysine | Cid_9839580 |
|  | 4-O-D-Glucopyranosyl-p-coumaric acid | Cid_9840292 |
|  | Zeatin-9-glucoside | Cid_9842892 |
|  | 1-(p-Tolyl)cyclopropanecarbonitrile | Cid_98628 |
|  | Pantothenic acid | Cid_988 |
|  | (+)-trans-C75 | Cid_9881506 |
|  | Cyclo(val-pro) | Cid_98951 |
|  | 2-Indolinone, 3-hydroxy-3-phenacyl- | Cid_99051 |
|  | Phenol | Cid_996 |
|  | Dimethyl 2,4-bis(4-hydroxyphenyl)cyclobutane-1,3-dicarboxylate | Cid_9968607 |
|  | Alvespimycin | Cid_99719309 |
|  | 2-Hydroxy-3-methylbutyric acid | Cid_99823 |

**Table S4│Targets of the components in HRCR-MIAS identified by UHPLC Q-Exactive-MS**

| No. | PubCHEM CID | Target | No. | PubCHEM CID | Target |
| --- | --- | --- | --- | --- | --- |
|  | Cid_3861164 | ACE |  | Cid_13401 | EGLN2 |
|  | Cid_3861164 | HTR2B |  | Cid_13401 | HIF1AN |
|  | Cid_3861164 | HTR1A |  | Cid_13401 | KDM4D |
|  | Cid_3861164 | HTR2A |  | Cid_4994 | CYP3A4 |
|  | Cid_3861164 | HTR2C |  | Cid_1130 | TKT |
|  | Cid_3861164 | HTR7 |  | Cid_1130 | ADAMTS5 |
|  | Cid_3861164 | MMP3 |  | Cid_1130 | MMP14 |
|  | Cid_3861164 | MME |  | Cid_1130 | CYP26B1 |
|  | Cid_3861164 | IDO1 |  | Cid_1130 | CYP26A1 |
|  | Cid_3861164 | ITGAL |  | Cid_1130 | GPR35 |
|  | Cid_3861164 | ECE1 |  | Cid_1130 | CTSV |
|  | Cid_3861164 | TACR1 |  | Cid_40424009 | CAD |
|  | Cid_3861164 | SLC6A4 |  | Cid_40424009 | PPM1B |
|  | Cid_3861164 | ERAP2 |  | Cid_40424009 | PPP1CC |
|  | Cid_3861164 | EPHA2 |  | Cid_10685 | F2RL3 |
|  | Cid_3861164 | EPHB2 |  | Cid_2761529 | CDC45 |
|  | Cid_3861164 | EPHA5 |  | Cid_5291187 | PDE7B |
|  | Cid_3861164 | EPHA4 |  | Cid_5291187 | HPRT1 |
|  | Cid_3861164 | EPHA8 |  | Cid_5291187 | ALDH3A1 |
|  | Cid_3861164 | EPHA6 |  | Cid_5291187 | PTGDR |
|  | Cid_3861164 | EPHA7 |  | Cid_5291187 | FGFR3 |
|  | Cid_3861164 | EPHB3 |  | Cid_5291187 | CHRNA3 |
|  | Cid_3861164 | EPHA3 |  | Cid_5291187 | CDK5 |
|  | Cid_3861164 | EPHB1 |  | Cid_333703 | KIF11 |
|  | Cid_3861164 | EPHA1 |  | Cid_333703 | CCR8 |
|  | Cid_3861164 | EPHB6 |  | Cid_500 | GABRR1 |
|  | Cid_3861164 | ADRA2A |  | Cid_23615384 | SLC7A5 |
|  | Cid_3861164 | HTR1B |  | Cid_23615384 | SLC6A1 |
|  | Cid_3861164 | HTR1D |  | Cid_23615384 | GABRA3 |
|  | Cid_3861164 | ADRB1 |  | Cid_23615384 | SLC6A11 |
|  | Cid_3861164 | HTR1E |  | Cid_23615384 | SLC6A13 |
|  | Cid_3861164 | SLC6A2 |  | Cid_23615384 | BBOX1 |
|  | Cid_3861164 | HTR6 |  | Cid_1102 | CASP2 |
|  | Cid_3861164 | CTSL |  | Cid_637541 | TLR4 |
|  | Cid_3861164 | MC1R |  | Cid_637541 | TPMT |
|  | Cid_3861164 | EDNRA |  | Cid_637541 | NGFR |
|  | Cid_3861164 | EPHX2 |  | Cid_1742210 | LSS |
|  | Cid_3861164 | PPP5C |  | Cid_442916 | MSR1 |
|  | Cid_3861164 | PPP1CA |  | Cid_7829 | CPT1A |
|  | Cid_3861164 | TOP1 |  | Cid_7829 | LIMK2 |
|  | Cid_3861164 | EDNRB |  | Cid_7829 | ATP4B |
|  | Cid_3861164 | CTSC |  | Cid_7829 | TSPO |
|  | Cid_3861164 | CTSA |  | Cid_7829 | PTK6 |
|  | Cid_3861164 | CCR3 |  | Cid_100642075 | LYPLA1 |
|  | Cid_3861164 | PPARG |  | Cid_100642075 | LYPLA2 |
|  | Cid_3861164 | SORT1 |  | Cid_100642075 | CALM1 |
|  | Cid_3861164 | CPA1 |  | Cid_100642075 | CYP24A1 |
|  | Cid_3861164 | ACE2 |  | Cid_100642075 | CYP27A1 |
|  | Cid_3861164 | SLC6A3 |  | Cid_100642075 | SNCA |
|  | Cid_3861164 | PTPN1 |  | Cid_100642075 | F3 |
|  | Cid_3861164 | PTPN2 |  | Cid_100642075 | CETP |
|  | Cid_3861164 | ITGB1 |  | Cid_100642075 | NFE2L2 |
|  | Cid_3861164 | PDE7A |  | Cid_100642075 | DPP7 |
|  | Cid_3861164 | BCAT2 |  | Cid_1183 | EP300 |
|  | Cid_3861164 | HSD11B1 |  | Cid_773 | GFPT1 |
|  | Cid_3861164 | TTR |  | Cid_773 | KYNU |
|  | Cid_3861164 | NR4A1 |  | Cid_439232 | PEPD |
|  | Cid_3861164 | HSPA1A |  | Cid_439232 | TH |
|  | Cid_3861164 | NTSR1 |  | Cid_719436 | SLC5A1 |
|  | Cid_3861164 | CYP19A1 |  | Cid_6451888 | ITGAV |
|  | Cid_3861164 | KMO |  | Cid_6451888 | FURIN |
|  | Cid_3861164 | RBP4 |  | Cid_6451888 | PCSK6 |
|  | Cid_3861164 | PTGS1 |  | Cid_6451888 | HGFAC |
|  | Cid_3861164 | GSR |  | Cid_6451888 | RXRB |
|  | Cid_3861164 | PDE9A |  | Cid_6451888 | RXRG |
|  | Cid_3861164 | LDHA |  | Cid_6451888 | RARB |
|  | Cid_3861164 | AKR1C2 |  | Cid_6451888 | RXRA |
|  | Cid_3861164 | PTGS2 |  | Cid_6451888 | CFB |
|  | Cid_3861164 | EPHB4 |  | Cid_6451888 | CACNA1G |
|  | Cid_3861164 | PDGFRB |  | Cid_6451888 | NTSR2 |
|  | Cid_3861164 | FGFR1 |  | Cid_6451888 | CAMK2D |
|  | Cid_3861164 | KDM5A |  | Cid_6451888 | F9 |
|  | Cid_3861164 | KDM5B |  | Cid_229579 | SPHK1 |
|  | Cid_3861164 | KDM2B |  | Cid_229579 | KISS1R |
|  | Cid_3861164 | KDM4C |  | Cid_2787526 | RAD51 |
|  | Cid_3861164 | FABP4 |  | Cid_2787526 | CCKAR |
|  | Cid_3861164 | FABP5 |  | Cid_2787526 | ITGB1 |
|  | Cid_3861164 | PTGER3 |  | Cid_2787526 | ERAP1 |
|  | Cid_3861164 | PIK3CA |  | Cid_2787526 | TNNI3K |
|  | Cid_3861164 | MMP8 |  | Cid_2787526 | CSNK2A1 |
|  | Cid_3861164 | CTSB |  | Cid_2787526 | MKNK2 |
|  | Cid_3861164 | FABP1 |  | Cid_2787526 | RHOA |
|  | Cid_3861164 | MAP3K8 |  | Cid_2787526 | ITGB1 |
|  | Cid_3861164 | OPRM1 |  | Cid_2787526 | P4HTM |
|  | Cid_3861164 | GHSR |  | Cid_2787526 | ATIC |
|  | Cid_3861164 | FFAR1 |  | Cid_2787526 | NTRK2 |
|  | Cid_3861164 | CDC25B |  | Cid_2787526 | PTPsigma |
|  | Cid_3861164 | AMPD3 |  | Cid_2787526 | AURKC |
|  | Cid_3861164 | CMA1 |  | Cid_11778214 | ADRB2 |
|  | Cid_3861164 | PTPRF |  | Cid_11778214 | CDK6 |
|  | Cid_3861164 | DRD5 |  | Cid_11778214 | CDK3 |
|  | Cid_3861164 | ADRB3 |  | Cid_11778214 | DRD1 |
|  | Cid_3861164 | AKR1A1 |  | Cid_11778214 | CDK8 |
|  | Cid_3861164 | DAO |  | Cid_11778214 | GRK7 |
|  | Cid_3861164 | CXCL8 |  | Cid_11778214 | STK38 |
|  | Cid_3861164 | CREBBP |  | Cid_11778214 | HIPK4 |
|  | Cid_3861164 | ST3GAL1 |  | Cid_11778214 | OXSR1 |
|  | Cid_3861164 | KDM6B |  | Cid_11778214 | STK39 |
|  | Cid_3861164 | PTGIR |  | Cid_11778214 | MAP3K13 |
|  | Cid_3861164 | PDF |  | Cid_11778214 | ICK |
|  | Cid_92904 | ALPL |  | Cid_11778214 | MAP3K15 |
|  | Cid_92904 | CA2 |  | Cid_11778214 | MAST1 |
|  | Cid_92904 | CTNNB1 |  | Cid_11778214 | SBK1 |
|  | Cid_92904 | KDM2A |  | Cid_11778214 | HUNK |
|  | Cid_92904 | PHF8 |  | Cid_11778214 | PRPF4B |
|  | Cid_92904 | EGLN1 |  | Cid_11778214 | SNRK |
|  | Cid_92904 | FYN |  | Cid_10635 | HIF1A |
|  | Cid_92904 | MAOA |  | Cid_227752 | GRK2 |
|  | Cid_92904 | MAOB |  | Cid_734154 | SLC16A1 |
|  | Cid_10922465 | CES2 |  | Cid_734154 | PAM |
|  | Cid_10922465 | NR1H3 |  | Cid_734154 | NOTUM |
|  | Cid_10922465 | PRKCH |  | Cid_734154 | PLEC |
|  | Cid_10922465 | HSD11B2 |  | Cid_734154 | PTPRG |
|  | Cid_10922465 | PTPN11 |  | Cid_114679 | MPI |
|  | Cid_10922465 | PTPN6 |  | Cid_114679 | KCNJ1 |
|  | Cid_10922465 | FABP3 |  | Cid_114679 | VEGFA |
|  | Cid_10922465 | PTGES |  | Cid_91704996 | ADRA1A |
|  | Cid_10922465 | AKR1B10 |  | Cid_91704996 | DRD3 |
|  | Cid_10922465 | CDC25A |  | Cid_91704996 | HTR1F |
|  | Cid_10922465 | PREP |  | Cid_91696188 | ROCK2 |
|  | Cid_10922465 | FNTA |  | Cid_91696188 | PTK2 |
|  | Cid_10922465 | BCHE |  | Cid_43794772 | SLC18A2 |
|  | Cid_10922465 | ALOX5 |  | Cid_43794772 | CHRNA3 |
|  | Cid_10922465 | NOS2 |  | Cid_43794772 | GAPDH |
|  | Cid_10922465 | BACE1 |  | Cid_43794772 | CCNE2 |
|  | Cid_10922465 | FAAH |  | Cid_43794772 | ECE2 |
|  | Cid_10922465 | PPARD |  | Cid_43794772 | ATM |
|  | Cid_10922465 | NPC1L1 |  | Cid_43794772 | DNMT3A |
|  | Cid_10922465 | NR3C1 |  | Cid_43794772 | TNFRSF1A |
|  | Cid_10922465 | SERPINA6 |  | Cid_43794772 | HTR4 |
|  | Cid_10922465 | SIGMAR1 |  | Cid_43794772 | PLK3 |
|  | Cid_10922465 | SHBG |  | Cid_57486975 | KDM1A |
|  | Cid_10922465 | CYP17A1 |  | Cid_57486975 | CHKA |
|  | Cid_10922465 | PPARA |  | Cid_57486975 | NR1D1 |
|  | Cid_10922465 | SCD |  | Cid_101184 | YARS |
|  | Cid_10922465 | ADORA3 |  | Cid_101184 | SLCO1B1 |
|  | Cid_10922465 | MAPK3 |  | Cid_101184 | PARP15 |
|  | Cid_10922465 | RORC |  | Cid_101184 | TAS1R3 |
|  | Cid_10922465 | POLB |  | Cid_101184 | OXER1 |
|  | Cid_10922465 | PDE4D |  | Cid_101184 | CXCR1 |
|  | Cid_10922465 | HMGCR |  | Cid_101184 | CSNK2A2 |
|  | Cid_10922465 | PLA2G1B |  | Cid_101184 | ACER2 |
|  | Cid_10922465 | ACP1 |  | Cid_101184 | PDYN |
|  | Cid_10922465 | G6PD |  | Cid_5984 | ACLY |
|  | Cid_10922465 | CYP51A1 |  | Cid_5984 | PYGB |
|  | Cid_10922465 | SRD5A2 |  | Cid_5984 | PYGM |
|  | Cid_10922465 | TOP2A |  | Cid_2181 | CHRNB1 |
|  | Cid_10922465 | RORA |  | Cid_2181 | CHRNB4 |
|  | Cid_10922465 | ESR2 |  | Cid_2181 | CHRNB4 |
|  | Cid_10922465 | CD81 |  | Cid_2181 | CHRNB3 |
|  | Cid_10922465 | TERT |  | Cid_2181 | CHRNA2 |
|  | Cid_10922465 | AR |  | Cid_2181 | DPP9 |
|  | Cid_10922465 | PTGER2 |  | Cid_3542461 | HTR5A |
|  | Cid_10922465 | CES1 |  | Cid_3542461 | IFNAR1 |
|  | Cid_10922465 | PTGER1 |  | Cid_43183355 | CBX7 |
|  | Cid_10922465 | TNF |  | Cid_43183355 | CBX4 |
|  | Cid_10922465 | CNR1 |  | Cid_43183355 | BIRC2 |
|  | Cid_10922465 | SLC22A12 |  | Cid_43183355 | ITGB7 |
|  | Cid_10922465 | PTGDR2 |  | Cid_43183355 | BIRC3 |
|  | Cid_10922465 | HSD17B2 |  | Cid_43183355 | ADAMTS4 |
|  | Cid_10922465 | ESR1 |  | Cid_43183355 | NPPA |
|  | Cid_10922465 | PDPK1 |  | Cid_43183355 | LPAR3 |
|  | Cid_10922465 | PGR |  | Cid_43183355 | HLA-DRB1 |
|  | Cid_10922465 | MIF |  | Cid_43183355 | GALR1 |
|  | Cid_10922465 | PTGER4 |  | Cid_43183355 | GALR2 |
|  | Cid_10922465 | SRD5A1 |  | Cid_43183355 | RRM1 |
|  | Cid_10922465 | NR1I2 |  | Cid_43183355 | AGTR2 |
|  | Cid_10922465 | HSD17B3 |  | Cid_43183355 | ITGA4 |
|  | Cid_10922465 | NR1I3 |  | Cid_43183355 | BAD |
|  | Cid_57509469 | P11511 |  | Cid_43183355 | ADAM12 |
|  | Cid_57509469 | P54707 |  | Cid_10130 | GSTK1 |
|  | Cid_57509469 | Q53EL6 |  | Cid_10130 | IDE |
|  | Cid_57509469 | P35354 |  | Cid_68072 | CTBP2 |
|  | Cid_57509469 | O60885 |  | Cid_68072 | NEK2 |
|  | Cid_57509469 | P25440 |  | Cid_68072 | AXL |
|  | Cid_57509469 | P04054 |  | Cid_914998 | CHEK2 |
|  | Cid_57509469 | P04035 |  | Cid_914998 | POLR1A |
|  | Cid_57509469 | P67775 |  | Cid_914998 | PBK |
|  | Cid_57509469 | P10275 |  | Cid_914998 | PRMT6 |
|  | Cid_57509469 | P06401 |  | Cid_914998 | PRMT8 |
|  | Cid_57509469 | P17252 |  | Cid_914998 | NQO1 |
|  | Cid_57509469 | O75582 |  | Cid_914998 | ATAD2 |
|  | Cid_57509469 | P41252 |  | Cid_914998 | EBP |
|  | Cid_57509469 | P30307 |  | Cid_914998 | CPT1B |
|  | Cid_57509469 | P55085 |  | Cid_914998 | CHRNA1 |
|  | Cid_57509469 | O00311 |  | Cid_914998 | L3MBTL1 |
|  | Cid_57509469 | Q06124 |  | Cid_914998 | L3MBTL3 |
|  | Cid_57509469 | P37231 |  | Cid_914998 | TBK1 |
|  | Cid_57509469 | P23458 |  | Cid_914998 | SSTR4 |
|  | Cid_57509469 | P23219 |  | Cid_914998 | DYRK3 |
|  | Cid_57509469 | P36873 |  | Cid_914998 | CSNK1E |
|  | Cid_57509469 | P05067 |  | Cid_914998 | UTS2R |
|  | Cid_57509469 | P09488 |  | Cid_727889 | AOC2 |
|  | Cid_57509469 | P30304 |  | Cid_727889 | MAP2K3 |
|  | Cid_57509469 | P14555 |  | Cid_727889 | CSNK1G1 |
|  | Cid_57509469 | P52333 |  | Cid_727889 | RPS6KA1 |
|  | Cid_57509469 | P29597 |  | Cid_727889 | CSNK1A1 |
|  | Cid_57509469 | P05023 |  | Cid_727889 | KCNN1 |
|  | Cid_57509469 | P04150 |  | Cid_727889 | KCNN2 |
|  | Cid_57509469 | O60674 |  | Cid_727889 | CDK9 |
|  | Cid_57509469 | P15538 |  | Cid_727889 | BMP4 |
|  | Cid_57509469 | P19099 |  | Cid_727889 | PTPN13 |
|  | Cid_57509469 | Q15393 |  | Cid_92932 | AKT2 |
|  | Cid_57509469 | Q8IV61 |  | Cid_92932 | AKT3 |
|  | Cid_57509469 | Q16549 |  | Cid_3013625 | DLG4 |
|  | Cid_57509469 | Q5S007 |  | Cid_3013625 | XPNPEP1 |
|  | Cid_57509469 | P08183 |  | Cid_3013625 | XPNPEP2 |
|  | Cid_57509469 | P14174 |  | Cid_3013625 | GLO1 |
|  | Cid_57509469 | P09874 |  | Cid_3013625 | TNFRSF10A |
|  | Cid_57509469 | Q15078 |  | Cid_3013625 | NRP1 |
|  | Cid_57509469 | Q08499 |  | Cid_70912 | HCAR3 |
|  | Cid_57509469 | O00329 |  | Cid_45360204 | SLC29A1 |
|  | Cid_57509469 | P78527 |  | Cid_45360204 | SLC28A2 |
|  | Cid_57509469 | P42338 |  | Cid_45360204 | SLC5A2 |
|  | Cid_57509469 | Q96RJ0 |  | Cid_6993111 | CPB1 |
|  | Cid_57509469 | O14757 |  | Cid_5281166 | ITGAV |
|  | Cid_57509469 | P59538 |  | Cid_122334 | ADRA2C |
|  | Cid_57509469 | P11388 |  | Cid_122334 | HASPIN |
|  | Cid_57509469 | P30305 |  | Cid_122334 | SCN4A |
|  | Cid_11746594 | NR3C2 |  | Cid_122334 | PHLPP2 |
|  | Cid_11746594 | PRKCA |  | Cid_122334 | GRIN2B |
|  | Cid_11746594 | IL6 |  | Cid_122334 | SSTR2 |
|  | Cid_11746594 | GLUL |  | Cid_132999 | HCN4 |
|  | Cid_11746594 | GPBAR1 |  | Cid_132999 | HCN2 |
|  | Cid_11746594 | RASGRP3 |  | Cid_132999 | HCN1 |
|  | Cid_11746594 | DRD2 |  | Cid_132999 | GNRHR |
|  | Cid_11746594 | KMT5A |  | Cid_132999 | CDC42BPA |
|  | Cid_11746594 | ADAM17 |  | Cid_132999 | SGPL1 |
|  | Cid_11746594 | PRKCG |  | Cid_132999 | ADK |
|  | Cid_11746594 | PRKCD |  | Cid_132999 | PRKCI |
|  | Cid_11746594 | PRKCB |  | Cid_132999 | CALCRL |
|  | Cid_11746594 | PRKCE |  | Cid_132999 | ACKR3 |
|  | Cid_11746594 | PRKCQ |  | Cid_132999 | ADIPOR1 |
|  | Cid_11746594 | JAK3 |  | Cid_132999 | ADIPOR2 |
|  | Cid_11746594 | JAK1 |  | Cid_132999 | CCL5 |
|  | Cid_11746594 | JAK2 |  | Cid_132999 | PDE11A |
|  | Cid_11746594 | TYK2 |  | Cid_132999 | ITK |
|  | Cid_11746594 | PSEN2 |  | Cid_132999 | PKN2 |
|  | Cid_11746594 | TRPA1 |  | Cid_132999 | KARS |
|  | Cid_11746594 | NPY5R |  | Cid_132999 | NPY2R |
|  | Cid_11746594 | CA6 |  | Cid_132999 | KCNJ5 |
|  | Cid_11746594 | CA4 |  | Cid_132999 | MAP3K7 |
|  | Cid_11746594 | TAAR1 |  | Cid_132999 | PDGFRA |
|  | Cid_11746594 | CCR1 |  | Cid_442356 | DHCR7 |
|  | Cid_11746594 | ALOX12 |  | Cid_442356 | RBBP9 |
|  | Cid_11746594 | CHRNA3 |  | Cid_442356 | AADAT |
|  | Cid_11746594 | PDE10A |  | Cid_442356 | QDPR |
|  | Cid_11746594 | ITGAL |  | Cid_228537 | ESRRG |
|  | Cid_11746594 | CYP11B1 |  | Cid_228537 | NEK6 |
|  | Cid_11746594 | CYP11B2 |  | Cid_228537 | UQCRB |
|  | Cid_11746594 | MAPK14 |  | Cid_228537 | MYLK |
|  | Cid_11746594 | MAP2K1 |  | Cid_228537 | DBF4 |
|  | Cid_11746594 | MTNR1A |  | Cid_228537 | UPP1 |
|  | Cid_5280378 | IL2 |  | Cid_23930375 | TK2 |
|  | Cid_5280378 | ALDH2 |  | Cid_23930375 | APEX1 |
|  | Cid_5280378 | CA12 |  | Cid_23930375 | CBFB |
|  | Cid_5280378 | ADORA1 |  | Cid_9968607 | DUT |
|  | Cid_5280378 | ADORA2A |  | Cid_9968607 | MT-CO2 |
|  | Cid_5280378 | TBXAS1 |  | Cid_9968607 | NCOR2 |
|  | Cid_5280378 | MGAM |  | Cid_9968607 | HDAC11 |
|  | Cid_5280378 | ESRRA |  | Cid_9968607 | HDAC9 |
|  | Cid_5280378 | ESRRB |  | Cid_9968607 | HDAC10 |
|  | Cid_5280378 | ABCG2 |  | Cid_9968607 | FCER2 |
|  | Cid_5280378 | CA7 |  | Cid_5319562 | AKR1C4 |
|  | Cid_5280378 | CBR1 |  | Cid_5319562 | ALDH5A1 |
|  | Cid_5280378 | EGFR |  | Cid_5319562 | TMIGD3 |
|  | Cid_5280378 | TYR |  | Cid_464 | SLC16A3 |
|  | Cid_5280378 | XDH |  | Cid_439213 | SI |
|  | Cid_5280378 | PFKFB3 |  | Cid_14480057 | NUDT1 |
|  | Cid_5280378 | PTPRS |  | Cid_14480057 | PKM |
|  | Cid_5280378 | HSD17B1 |  | Cid_14480057 | OXTR |
|  | Cid_5280378 | ABCB1 |  | Cid_75190 | USP10 |
|  | Cid_5280378 | ALOX15 |  | Cid_75190 | USP13 |
|  | Cid_5280378 | TLR9 |  | Cid_75190 | C1R |
|  | Cid_5280378 | CA1 |  | Cid_75190 | KLK1 |
|  | Cid_5280378 | NOX4 |  | Cid_75190 | C1S |
|  | Cid_5280378 | PON1 |  | Cid_75190 | CTRB1 |
|  | Cid_5280378 | TNKS2 |  | Cid_75190 | CTSG |
|  | Cid_5280378 | TNKS |  | Cid_75190 | CTSH |
|  | Cid_5280378 | ACHE |  | Cid_4071159 | NMUR2 |
|  | Cid_5280378 | CA3 |  | Cid_4071159 | FUCA1 |
|  | Cid_5280378 | CA14 |  | Cid_240106 | F13A1 |
|  | Cid_5280378 | CA13 |  | Cid_240106 | XPO1 |
|  | Cid_5280378 | CA5B |  | Cid_2735705 | PARP14 |
|  | Cid_5280378 | CA5A |  | Cid_1042026 | CALCRL |
|  | Cid_5280378 | DHODH |  | Cid_1042026 | LYN |
|  | Cid_5280378 | STS |  | Cid_1042026 | STK17B |
|  | Cid_5280378 | ERCC5 |  | Cid_1042026 | LGMN |
|  | Cid_5280378 | FEN1 |  | Cid_1042026 | BRD9 |
|  | Cid_5280378 | RAF1 |  | Cid_1042026 | PDE1C |
|  | Cid_5280378 | CA9 |  | Cid_1042026 | MAP4K4 |
|  | Cid_7251185 | PGGT1B |  | Cid_1042026 | INSR |
|  | Cid_7251185 | JUN |  | Cid_1042026 | MAP3K12 |
|  | Cid_7251185 | VAV1 |  | Cid_1042026 | PLD1 |
|  | Cid_7251185 | TRPV4 |  | Cid_1042026 | PLD2 |
|  | Cid_7251185 | SLC2A1 |  | Cid_1042026 | TRHR |
|  | Cid_7251185 | ADCY1 |  | Cid_1042026 | GPR39 |
|  | Cid_7251185 | MDM2 |  | Cid_1042026 | PANK1 |
|  | Cid_7251185 | GLRA2 |  | Cid_1042026 | TXK |
|  | Cid_7251185 | F2RL1 |  | Cid_1042026 | TAOK2 |
|  | Cid_7251185 | IARS |  | Cid_5319022 | GPR55 |
|  | Cid_7251185 | SF3B3 |  | Cid_5319022 | CEL |
|  | Cid_7251185 | METAP2 |  | Cid_5319022 | RAPGEF4 |
|  | Cid_7251185 | CCR2 |  | Cid_10477119 | CX3CR1 |
|  | Cid_7251185 | MAPK10 |  | Cid_10477119 | LIPG |
|  | Cid_7251185 | CSNK1D |  | Cid_10477119 | F3 |
|  | Cid_7251185 | MAPK9 |  | Cid_10477119 | NFKB1 |
|  | Cid_7251185 | EPHX1 |  | Cid_10477119 | RELA |
|  | Cid_7251185 | PDCD4 |  | Cid_10477119 | ROS1 |
|  | Cid_7251185 | F2 |  | Cid_10477119 | F11 |
|  | Cid_7251185 | HCRTR2 |  | Cid_10477119 | PRSS3 |
|  | Cid_7251185 | HCRTR1 |  | Cid_10477119 | TYRO3 |
|  | Cid_7251185 | NLRP3 |  | Cid_5283028 | ATP12A |
|  | Cid_7251185 | IKBKB |  | Cid_445858 | CYP1A1 |
|  | Cid_7251185 | BCL2L1 |  | Cid_98951 | CAPN1 |
|  | Cid_7251185 | CYP2A6 |  | Cid_98951 | DPP8 |
|  | Cid_336327 | RET |  | Cid_98951 | WDR5 |
|  | Cid_336327 | LCK |  | Cid_98951 | CELA1 |
|  | Cid_336327 | ALK |  | Cid_98951 | GZMB |
|  | Cid_336327 | PIM1 |  | Cid_98951 | TMPRSS6 |
|  | Cid_336327 | PIM2 |  | Cid_98951 | HPN |
|  | Cid_336327 | KDR |  | Cid_98951 | ST14 |
|  | Cid_336327 | CHEK1 |  | Cid_98951 | TPSAB1 |
|  | Cid_336327 | WEE1 |  | Cid_98951 | MEN1 |
|  | Cid_336327 | GSK3B |  | Cid_10198252 | ITGB1 |
|  | Cid_336327 | KIT |  | Cid_3470698 | HPSE |
|  | Cid_336327 | MKNK1 |  | Cid_14448072 | TTL |
|  | Cid_336327 | MMP1 |  | Cid_3852139 | TDP1 |
|  | Cid_336327 | RPS6KA2 |  | Cid_3852139 | SIRT5 |
|  | Cid_336327 | MAPKAPK2 |  | Cid_3852139 | PPP2CA |
|  | Cid_336327 | PARP1 |  | Cid_3852139 | TAS2R31 |
|  | Cid_336327 | EIF4A1 |  | Cid_3852139 | LIPA |
|  | Cid_336327 | COMT |  | Cid_3852139 | PCSK7 |
|  | Cid_336327 | HTT |  | Cid_3852139 | S1PR2 |
|  | Cid_336327 | VCP |  | Cid_3852139 | MTAP |
|  | Cid_336327 | HDAC2 |  | Cid_3852139 | GAA |
|  | Cid_336327 | CCND3 |  | Cid_3852139 | MAN1B1 |
|  | Cid_336327 | ERN1 |  | Cid_3852139 | GSTM1 |
|  | Cid_336327 | LNPEP |  | Cid_3852139 | ATP2A1 |
|  | Cid_336327 | TYMS |  | Cid_3852139 | SREBF2 |
|  | Cid_336327 | CASP3 |  | Cid_3852139 | PRMT3 |
|  | Cid_336327 | CASP7 |  | Cid_354654 | SLC10A2 |
|  | Cid_336327 | MET |  | Cid_354654 | SLC37A4 |
|  | Cid_336327 | MTOR |  | Cid_354654 | MMEL1 |
|  | Cid_336327 | PIK3CD |  | Cid_354654 | CPT2 |
|  | Cid_336327 | PIK3CB |  | Cid_354654 | CAPN2 |
|  | Cid_336327 | PIK3CG |  | Cid_354654 | AMPD2 |
|  | Cid_336327 | GSTP1 |  | Cid_354654 | IL1B |
|  | Cid_336327 | GSTM2 |  | Cid_354654 | MMP10 |
|  | Cid_336327 | ABL1 |  | Cid_354654 | AVPR2 |
|  | Cid_336327 | PDE4A |  | Cid_354654 | AVPR1A |
|  | Cid_336327 | PDE4B |  | Cid_354654 | STK17A |
|  | Cid_336327 | PDE4C |  | Cid_354654 | QPCTL |
|  | Cid_336327 | PRKDC |  | Cid_354654 | CSK |
|  | Cid_336327 | HCK |  | Cid_354654 | ERG |
|  | Cid_336327 | ADORA2B |  | Cid_354654 | CAMKK2 |
|  | Cid_336327 | SRC |  | Cid_354654 | IGFBP3 |
|  | Cid_336327 | TUBB1 |  | Cid_354654 | CFTR |
|  | Cid_336327 | TUBB3 |  | Cid_354654 | OGA |
|  | Cid_336327 | CDK2 |  | Cid_354654 | ADA |
|  | Cid_336327 | CDK4 |  | Cid_354654 | GLRA1 |
|  | Cid_336327 | MMP7 |  | Cid_354654 | ADCY5 |
|  | Cid_336327 | MBD2 |  | Cid_354654 | LGALS3 |
|  | Cid_336327 | CDK5R1 |  | Cid_354654 | LGALS9 |
|  | Cid_336327 | CLK1 |  | Cid_354654 | HK2 |
|  | Cid_336327 | CLK3 |  | Cid_354654 | HK1 |
|  | Cid_336327 | DYRK2 |  | Cid_354654 | AMY2A |
|  | Cid_336327 | DGAT1 |  | Cid_354654 | TREH |
|  | Cid_336327 | PIK3CA |  | Cid_354654 | NEU2 |
|  | Cid_336327 | PLK1 |  | Cid_344187 | AGPAT2 |
|  | Cid_336327 | IRAK4 |  | Cid_344187 | HLA-DRB3 |
|  | Cid_336327 | AURKA |  | Cid_344187 | RGS4 |
|  | Cid_336327 | RPS6KA5 |  | Cid_344187 | MAP3K20 |
|  | Cid_336327 | MMP9 |  | Cid_344187 | ABL2 |
|  | Cid_336327 | MMP2 |  | Cid_344187 | TGFBR2 |
|  | Cid_336327 | GRIA1 |  | Cid_344187 | MAP2 |
|  | Cid_336327 | SYK |  | Cid_344187 | RIPK2 |
|  | Cid_336327 | ROCK2 |  | Cid_344187 | STK3 |
|  | Cid_336327 | ALPG |  | Cid_344187 | STK26 |
|  | Cid_181557 | BRD4 |  | Cid_344187 | SLC27A1 |
|  | Cid_181557 | BRD2 |  | Cid_344187 | C5AR1 |
|  | Cid_181557 | CDK1 |  | Cid_344187 | SLC27A4 |
|  | Cid_71694446 | P2RX7 |  | Cid_344187 | RIPK3 |
|  | Cid_71694446 | ADRA1D |  | Cid_344187 | HAO2 |
|  | Cid_71694446 | FADS1 |  | Cid_344187 | SSTR5 |
|  | Cid_71694446 | LIPE |  | Cid_344187 | SSTR1 |
|  | Cid_71694446 | APP |  | Cid_344187 | SSTR3 |
|  | Cid_71694446 | MTNR1B |  | Cid_344187 | UBA1 |
|  | Cid_14106072 | CTSD |  | Cid_344187 | YES1 |
|  | Cid_14106072 | CCR5 |  | Cid_344187 | FASN |
|  | Cid_14106072 | TRPV1 |  | Cid_344187 | BHMT |
|  | Cid_14106072 | CTSK |  | Cid_344187 | KYAT3 |
|  | Cid_14106072 | CTSS |  | Cid_344187 | ATP1A1 |
|  | Cid_14106072 | GABRB3 |  | Cid_344187 | FGF1 |
|  | Cid_14106072 |  |  | Cid_344187 | FGF2 |
|  | Cid_14106072 |  |  | Cid_344187 | LGALS4 |
|  | Cid_14106072 | GABRA2 |  | Cid_344187 | LGALS8 |
|  | Cid_14106072 |  |  | Cid_344187 | MTTP |
|  | Cid_14106072 | PIN1 |  | Cid_344187 | APOB |
|  | Cid_14106072 | FAP |  | Cid_344187 | MDM4 |
|  | Cid_14106072 | CHRM1 |  | Cid_344187 | ELOVL6 |
|  | Cid_14106072 | MC4R |  | Cid_344187 | C3AR1 |
|  | Cid_14106072 | CHRM4 |  | Cid_344187 | TEK |
|  | Cid_14106072 | CHRM5 |  | Cid_344187 | SELP |
|  | Cid_14106072 | PRSS1 |  | Cid_344187 | SELL |
|  | Cid_14106072 | CHRM3 |  | Cid_344187 | SLC46A1 |
|  | Cid_14106072 | NQO2 |  | Cid_344187 | FOLR1 |
|  | Cid_14106072 | GPR139 |  | Cid_344187 | GART |
|  | Cid_14106072 | GABRA1 |  | Cid_344187 | SLC19A1 |
|  | Cid_14106072 | HMOX1 |  | Cid_344187 | FOLR2 |
|  | Cid_14106072 | MPO |  | Cid_344187 | ITGB5 |
|  | Cid_14106072 | KCNA5 |  | Cid_344187 | FGFR2 |
|  | Cid_14106072 | FKBP1A |  | Cid_344187 | F5 |
|  | Cid_14106072 | GRM5 |  | Cid_344187 | LPAR1 |
|  | Cid_14106072 | SIRT2 |  | Cid_344187 | LPAR5 |
|  | Cid_14543627 | ABCC9 |  | Cid_344187 | ITGAV |
|  | Cid_14543627 | MAPK1 |  | Cid_344187 | PPP2R5A |
|  | Cid_14543627 | LRRK2 |  | Cid_344187 | SLC5A4 |
|  | Cid_14543627 | PYGL |  | Cid_344187 | HSPA8 |
|  | Cid_14543627 | DRD4 |  | Cid_344187 | MANBA |
|  | Cid_14543627 | JAK3 |  | Cid_344187 | CDA |
|  | Cid_14543627 | ICMT |  | Cid_344187 | HSPA5 |
|  | Cid_14543627 | PSMB5 |  | Cid_344187 | VARS |
|  | Cid_14543627 | TK1 |  | Cid_344187 | LARS |
|  | Cid_14543627 | VHL |  | Cid_344187 | AMY1A |
|  | Cid_14543627 | DYRK1A |  | Cid_344187 | HRAS |
|  | Cid_14543627 | MAPK11 |  | Cid_344187 | F7 |
|  | Cid_14543627 | KCNE1 |  | Cid_344187 | SGK1 |
|  | Cid_14543627 | IMPDH2 |  | Cid_344187 | PGF |
|  | Cid_14543627 | HSP90AB1 |  | Cid_344187 | FOS |
|  | Cid_14543627 | CDC7 |  | Cid_344187 | NEU3 |
|  | Cid_14543627 | PER2 |  | Cid_6482234 | FFAR4 |
|  | Cid_14543627 | LTA4H |  | Cid_6482234 | MMP16 |
|  | Cid_14543627 | PSEN1 |  | Cid_6482234 | MARS |
|  | Cid_14543627 | CCNA2 |  | Cid_6482234 | HNF4A |
|  | Cid_14543627 | PSEN2 |  | Cid_6482234 | PLA2G2E |
|  | Cid_14543627 | KCNH2 |  | Cid_6482234 | PLA2G2F |
|  | Cid_14543627 | KCNJ11 |  | Cid_6482234 | PLA2G2D |
|  | Cid_14543627 | PARP3 |  | Cid_138486 | Q9Y233 |
|  | Cid_14543627 | HPGDS |  | Cid_138486 | P31645 |
|  | Cid_14543627 | CXCR2 |  | Cid_138486 | P00734 |
|  | Cid_14543627 | PSMB2 |  | Cid_138486 | P07477 |
|  | Cid_14543627 | PSMB1 |  | Cid_735846 | EZR |
|  | Cid_14543627 | AKT1 |  | Cid_735846 | IKBKE |
|  | Cid_14543627 | CCNE1 |  | Cid_735846 | PDE1B |
|  | Cid_14543627 | CTRC |  | Cid_735846 | RIPK1 |
|  | Cid_14543627 | IL6ST |  | Cid_735846 | PTP4A3 |
|  | Cid_14543627 | TLR8 |  | Cid_135411330 | QRFPR |
|  | Cid_14543627 | MAP3K14 |  | Cid_135411330 | GPR84 |
|  | Cid_14543627 | TLR7 |  | Cid_135411330 | CCR9 |
|  | Cid_14543627 | TRPM8 |  | Cid_135411330 | CHAT |
|  | Cid_14543627 | CFD |  | Cid_16395956 | MAP2K4 |
|  | Cid_14543627 | ELANE |  | Cid_16395956 | MAPK13 |
|  | 14543627 | NTRK1 |  | Cid_16395956 | MAP2K7 |
|  | 14543627 | CYP2C9 |  | Cid_16395956 | MAPK12 |
|  | 14543627 | BRAF |  | Cid_16395956 | CENPE |
|  | Cid_168136 | CHRM2 |  | Cid_23983701 | PRKAA1 |
|  | Cid_168136 | TDO2 |  | Cid_23983701 | DNASE1L3 |
|  | Cid_168136 | ADH1A |  | Cid_182174 | FPR1 |
|  | Cid_168136 | CHRNA4 |  | Cid_182174 | PSENEN |
|  | Cid_168136 | ADH1C |  | Cid_6992304 | TACR3 |
|  | Cid_168136 | SLC5A7 |  | Cid_6992304 | GHRL |
|  | Cid_168136 | PABPC1 |  | Cid_6992304 | MC3R |
|  | Cid_168136 | MMP13 |  | Cid_13306588 | UBE2I |
|  | Cid_23815419 | CNR2 |  | Cid_1942420 | NMT1 |
|  | Cid_23815419 | PDE2A |  | Cid_1942420 | SLC6A15 |
|  | Cid_23815419 | KCNA3 |  | Cid_7408192 | ADAM8 |
|  | Cid_23815419 | IGF1R |  | Cid_7408192 | SPSB2 |
|  | Cid_23815419 | KCNK3 |  | Cid_880925 | CDC42 |
|  | Cid_23815419 | GPR88 |  | Cid_112477320 | HTR3B |
|  | Cid_23815419 | AKR1C3 |  | Cid_112477320 | USP14 |
|  | Cid_23815419 | HRH1 |  | Cid_124039 | GABRA6 |
|  | Cid_23815419 | BDKRB1 |  | Cid_124039 | CTSF |
|  | Cid_23815419 | SCN9A |  | Cid_114703 | MAPKAPK5 |
|  | Cid_23815419 | MAP3K5 |  | Cid_114703 | NDUFA4 |
|  | Cid_23815419 | SORD |  | Cid_114703 | NUAK1 |
|  | Cid_23815419 | ADRA1B |  | Cid_114703 | GUSB |
|  | Cid_23815419 | CASP1 |  | Cid_352039 | GPR142 |
|  | Cid_23815419 | CAPN1 |  | Cid_22824556 | GRB2 |
|  | Cid_23815419 | BRD3 |  | Cid_22824556 | GCLC |
|  | Cid_23815419 | CRHR1 |  | Cid_60208893 | DNTT |
|  | Cid_23815419 | OPRL1 |  | Cid_791978 | PKMYT1 |
|  | Cid_23815419 | CASP8 |  | Cid_2877 | MC5R |
|  | Cid_23815419 | MERTK |  | Cid_2877 | CCND2 |
|  | Cid_23815419 | PRKD1 |  | Cid_2877 | KHK |
|  | Cid_23815419 | EIF2AK3 |  | Cid_124433152 | PRMT5 |
|  | Cid_23815419 | AGTR1 |  | Cid_124433152 | NNMT |
|  | Cid_23815419 | CDK9 |  | Cid_75368727 | WNT3A |
|  | Cid_23815419 | SMYD2 |  | Cid_75368727 | LDLR |
|  | Cid_23815419 | F2R |  | Cid_75368727 | CCND1 |
|  | Cid_23815419 | ALOX5AP |  | Cid_75368727 | KCNJ6 |
|  | Cid_23815419 | P2RX3 |  | Cid_75368727 | CACNA1I |
|  | Cid_23815419 | SMO |  | Cid_5326566 | NGF |
|  | Cid_5318267 | AKR1B1 |  | Cid_100622832 | MAP2K2 |
|  | Cid_5280448 | ABCC1 |  | Cid_100622832 | SCARB1 |
|  | Cid_5280448 | CYP1B1 |  | Cid_100622832 | MLNR |
|  | Cid_5280448 | DUSP3 |  | Cid_100622832 | PIK3R1 |
|  | Cid_5280448 | PLAT |  | Cid_100622832 | MAP3K1 |
|  | Cid_5280448 | F10 |  | Cid_3156809 | MAK |
|  | Cid_5280448 | PLAU |  | Cid_3156809 | CDKL5 |
|  | Cid_92281781 | TRPV3 |  | Cid_3156809 | CDKL3 |
|  | Cid_92281781 | PTK2B |  | Cid_3156809 | PIK3C2G |
|  | Cid_92281781 | GSK3A |  | Cid_3156809 | MAP3K6 |
|  | Cid_92281781 | OPRD1 |  | Cid_3156809 | CDK13 |
|  | Cid_92281781 | OPRK1 |  | Cid_3156809 | CASK |
|  | Cid_92281781 | PTAFR |  | Cid_3156809 | PIP5K1C |
|  | Cid_7971 | DBH |  | Cid_3156809 | DSTYK |
|  | Cid_7971 | HDAC6 |  | Cid_3156809 | PRKAA2 |
|  | Cid_7971 | HDAC8 |  | Cid_3156809 | MAP2K6 |
|  | Cid_11007871 | DNM1 |  | Cid_3156809 | BLK |
|  | Cid_11007871 | NAAA |  | Cid_6175 | GRK1 |
|  | Cid_11007871 | SLC22A2 |  | Cid_6029 | P2RY6 |
|  | Cid_11007871 | SLC47A1 |  | Cid_6029 | OGT |
|  | Cid_11007871 | GRIN1 |  | Cid_6029 | HEXA |
|  | Cid_11007871 | GRIN2A |  | Cid_6029 | HEXB |
|  | Cid_11007871 | GRIN1 |  | Cid_6029 | P2RY4 |
|  | Cid_11007871 | NISCH |  | Cid_6029 | P2RY2 |
|  | Cid_138440 | HDAC3 |  | Cid_45359306 | UBLCP1 |
|  | Cid_138440 | SLC22A6 |  | Cid_45359306 | P2RY12 |
|  | Cid_138440 | FABP2 |  | Cid_45359306 | PTGIS |
|  | Cid_138440 | NR1H4 |  | Cid_45359306 | PTPA |
|  | Cid_138440 | VDR |  | Cid_5226097 | NPFFR1 |
|  | Cid_138440 | SLC6A12 |  | Cid_5226097 | NPFFR2 |
|  | Cid_138440 | KDM5C |  | Cid_5226097 | MLX |
|  | Cid_138440 | RNPEP |  | Cid_5226097 | BRCA1 |
|  | Cid_138440 | UGT2B7 |  | Cid_45783154 | LPAR6 |
|  | Cid_445144 | GPR17 |  | Cid_45783154 | LPAR2 |
|  | Cid_445144 | FBP1 |  | Cid_45783154 | LPAR4 |
|  | Cid_445144 | KCNN4 |  | Cid_56991460 | PDE8B |
|  | Cid_445144 | CACNA2D1 |  | Cid_56991460 | ITGA2 |
|  | Cid_445144 | SLC7A11 |  | Cid_11988273 | LANCL2 |
|  | Cid_445144 | KDM4E |  | Cid_11988273 | RAP1A |
|  | Cid_445144 | KDM3A |  | Cid_11128 | GGH |
|  | Cid_445144 | FTO |  | Cid_11128 | LIG1 |
|  | Cid_445144 | KDM4A |  | Cid_90659885 | SLC25A20 |
|  | Cid_273 | ODC1 |  | Cid_36606 | PLCG2 |
|  | Cid_821478 | GLS |  | Cid_36606 | BCL2A1 |
|  | Cid_821478 | GRM4 |  | Cid_36606 | CAMK2A |
|  | Cid_821478 | ERBB2 |  | Cid_12149038 | CHRNA5 |
|  | Cid_821478 | GABRA1 |  | Cid_92946 | CHRND |
|  | Cid_821478 | GABRA5 |  | Cid_92946 | SLC1A3 |
|  | Cid_676060 | CHRNA4 |  | Cid_715840 | IKBKG |
|  | Cid_676060 | XIAP |  | Cid_715840 | GCKR |
|  | Cid_676060 | GSTA1 |  | Cid_715840 | CSNK1G2 |
|  | Cid_676060 | DCTPP1 |  | Cid_715840 | CSNK1G3 |
|  | Cid_676060 | CSF1R |  | Cid_715840 | MAP3K19 |
|  | Cid_676060 | CASP4 |  | Cid_715840 | GPR183 |
|  | Cid_676060 | CASP9 |  | Cid_7408195 | NPY4R |
|  | Cid_676060 | CASP6 |  | Cid_7408195 | PRLR |
|  | Cid_676060 | BACE2 |  | Cid_7408195 | DPEP1 |
|  | Cid_676060 | MAPK8 |  | Cid_24096399 | ITGA2 |
|  | Cid_676060 | CACNA1C |  | Cid_24096399 | ITGAV |
|  | Cid_676060 | KLKB1 |  | Cid_22297431 | ADCY10 |
|  | Cid_676060 | KAT2B |  | Cid_22297431 | GPER1 |
|  | Cid_676060 | CDK2 |  | Cid_22297431 | GPR18 |
|  | Cid_676060 | PRTN3 |  | Cid_25320858 | GIPR |
|  | Cid_676060 | AOC1 |  | Cid_25320858 | GLP1R |
|  | Cid_676060 | PIM3 |  | Cid_25320858 | LIPC |
|  | Cid_676060 | TNNC1 |  | Cid_25320858 | PLA2G4B |
|  | Cid_676060 | MGLL |  | Cid_97143 | ST6GAL1 |
|  | Cid_676060 | TGFB1 |  | Cid_97143 | MAPT |
|  | Cid_676060 | NOS3 |  | Cid_97143 | CAMK2B |
|  | Cid_676060 | PLAA |  | Cid_97143 | DAPK1 |
|  | Cid_676060 | EPAS1 |  | Cid_97143 | PIK3R1 |
|  | Cid_676060 | AURKB |  | Cid_97143 | MPG |
|  | Cid_676060 | CDC25C |  | Cid_14606540 | NAE1 |
|  | Cid_676060 | RPS6KA3 |  | Cid_14606540 | PKN1 |
|  | Cid_676060 | NOD1 |  | Cid_915425 | NEK1 |
|  | Cid_676060 | NOD2 |  | Cid_480860 | TAOK1 |
|  | Cid_676060 | HSP90AA1 |  | Cid_480860 | TAOK3 |
|  | Cid_676060 | DYRK1B |  | Cid_480860 | ME1 |
|  | Cid_676060 | DAPK3 |  | Cid_480860 | ACVR1 |
|  | Cid_676060 | ASF1A |  | Cid_1972117 | SLC33A1 |
|  | Cid_676060 | SLC9A1 |  | Cid_1972117 | RPS27 |
|  | Cid_91734833 | CACNA1B |  | Cid_1972117 | CDK7 |
|  | Cid_91734833 | S1PR3 |  | Cid_1972117 | CAMK2G |
|  | Cid_91734833 | S1PR1 |  | Cid_1972117 | STK10 |
|  | Cid_91734833 | APLNR |  | Cid_1972117 | SLK |
|  | Cid_91734833 | PLA2G4A |  | Cid_1972117 | CDK16 |
|  | Cid_91734833 | TRPC6 |  | Cid_1972117 | MAPK15 |
|  | Cid_91734833 | TRPC3 |  | Cid_1972117 | CDK18 |
|  | Cid_91734833 | SLC6A7 |  | Cid_1972117 | DMPK |
|  | Cid_91734833 | SLC6A9 |  | Cid_1972117 | MAP4K2 |
|  | Cid_91734833 | CACNA1H |  | Cid_1972117 | MAPK7 |
|  | Cid_91734833 | CASR |  | Cid_1972117 | CDK11A |
|  | Cid_91734833 | BRS3 |  | Cid_1972117 | CDK17 |
|  | Cid_91734833 | FLT1 |  | Cid_1972117 | CDK11B |
|  | Cid_91734833 | GRM2 |  | Cid_1972117 | CDK15 |
|  | Cid_91734833 | HPGD |  | Cid_1972117 | DCLK3 |
|  | Cid_91734833 | PTPN22 |  | Cid_1972117 | CDK14 |
|  | Cid_91734833 | PANK3 |  | Cid_1972117 | STK33 |
|  | Cid_91734833 | GABRB3 |  | Cid_23928030 | SAE1 |
|  | Cid_91734833 | CACNA1D |  | Cid_9839580 | SETD7 |
|  | Cid_91734833 | CYP2C19 |  | Cid_13965473 | IGFBP6 |
|  | Cid_108312 | SHH |  | Cid_13965473 | IGFBP4 |
|  | Cid_108312 | S1PR5 |  | Cid_13965473 | IGFBP5 |
|  | Cid_108312 | S1PR4 |  | Cid_13965473 | IGFBP2 |
|  | Cid_108312 | PTGFR |  | Cid_13965473 | IGFBP1 |
|  | Cid_108312 | CPA3 |  | Cid_71751823 | SLC28A3 |
|  | Cid_108312 | CPB2 |  | Cid_71751823 | SLC22A8 |
|  | Cid_108312 | ADRA2B |  | Cid_4272 | RCE1 |
|  | Cid_108312 | TACR2 |  | Cid_4272 | BCL2L2 |
|  | Cid_108312 | RASGRP1 |  | Cid_5283445 | GPR119 |
|  | Cid_108312 | SLC13A5 |  | Cid_5283445 | SLC8A1 |
|  | Cid_22278 | ADH1B |  | Cid_586372 | INSRR |
|  | Cid_1201529 | FDFT1 |  | Cid_586372 | ACVRL1 |
|  | Cid_19374862 | AOC3 |  | Cid_118796498 | KCNQ1 |
|  | Cid_4250163 | HDAC1 |  | Cid_118796498 | FKBP5 |
|  | Cid_4250163 | NAMPT |  | Cid_118796498 | PORCN |
|  | Cid_4250163 | MCL1 |  | Cid_118796498 | INCENP |
|  | Cid_4250163 | CISD1 |  | Cid_118796498 | ORAI1 |
|  | Cid_4250163 | SOAT1 |  | Cid_12599 | FHIT |
|  | Cid_759667 | MALT1 |  | Cid_12599 | QARS |
|  | Cid_759667 | ICAM1 |  | Cid_12599 | P2RY11 |
|  | Cid_759667 | SELE |  | Cid_12599 | RARS |
|  | Cid_759667 | TGM2 |  | Cid_12599 | DNPH1 |
|  | Cid_759667 | KCNK2 |  | Cid_12599 | P2RX1 |
|  | Cid_759667 | TYMP |  | Cid_12599 | PARG |
|  | Cid_759667 | CCNB3 |  | Cid_12599 | RNASEL |
|  | Cid_759667 | TDP2 |  | Cid_12599 | RNASE2 |
|  | Cid_759667 | CCNC |  | Cid_12599 | RNASE1 |
|  | Cid_759667 | ASAH1 |  | Cid_12599 | ENPP1 |
|  | Cid_759667 | FLT4 |  | Cid_12599 | ASNS |
|  | Cid_759667 | VCAM1 |  | Cid_12599 | EIF4E |
|  | Cid_759667 | PLK4 |  | Cid_12599 | DTYMK |
|  | Cid_759667 | SCN10A |  | Cid_12599 | FDPS |
|  | Cid_759667 | ROCK1 |  | Cid_12599 | TRPM2 |
|  | Cid_759667 | FLT3 |  | Cid_12599 | AMD1 |
|  | Cid_759667 | KCNK9 |  | Cid_12599 | SRM |
|  | Cid_759667 | PARP10 |  | Cid_44533020 | CCND3 |
|  | Cid_759667 | PARP4 |  | Cid_44533020 | PIK3C3 |
|  | Cid_759667 | QPCT |  | Cid_44533020 | MST1R |
|  | Cid_759667 | PLK2 |  | Cid_44533020 | TIE1 |
|  | Cid_759667 | KDM4B |  | Cid_44533020 | STAT6 |
|  | Cid_759667 | HRH4 |  | Cid_73399 | SOAT2 |
|  | Cid_759667 | IDH1 |  | Cid_45933916 | CNOT7 |
|  | Cid_759667 | PRKACA |  | Cid_45933916 | HPD |
|  | Cid_759667 | CYP1A2 |  | Cid_2369 | SPHK2 |
|  | Cid_759667 | PRF1 |  | Cid_2369 | NPY1R |
|  | Cid_14079 | PTPRC |  | Cid_29980616 | TP53 |
|  | Cid_5311079 | GRIK1 |  | Cid_29980616 | PTGES2 |
|  | Cid_5311079 | SLC1A2 |  | Cid_29980616 | HSD17B7 |
|  | Cid_5311079 | GRM3 |  | Cid_29980616 | DAGLA |
|  | Cid_5311079 | GRM8 |  | Cid_29980616 | ENPP2 |
|  | Cid_5311079 | GRM1 |  | Cid_29980616 | DAGLB |
|  | Cid_5311079 | GRM6 |  | Cid_7020001 | MAGI3 |
|  | Cid_5311079 | GRIK2 |  | Cid_7020001 | PSMB8 |
|  | Cid_5311079 | SLC1A1 |  | Cid_7020001 | PSMB9 |
|  | Cid_5311079 | GRIK5 |  | Cid_181526 | PPM1A |
|  | Cid_5311079 | GRIA4 |  | Cid_181526 | NEU4 |
|  | Cid_5311079 | GRIK3 |  | Cid_54680116 | PSMG3 |
|  | Cid_5311079 | GRIA2 |  | Cid_21721881 | KLK2 |
|  | Cid_5311079 | ENPEP |  | Cid_21721881 | PGD |
|  | Cid_5311079 | GRM7 |  | Cid_21721881 | ST3GAL3 |
|  | Cid_5311079 | PLG |  | Cid_21721881 | FUT4 |
|  | Cid_439227 | ITGA2B |  | Cid_21721881 | STAT1 |
|  | Cid_439227 | PLA2G2A |  | Cid_21721881 | MT-ND4 |
|  | Cid_439227 | PLA2G5 |  | Cid_443955 | FCGR1A |
|  | Cid_439227 | OAT |  | Cid_443955 | NMBR |
|  | Cid_439227 | LAP3 |  | Cid_94701 | MAP3K9 |
|  | Cid_439227 | SLC18A3 |  | Cid_135398570 | RAC1 |
|  | Cid_439227 | NOS1 |  | Cid_3105 | TNK2 |
|  | Cid_439227 | GABBR2 |  | Cid_3105 | DLK1 |
|  | Cid_774 | HRH2 |  | Cid_3105 | PBRM1 |
|  | Cid_774 | HRH3 |  | Cid_3105 | BRDT |
|  | Cid_774 | CHRNA7 |  | Cid_3105 | BRD7 |
|  | Cid_53394341 | ARG1 |  | Cid_3105 | TAF1 |
|  | Cid_132862 | SLC15A1 |  | Cid_3105 | BRD8 |
|  | Cid_132862 | DDAH1 |  | Cid_3105 | CD74 |
|  | Cid_132862 | ANPEP |  | Cid_3105 | SCN1A |
|  | Cid_132862 | PTPRA |  | Cid_8944 | MCHR2 |
|  | Cid_132862 | CPN1 |  | Cid_8944 | ABHD6 |
|  | Cid_2060575 | CYP2D6 |  | Cid_8944 | ARHGDIA |
|  | Cid_2060575 | DPP4 |  | Cid_8944 | PGGT1B |
|  | Cid_2060575 | RCOR1 |  | Cid_8944 | CHIA |
|  | Cid_6285057 | AHR |  | Cid_8944 | MGAT2 |
|  | Cid_6285057 | HLCS |  | Cid_24739240 | PRKAG1 |
|  | Cid_761523 | CDK7 |  | Cid_119058050 | PRLHR |
|  | Cid_761523 | GABRB3 |  | Cid_119058050 | MGAT3 |
|  | Cid_761523 | GABRA2 |  | Cid_1547484 | ACACA |
|  | Cid_761523 | CDK1 |  | Cid_1547484 | OGFRL1 |
|  | Cid_761523 | SCN2A |  | Cid_102591297 | ACVR1B |
|  | Cid_761523 | CCR4 |  | Cid_64981 | SMARCA2 |
|  | Cid_761523 | PIP4K2C |  | Cid_64981 | PITRM1 |
|  | Cid_761523 | P2RY1 |  | Cid_64981 | NOX1 |
|  | Cid_761523 | DHFR |  | Cid_9842892 | SETD2 |
|  | Cid_15730832 | LTB4R |  | Cid_9842892 | LGALS7 |
|  | Cid_15730832 | IMPDH1 |  | Cid_10475115 | SLC6A5 |
|  | Cid_15730832 | KDM7A |  | Cid_145454234 | NPR1 |
|  | Cid_15730832 | THRA |  | Cid_145454234 | NPR3 |
|  | Cid_15730832 | THRB |  | Cid_110208996 | MAP3K11 |
|  | Cid_15730832 | PDE5A |  | Cid_4485701 | BRD1 |
|  | Cid_15730832 | TBXA2R |  | Cid_4485701 | ATAD2B |
|  | Cid_15730832 | PDE6D |  | Cid_4485701 | TAF1L |
|  | Cid_15730832 | RARG |  | Cid_4485701 | BRPF3 |
|  | Cid_15730832 | RARA |  | Cid_4485701 | EIF2AK1 |
|  | Cid_15730832 | PTPN7 |  | Cid_56773937 | GABRA4 |
|  | Cid_15730832 | GRK6 |  | Cid_56773937 | GABRA3 |
|  | Cid_15730832 | AMPD1 |  | Cid_56773937 | GABRA2 |
|  | Cid_15730832 | SERPINE1 |  | Cid_56773937 | ALKBH3 |
|  | Cid_15730832 | FOLH1 |  | Cid_56773937 | PAK1 |
|  | Cid_15730832 | BMP1 |  | Cid_99719309 | DDOST |
|  | Cid_338 | LDHB |  | Cid_99719309 | KLK3 |
|  | Cid_338 | AKR1C1 |  | Cid_99719309 | TRIM24 |
|  | Cid_338 | FUT7 |  | Cid_23874473 | CD22 |
|  | Cid_338 | ALB |  | Cid_442813 | MGMT |
|  | Cid_4177311 | CHUK |  | Cid_71317118 | ABHD12 |
|  | Cid_4177311 | PIK3CD |  | Cid_71317118 | ABHD16A |
|  | Cid_4177311 | RPS6KB1 |  | Cid_8768 | DUSP1 |
|  | Cid_4177311 | BCL2 |  | Cid_519541 | RNASEH1 |
|  | Cid_4177311 | CCNT1 |  | Cid_45783053 | EIF4H |
|  | Cid_4177311 | BRPF1 |  | Cid_12227668 | MDH2 |
|  | Cid_4177311 | GCK |  | Cid_1670 | PCNA |
|  | Cid_4177311 | GYS1 |  | Cid_268297 | PIN4 |
|  | Cid_4177311 | LIMK1 |  | Cid_2826719 | KAT5 |
|  | Cid_4177311 | PRKCZ |  | Cid_2826719 | ELAVL1 |
|  | Cid_4177311 | STAT3 |  | Cid_6420119 | DUSP22 |
|  | Cid_4177311 | HSP90B1 |  | Cid_6420119 | HIPK3 |
|  | Cid_4177311 | KCNN3 |  | Cid_6420119 | UCHL1 |
|  | Cid_4177311 | ERBB4 |  | Cid_16095194 | CCR6 |
|  | Cid_4177311 | PARP2 |  | Cid_16095194 | ATP6AP1 |
|  | Cid_4177311 | SCN5A |  | Cid_4524287 | HAGH |
|  | Cid_4177311 | BDKRB2 |  | Cid_667639 | YWHAG |
|  | Cid_4177311 | TRAP1 |  | Cid_667639 | HSD17B14 |
|  | Cid_4177311 | ATR |  | Cid_11960529 | PRKX |
|  | Cid_938 | HCAR2 |  | Cid_40634 | PHLPP1 |
|  | Cid_938 | DDO |  | Cid_23983705 | GALK1 |
|  | Cid_938 | SIRT3 |  | Cid_5380645 | BIRC5 |
|  | Cid_419490 | AHCY |  | Cid_259632 | MMP25 |
|  | Cid_4472712 | REN |  | Cid_259632 | ADAM10 |
|  | Cid_4472712 | HLA-A |  | Cid_888706 | RPA1 |
|  | Cid_4472712 | GNPAT |  | Cid_888706 | EPRS |
|  | Cid_4472712 | DNPEP |  | Cid_888706 | CAMKK1 |
|  | Cid_4472712 | FPR2 |  | Cid_440043 | ITPR3 |
|  | Cid_4472712 | PGC |  | Cid_440043 | ITPR1 |
|  | Cid_4472712 | EGLN3 |  | Cid_440043 | INPP5A |
|  | Cid_4472712 | SIRT1 |  | Cid_2872935 | GANAB |
|  | Cid_4472712 | PLA2G2C |  | Cid_5281707 | FGR |
|  | Cid_4472712 | PLA2G10 |  | Cid_16376440 | CCNE1 |
|  | Cid_4472712 | UGCG |  | Cid_92503 | ALOX15B |
|  | Cid_4472712 | GBA |  | Cid_10639 | EIF2AK2 |
|  | Cid_4472712 | GBA2 |  | Cid_51136263 | PTPN9 |
|  | Cid_4472712 | PGA5 |  | Cid_5976625 | MPL |
|  | Cid_4472712 | SAE1 |  | Cid_42905 | PAK4 |
|  | Cid_4472712 | CTSE |  | Cid_42905 | LPL |
|  | Cid_4472712 | ADAM9 |  | Cid_139292107 | MAG |
|  | Cid_199 | PAOX |  | Cid_38361084 | PLCG1 |
|  | Cid_18687359 | S100B |  | Cid_38361084 | BMX |
|  | Cid_18687359 | PRCP |  | Cid_38361084 | COQ8B |
|  | Cid_18687359 | PNMT |  | Cid_5329099 | DUSP26 |
|  | Cid_91746260 | KMT2A |  | Cid_5283007 | SCP2 |
|  | Cid_91746260 | DOT1L |  | Cid_5283007 | GCG |
|  | Cid_91746260 | SUV39H1 |  | Cid_5283007 | KEAP1 |
|  | Cid_91746260 | DNMT1 |  | Cid_5986673 | PHKG2 |
|  | Cid_91746260 | INMT |  | Cid_94870 | ADH5 |
|  | Cid_91746260 | EZH2 |  | Cid_45782803 | PADI1 |
|  | Cid_91746260 | EZH1 |  | Cid_45782803 | PADI2 |
|  | Cid_91746260 | SETDB1 |  | Cid_45782803 | PADI3 |
|  | Cid_91746260 | CARM1 |  | Cid_45782803 | PADI4 |
|  | Cid_91746260 | PRMT1 |  | Cid_13966127 | GABRA1 |
|  | Cid_91746260 | EHMT1 |  | Cid_21588208 | PIK3C2B |
|  | Cid_91746260 | EHMT2 |  | Cid_21588208 | POLK |
|  | Cid_91746260 | DNMT3B |  | Cid_75411900 | STAT5A |
|  | Cid_439600 | ABAT |  | Cid_129009008 | ABCB11 |
|  | Cid_119507 | MMP12 |  | Cid_129009008 | ENGASE |
|  | Cid_119507 | HDAC7 |  | Cid_439211 | B4GALT1 |
|  | Cid_119507 | NAALAD2 |  | Cid_439211 | P2RY14 |
|  | Cid_119507 | HTR3A |  | Cid_3548445 | JAK1 |
|  | Cid_119507 | CXCR4 |  | Cid_10111831 | GRIN2D |
|  | Cid_119507 | ILK |  | Cid_135403648 | MTHFD2 |
|  | Cid_72503 | ADH4 |  | Cid_135403648 | FPGS |
|  | Cid_72503 | ALDH1A1 |  | Cid_135403648 | MTR |
|  | Cid_72503 | CCND1 |  | Cid_75368782 | FARS2 |
|  | Cid_16724 | METAP1 |  | Cid_75368782 | KLK5 |
|  | Cid_10976240 | GABBR1 |  | Cid_75368782 | PPOX |
|  | Cid_10976240 | HAO1 |  | Cid_24826799 | DDR2 |
|  | Cid_16395991 | SQLE |  | Cid_24826799 | DDR1 |
|  | Cid_16395991 | NR1H2 |  | Cid_24826799 | PIK3C2A |
|  | Cid_16395991 | GCGR |  | Cid_24826799 | SFRP1 |
|  | Cid_16395991 | POLA1 |  | Cid_16395819 | CCL2 |
|  | Cid_135398638 | GDA |  | Cid_2601 | SCD5 |
|  | Cid_135398638 | PNP |  | Cid_5281727 | PRKACB |
|  | Cid_454892 | PDGFRA |  | Cid_5281727 | FRK |
|  | Cid_454892 | PDE3A |  | Cid_5281727 | STK36 |
|  | Cid_454892 | PDE3B |  | Cid_5281727 | GAK |
|  | Cid_454892 | BAZ2B |  | Cid_5281727 | TNIK |
|  | Cid_454892 | PGK1 |  | Cid_5281727 | RPS6KA6 |
|  | Cid_454892 | CECR2 |  | Cid_5281727 | CDC42BPB |
|  | Cid_454892 | BAZ2A |  | Cid_5281727 | NLK |
|  | Cid_454892 | TGFBR1 |  | Cid_5281727 | ACVR2B |
|  | Cid_454892 | BTK |  | Cid_5281727 | CSNK1A1L |
|  | Cid_454892 | PPIA |  | Cid_5281727 | CIT |
|  | Cid_454892 | KIF20A |  | Cid_5281727 | CDC42BPG |
|  | Cid_454892 | ZAP70 |  | Cid_5281727 | LATS2 |
|  | Cid_454892 | NPBWR1 |  | Cid_5281727 | STK32B |
|  | Cid_454892 | PI4KB |  | Cid_44525694 | GRK5 |
|  | Cid_454892 | CLK4 |  | Cid_371682 | CCNA2 |
|  | Cid_454892 | CLK2 |  | Cid_371682 | SHC1 |
|  | Cid_1533806 | MB |  | Cid_371682 | DDX3X |
|  | Cid_1533806 | KCNMA1 |  | Cid_371682 | PLAUR |
|  | Cid_1533806 | HDAC5 |  | Cid_371682 | SLC8B1 |
|  | Cid_1533806 | HDAC4 |  | Cid_371682 | SMS |
|  | Cid_1533806 | EEF2K |  | Cid_371682 | PRMT7 |
|  | Cid_1533806 | NAT1 |  | Cid_371682 | NR4A2 |
|  | Cid_1533806 | PLA2G7 |  | Cid_371682 | MMP15 |
|  | Cid_1533806 | PRKAB1 |  | Cid_371682 | MMP26 |
|  | Cid_1533806 | PRKD2 |  | Cid_371682 | PDE6A |
|  | Cid_5374527 | GLI2 |  | Cid_371682 | CD274 |
|  | Cid_5374527 | GLI1 |  | Cid_371682 | CAMK1 |
|  | Cid_525811 | MCHR1 |  | Cid_371682 | CAMK4 |
|  | Cid_525811 | TTK |  | Cid_371682 | DKK1 |
|  | Cid_525811 | NCOR1 |  | Cid_371682 | ASIC3 |
|  | Cid_525811 | ACACB |  | Cid_371682 | RORB |
|  | Cid_525811 | CD38 |  | Cid_371682 | RPS6KA4 |
|  | Cid_525811 | PDK1 |  | Cid_371682 | MAPK6 |
|  | Cid_525811 | CXCR3 |  | Cid_371682 | MYLK4 |
|  | Cid_525811 | PROC |  | Cid_371682 | GABRA6 |
|  | Cid_525811 | MELK |  | Cid_371682 | PTPRCAP |
|  | Cid_525811 | MPEG1 |  | Cid_371682 | GPR34 |
|  | Cid_525811 | SLC2A3 |  | Cid_371682 | P2RY10 |
|  | Cid_525811 | SLC2A2 |  | Cid_371682 | GPR174 |
|  | Cid_525811 | CCKBR |  | Cid-11471482 | CPM |
|  | Cid_135591005 | TPO | - | - | - |

**Table S5│ The common targets of diseases**

| No. | Target | No. | Target | No. | Target | No. | Target |
| --- | --- | --- | --- | --- | --- | --- | --- |
|  | MSH6 |  | VARS1 |  | TRE-TTC3-1 |  | CLDN4 |
|  | MSH2 |  | EIF2B4 |  | FBP1 |  | POSTN |
|  | MLH1 |  | LARS1 |  | TRIM21 |  | ETS1 |
|  | APC |  | MT-TP |  | ROCR |  | BIRC2 |
|  | TP53 |  | USP20 |  | LTF |  | LRP6 |
|  | PALB2 |  | SYVN1 |  | TRAP1 |  | LINC02605 |
|  | POLD1 |  | MT-ND5 |  | ADGRE5 |  | TMEFF2 |
|  | CDH1 |  | LSM12 |  | TNFSF13 |  | WNT2 |
|  | MUTYH |  | LIMK2 |  | HLA-DRA |  | MIF |
|  | PTEN |  | SETD1B |  | RPL18 |  | CLDN1 |
|  | KRAS |  | USP19 |  | RAD21 |  | STAT6 |
|  | EGFR |  | BCL2L14 |  | CBFA2T3 |  | AMACR |
|  | BRAF |  | VNN1 |  | TUBA1A |  | RUNX2 |
|  | SMAD4 |  | PCK2 |  | PDCD6 |  | LZTS1 |
|  | CTNNB1 |  | SEC22B |  | FOXP1 |  | H2AX |
|  | CDKN2A |  | ZBTB33 |  | APBA1 |  | AGER |
|  | H19 |  | MAFK |  | ELOB |  | SERPINA1 |
|  | CCND1 |  | SEC16A |  | USP1 |  | CXCR2 |
|  | MIR21 |  | CTSG |  | RTEL1-TNFRSF6B |  | TJP1 |
|  | FGFR3 |  | NOL6 |  | ATOH1 |  | TXN |
|  | DICER1 |  | AAMP |  | KRT9 |  | FOXE1 |
|  | FGFR2 |  | ASH2L |  | IKZF3 |  | LGR5 |
|  | MRE11 |  | LINC01475 |  | TUBB2A |  | IL11 |
|  | TGFBR2 |  | PDK3 |  | ZFP36 |  | MIR17HG |
|  | TERT |  | GAP43 |  | MRTFA |  | MIR449A |
|  | CCAT1 |  | DDX24 |  | SOCS2 |  | IL4R |
|  | GAS5 |  | RHBDD1 |  | COPS6 |  | TIAM1 |
|  | MYC |  | GPANK1 |  | TNFSF15 |  | SPHK1 |
|  | CDK4 |  | NLRP6 |  | MIR655 |  | CSNK1A1 |
|  | XIST |  | MIR1226 |  | AICDA |  | NOD2 |
|  | EP300 |  | PSMD3 |  | CDC37 |  | S100A9 |
|  | FBXW7 |  | RAE1 |  | GRWD1 |  | MYB |
|  | BAX |  | MIR523 |  | DHX9 |  | EPO |
|  | MIR31 |  | IMPA2 |  | CPSF6 |  | ADAM17 |
|  | BAP1 |  | CDH23 |  | CUL4B |  | LAMC2 |
|  | CDKN1B |  | DNAJB6 |  | TFAM |  | CEACAM6 |
|  | MIR34A |  | MIR4728 |  | UGDH |  | SLC19A1 |
|  | MIR155 |  | UTS2 |  | SRSF1 |  | TNFRSF1B |
|  | DCC |  | STMN3 |  | IL23R |  | HNF4A |
|  | LINC-ROR |  | PHKA2 |  | IL13RA1 |  | WEE1 |
|  | PPARG |  | LUC7L |  | ITGA4 |  | SOD2-OT1 |
|  | MIR20A |  | NUDT21 |  | MACF1 |  | CDX1 |
|  | MIR34C |  | PSMA3 |  | SIRT6 |  | MAPK9 |
|  | NFE2L2 |  | USP12 |  | GSK3A |  | MIR133A1 |
|  | MIR29A |  | NDUFA4 |  | IRF8 |  | NTN1 |
|  | MIR18A |  | DDOST |  | TUBB6 |  | CCL20 |
|  | KCNQ1OT1 |  | NOC4L |  | DDX17 |  | HSP90B1 |
|  | STAT3 |  | NCF4 |  | PARK7 |  | IL13 |
|  | MIR223 |  | GALK1 |  | ATF6 |  | MCM7 |
|  | MTOR |  | VBP1 |  | PITX1 |  | CASP1 |
|  | VEGFA |  | IL17RD |  | SLX1A-SULT1A3 |  | DVL1 |
|  | PIK3R1 |  | MIR1260B |  | IL21 |  | CXCR1 |
|  | SMAD7 |  | WDR62 |  | MTSS1 |  | AHR |
|  | MIR100 |  | IL10RB |  | CXCL2 |  | ICOSLG |
|  | MIR215 |  | PRTN3 |  | HUWE1 |  | CTBP1 |
|  | MDM2 |  | ZGPAT |  | MUS81 |  | SUZ12 |
|  | MIR320A |  | WDR20 |  | SDC2 |  | S100A8 |
|  | PTGS2 |  | IL1R2 |  | TACR1 |  | TMPRSS2 |
|  | RHOA |  | DAP |  | NLRP1 |  | ALOX15 |
|  | MIR19A |  | PLXDC2 |  | ABL2 |  | SDC1 |
|  | MIR214 |  | PPP6R1 |  | RPL13 |  | SFTA3 |
|  | JUN |  | ZFP90 |  | CAPZB |  | HMGA2 |
|  | BCL2 |  | C1QTNF4 |  | ABCF2 |  | HIC1 |
|  | IDH1 |  | ALDH18A1 |  | TUBA1C |  | SYNE1 |
|  | MIR146B |  | CBLL1 |  | ST6GALNAC1 |  | GJA1 |
|  | MIR15B |  | CARD9 |  | CAMK2G |  | MIR551A |
|  | TLR2 |  | MIR3615 |  | NUBPL |  | MPO |
|  | HIF1A |  | ATP5MF |  | RBM39 |  | CHI3L1 |
|  | CDKN1A |  | MTCH2 |  | LMTK3 |  | HLA-DRB1 |
|  | FAS |  | PAPSS2 |  | PRG2 |  | CLDN3 |
|  | IL6 |  | RBM28 |  | KPNB1 |  | CDCP1 |
|  | IL2 |  | SLC26A3 |  | BCAM |  | NOTCH2 |
|  | MIRLET7C |  | PI4KB |  | FBXW11 |  | TNFRSF6B |
|  | MMP9 |  | RNASE3 |  | USP9X |  | CSE1L |
|  | TNF |  | PSMA6 |  | CRHR1 |  | HDAC3 |
|  | PTPN11 |  | GJA9-MYCBP |  | GOT1 |  | CSNK2A2 |
|  | MMP2 |  | WDTC1 |  | USP10 |  | CDC25A |
|  | KDR |  | PGAP6 |  | SIRT5 |  | PIK3R3 |
|  | ERBB4 |  | PSD |  | NAV2 |  | LRP5 |
|  | IL1B |  | MIR938 |  | DAPK3 |  | DCN |
|  | MIR149 |  | RIMS1 |  | PITX1-AS1 |  | TLR5 |
|  | RECQL4 |  | IRAK3 |  | FMR1 |  | IDO1 |
|  | MUC2 |  | MLXIP |  | CLSPN |  | HLA-DQB1 |
|  | BIRC5 |  | RGS10 |  | SNTB2 |  | TAGLN |
|  | CDK2 |  | ZBTB18 |  | IRF5 |  | NDRG2 |
|  | CD274 |  | PANK2 |  | PFKFB3 |  | TRIM28 |
|  | MGMT |  | CCNY |  | DDX18 |  | HIPK2 |
|  | CASP3 |  | NFKBIZ |  | MFF |  | EWSR1 |
|  | VDR |  | BVES |  | STAU1 |  | IGF2BP1 |
|  | MUC6 |  | RCL1 |  | HSD17B10 |  | SELENOP |
|  | NOTCH1 |  | SIGIRR |  | SCRN1 |  | VCL |
|  | CXCL8 |  | YBX2 |  | GSDMB |  | ACVR2A |
|  | MUC1 |  | TNPO3 |  | NOP58 |  | HSPG2 |
|  | MIR26B |  | STARD7 |  | TUFM |  | RACK1 |
|  | ABL1 |  | PARP12 |  | FFAR4 |  | NR1H2 |
|  | YAP1 |  | REEP1 |  | CBX1 |  | CASP5 |
|  | SMAD3 |  | HECTD1 |  | AP2A1 |  | ACLY |
|  | CD44 |  | ARMC10 |  | SERPINC1 |  | IL22 |
|  | NFKB1 |  | RBM47 |  | GPR35 |  | TRAF6 |
|  | MIR98 |  | SLC9A8 |  | RBBP5 |  | PRMT1 |
|  | CXCR4 |  | KDM4D |  | HAMP |  | PYCARD |
|  | TCF7L2 |  | ZNF280C |  | CRH |  | MAD2L1 |
|  | MIR193A |  | FHIP2B |  | MYCBP2 |  | PGR-AS1 |
|  | SMAD2 |  | INPP5E |  | MUC13 |  | DDX5 |
|  | BCL2L1 |  | APEH |  | WDR54 |  | NFKB2 |
|  | MIR16-1 |  | GPR12 |  | CDC23 |  | NR1H4 |
|  | IFNG |  | EXOC3-AS1 |  | ACTR2 |  | EPHB3 |
|  | RAC1 |  | MIR3150A |  | PAICS |  | CLDN18 |
|  | CDX2 |  | EXOC3 |  | PPP2CB |  | FCGR2A |
|  | TLR4 |  | RNF186 |  | RGN |  | COPS5 |
|  | IL10 |  | INAVA |  | ST7 |  | EDNRA |
|  | IL1RN |  | FFAR2 |  | CDCA8 |  | SPINT1 |
|  | JAK2 |  | CLEC4D |  | PTPN2 |  | EEF1A1 |
|  | MIR193B |  | BSN |  | KNTC1 |  | RBX1 |
|  | MAPK3 |  | HLA-DRB9 |  | DARS1 |  | TFPI2 |
|  | TCF7 |  | COG6 |  | HADHA |  | FOXP2 |
|  | IDH2 |  | IAH1 |  | CLK2 |  | RNU6-1 |
|  | KRT20 |  | PLA2G2A |  | FPR1 |  | KMT2A |
|  | STAT1 |  | EPHB2 |  | NRBP1 |  | F2RL1 |
|  | E2F1 |  | NRAS |  | PSME3 |  | SKP1 |
|  | MIR7-3HG |  | FASLG |  | TARS1 |  | KHDRBS1 |
|  | TNFSF10 |  | BLACAT1 |  | MMS19 |  | MIR1-2 |
|  | CASP9 |  | FH |  | PSMA2 |  | FOLR1 |
|  | MIR181B1 |  | BUB1 |  | TMPRSS6 |  | EIF4A1 |
|  | SNAI1 |  | CASP10 |  | NEIL2 |  | MCM3 |
|  | PHB1 |  | CASP8 |  | DPM1 |  | HLA-DQA1 |
|  | MIR429 |  | BARD1 |  | ILF2 |  | SATB2 |
|  | ALDH2 |  | ATR |  | UBAP2 |  | MIR506 |
|  | RUNX3 |  | PIK3CA |  | FKBP4 |  | FBXO11 |
|  | MUC5AC |  | ADH1B |  | ASAH1 |  | MDK |
|  | LINC01672 |  | MCC |  | SIAH2 |  | WNT4 |
|  | SETD2 |  | IRF1 |  | COPA |  | PDGFA |
|  | KRT7 |  | HMMR |  | CAPZA2 |  | IL1R1 |
|  | MAPK8 |  | FGFR4 |  | CRTC1 |  | NTSR1 |
|  | PROM1 |  | SASH1 |  | NFATC3 |  | HCCAT5 |
|  | RMRP |  | ESR1 |  | ELK4 |  | MMP10 |
|  | TGFA |  | PRKN |  | PIM3 |  | CUL4A |
|  | PLK1 |  | CAHM |  | EIF5B |  | TNFRSF9 |
|  | FGF2 |  | PMS2 |  | IARS1 |  | SGPL1 |
|  | MIR124-3 |  | PTPN12 |  | TNFSF8 |  | DEK |
|  | MIR301A |  | DLC1 |  | DDX39A |  | CX3CR1 |
|  | PKM |  | PDGFRL |  | ADSL |  | ANG |
|  | NOS2 |  | RAD54B |  | FKBP8 |  | BRD4 |
|  | RELA |  | CRCS6 |  | STING1 |  | SPINT2 |
|  | TOP1 |  | CRCS2 |  | NAP1L1 |  | RTEL1 |
|  | PAK1 |  | PCAT1 |  | MARCKS |  | TXNIP |
|  | CERNA3 |  | PCAT2 |  | CCT5 |  | EMSY |
|  | VIM |  | PRNCR1 |  | FDFT1 |  | KIF15 |
|  | CCNA2 |  | CASC19 |  | TNFAIP8 |  | TRPM8 |
|  | CTSD |  | CASC8 |  | UBAP2L |  | FKBP5 |
|  | NLRP3 |  | CCAT2 |  | UBE3C |  | TRAF2 |
|  | HDAC1 |  | CASC11 |  | TECTA |  | MAP3K5 |
|  | AREG |  | GALNT12 |  | GNL2 |  | S100B |
|  | LGALS3 |  | KLF6 |  | RIPK3 |  | CXCL9 |
|  | SPINK1 |  | CRCS5 |  | GOLGB1 |  | VDAC1 |
|  | IL7R |  | MAP3K8 |  | RPN2 |  | MIR543 |
|  | FOXM1 |  | ERCC6 |  | TNFRSF14 |  | HSPA6 |
|  | XPO1 |  | MXI1 |  | GPSM3 |  | EIF4G1 |
|  | FASN |  | CASC2 |  | ANGPTL2 |  | RPL6 |
|  | HMGB1 |  | HRAS |  | PNMT |  | DDX3X |
|  | RXRA |  | PTPRJ |  | HLA-DRB5 |  | CTC1 |
|  | IL4 |  | CTTN |  | PUS10 |  | PRMT5 |
|  | PRKDC |  | ATM |  | POLA2 |  | PRDX6 |
|  | MIR4435-2HG |  | CRCS7 |  | GIGYF2 |  | MST1 |
|  | BMI1 |  | PPP2R1B |  | EIF3F |  | F5 |
|  | FOS |  | POLE |  | MICAL1 |  | TNFAIP3 |
|  | IL17A |  | BRCA2 |  | MIR4689 |  | MED15 |
|  | CCR6 |  | RB1 |  | SEC13 |  | GAB1 |
|  | CFLAR |  | NKX2-1 |  | STK3 |  | CHD4 |
|  | CTSB |  | CRCS8 |  | GNA12 |  | PKD1 |
|  | DAPK1 |  | MLH3 |  | SETD1A |  | ID2 |
|  | MMP3 |  | XRCC3 |  | EIF3D |  | GNL3 |
|  | CDC42 |  | AKT1 |  | CRHR2 |  | MAGED2 |
|  | GAPDH |  | BUB1B |  | SSBP1 |  | FUT8 |
|  | ANXA5 |  | RAD51 |  | CIRBP |  | WNT16 |
|  | TCF4 |  | CHRNA5 |  | ZFP64 |  | LATS1 |
|  | TMX2-CTNND1 |  | IQGAP1 |  | SLC2A4RG |  | CLDN2 |
|  | IL6R |  | BCAR4 |  | USP25 |  | ADM |
|  | NDRG1 |  | CRNDE |  | DOCK7 |  | PIAS3 |
|  | MIR381 |  | CRCS9 |  | CNR2 |  | XDH |
|  | CDH3 |  | NQO1 |  | POLA1 |  | DVL2 |
|  | SERPINB5 |  | FLCN |  | IL17RB |  | BCL3 |
|  | LGALS1 |  | RAD51D |  | CSN1S1 |  | KRR1 |
|  | MTA1 |  | ERBB2 |  | SMOX |  | DAB2 |
|  | TLR9 |  | BRCA1 |  | SLC11A1 |  | HIC2 |
|  | RIPK1 |  | RNF43 |  | GATD3 |  | CTBP2 |
|  | HERC2 |  | PPM1D |  | RIGI |  | LSP1 |
|  | CXCL1 |  | BRIP1 |  | CUEDC2 |  | GPRC5A |
|  | ALDH1A1 |  | AXIN2 |  | RANBP1 |  | STIM1 |
|  | CSF3 |  | RHBDF2 |  | TRAPPC4 |  | BCAS2 |
|  | HBEGF |  | STK11 |  | VPS16 |  | TNFSF12 |
|  | SOCS1 |  | UCA1 |  | MIR4284 |  | DDB1 |
|  | MMP13 |  | CYP2A6 |  | WDR48 |  | IL12B |
|  | FOXD2-AS1 |  | CRCS11 |  | VPS35 |  | DYRK1B |
|  | DDB2 |  | SRC |  | NUP210 |  | SERPINH1 |
|  | NTRK3 |  | NCOA3 |  | GGA1 |  | ATP1A1 |
|  | HPSE |  | AURKA |  | DCAF4 |  | SLC7A5 |
|  | HDAC2 |  | TFF1 |  | LAP3 |  | NRIP1 |
|  | FSCN1 |  | CHEK2 |  | MARS1 |  | PRSS8 |
|  | PTGER4 |  | AR |  | CASP4 |  | OLFM4 |
|  | ILK |  | CAGE1 |  | GOLIM4 |  |  |
|  | AXL |  | TTF2 |  | USP34 |  |  |

**Table 6｜Core Targets of PPI network**

| No. | Target | No. | Target | No. | Target | No. | Target |
| --- | --- | --- | --- | --- | --- | --- | --- |
|  | MMP3 |  | MTOR |  | HSP90B1 |  | CX3CR1 |
|  | IDO1 |  | ABL1 |  | ERBB4 |  | NFKB1 |
|  | TACR1 |  | PRKDC |  | TRAP1 |  | RELA |
|  | EPHB2 |  | SRC |  | ATR |  | TMPRSS6 |
|  | EPHB3 |  | CDK2 |  | S100B |  | HPSE |
|  | EDNRA |  | CDK4 |  | PNMT |  | SIRT5 |
|  | TOP1 |  | PLK1 |  | KMT2A |  | IL1B |
|  | PPARG |  | AURKA |  | PRMT1 |  | MMP10 |
|  | PTPN2 |  | MMP9 |  | CXCR4 |  | LGALS3 |
|  | NTSR1 |  | MMP2 |  | ILK |  | ABL2 |
|  | PTGS2 |  | BRD4 |  | ALDH1A1 |  | TGFBR2 |
|  | PIK3CA |  | CTSD |  | NR1H2 |  | STK3 |
|  | CTSB |  | MPO |  | POLA1 |  | RIPK3 |
|  | MAP3K8 |  | CXCR2 |  | PI4KB |  | FASN |
|  | CXCL8 |  | AKT1 |  | CLK2 |  | ATP1A1 |
|  | CTNNB1 |  | TRPM8 |  | KDM4D |  | FGF2 |
|  | PTPN11 |  | BRAF |  | GPR35 |  | FOLR1 |
|  | CDC25A |  | MMP13 |  | FGFR3 |  | SLC19A1 |
|  | NOS2 |  | CNR2 |  | SLC7A5 |  | FGFR2 |
|  | MAPK3 |  | MAP3K5 |  | TLR4 |  | F5 |
|  | TERT |  | CASP1 |  | LIMK2 |  | HRAS |
|  | AR |  | CRHR1 |  | NFE2L2 |  | FFAR4 |
|  | TNF |  | CASP8 |  | EP300 |  | HNF4A |
|  | ESR1 |  | GSK3A |  | RXRA |  | RIPK1 |
|  | MIF |  | HDAC3 |  | SPHK1 |  | FPR1 |
|  | PTGER4 |  | NR1H4 |  | RAD51 |  | CDC42 |
|  | IL6 |  | VDR |  | RHOA |  | NDUFA4 |
|  | ADAM17 |  | FBP1 |  | HIF1A |  | PRMT5 |
|  | JAK2\| |  | ERBB2 |  | VEGFA |  | BRCA1 |
|  | IL2 |  | CASP4 |  | GAPDH |  | DAPK1 |
|  | ALDH2 |  | CASP9 |  | ATM |  | PIK3R1 |
|  | EGFR |  | MAPK8 |  | CXCR1 |  | CAMK2G |
|  | XDH |  | PRTN3 |  | CSNK2A2 |  | FKBP5 |
|  | PFKFB3 |  | PIM3 |  | ACLY |  | STAT6 |
|  | ALOX15 |  | NOD2 |  | BIRC2 |  | TP53 |
|  | TLR9 |  | DYRK1B |  | HLA-DRB1 |  | STAT1 |
|  | JUN |  | DAPK3 |  | ITGA4 |  | RAC1 |
|  | MDM2 |  | ADH1B |  | CTBP2 |  | SETD2 |
|  | F2RL1 |  | FDFT1 |  | AXL |  | PAK1 |
|  | MAPK9 |  | HDAC1 |  | CHEK2 |  | DDOST |
|  | NLRP3 |  | ASAH1 |  | NQO1 |  | MGMT |
|  | BCL2L1 |  | IDH1 |  | CSNK1A1 |  | CCR6 |
|  | CYP2A6 |  | PLA2G2A |  | SGPL1 |  | GALK1 |
|  | KDR |  | LAP3 |  | PKM |  | BIRC5 |
|  | WEE1 |  | AHR |  | USP10 |  | CCNA2 |
|  | EIF4A1 |  | BCL2 |  | CTSG |  | DDX3X |
|  | HDAC2 |  | STAT3 |  | XPO1 |  | CD274 |
|  | CASP3 |  |  |  |  |  |  |

**Table 7｜Top 100 results of KEGG enrichment**

| No. | Term | Count | PValue | Fold Enrichment | FDR |
| --- | --- | --- | --- | --- | --- |
|  | hsa05200:Pathways in cancer | 66 | 1.90E-35 | 6.20457233 | 2.73E-33 |
|  | hsa05417:Lipid and atherosclerosis | 37 | 7.54E-24 | 8.590643591 | 5.43E-22 |
|  | hsa05205:Proteoglycans in cancer | 32 | 3.11E-19 | 7.792172433 | 8.95E-18 |
|  | hsa05165:Human papillomavirus infection | 32 | 3.15E-13 | 4.825967821 | 1.62E-12 |
|  | hsa05161:Hepatitis B | 31 | 3.03E-21 | 9.552325581 | 1.45E-19 |
|  | hsa05132:Salmonella infection | 31 | 8.95E-16 | 6.214766041 | 9.21E-15 |
|  | hsa04151:PI3K-Akt signaling pathway | 31 | 1.51E-11 | 4.310520179 | 5.43E-11 |
|  | hsa05163:Human cytomegalovirus infection | 30 | 4.08E-16 | 6.655813953 | 5.87E-15 |
|  | hsa05131:Shigellosis | 30 | 5.65E-15 | 6.062988419 | 4.28E-14 |
|  | hsa05167:Kaposi sarcoma-associated herpesvirus infection | 28 | 7.74E-16 | 7.204747063 | 8.57E-15 |
|  | hsa04010:MAPK signaling pathway | 28 | 3.52E-11 | 4.64359113 | 1.10E-10 |
|  | hsa05152:Tuberculosis | 26 | 1.02E-14 | 7.210465116 | 7.33E-14 |
|  | hsa05130:Pathogenic Escherichia coli infection | 26 | 9.54E-14 | 6.554968288 | 5.97E-13 |
|  | hsa05169:Epstein-Barr virus infection | 26 | 1.53E-13 | 6.425166935 | 9.17E-13 |
|  | hsa05170:Human immunodeficiency virus 1 infection | 26 | 4.66E-13 | 6.122093023 | 2.31E-12 |
|  | hsa05166:Human T-cell leukemia virus 1 infection | 26 | 1.34E-12 | 5.846323067 | 5.66E-12 |
|  | hsa05010:Alzheimer disease | 26 | 1.41E-07 | 3.379905523 | 2.66E-07 |
|  | hsa05022:Pathways of neurodegeneration - multiple diseases | 26 | 7.34E-06 | 2.726646473 | 1.08E-05 |
|  | hsa05203:Viral carcinogenesis | 25 | 1.50E-12 | 6.11747606 | 6.19E-12 |
|  | hsa05206:MicroRNAs in cancer | 25 | 9.42E-09 | 4.025693923 | 2.05E-08 |
|  | hsa04933:AGE-RAGE signaling pathway in diabetic complications | 24 | 1.08E-18 | 11.98046512 | 2.58E-17 |
|  | hsa04625:C-type lectin receptor signaling pathway | 24 | 2.77E-18 | 11.519678 | 5.70E-17 |
|  | hsa04210:Apoptosis | 24 | 1.57E-15 | 8.809165527 | 1.50E-14 |
|  | hsa05162:Measles | 24 | 2.25E-15 | 8.681496461 | 2.02E-14 |
|  | hsa05418:Fluid shear stress and atherosclerosis | 24 | 2.61E-15 | 8.619039652 | 2.21E-14 |
|  | hsa04621:NOD-like receptor signaling pathway | 24 | 1.59E-12 | 6.441110278 | 6.19E-12 |
|  | hsa05208:Chemical carcinogenesis - reactive oxygen species | 24 | 7.04E-11 | 5.372405882 | 2.02E-10 |
|  | hsa04014:Ras signaling pathway | 24 | 2.22E-10 | 5.07646827 | 5.62E-10 |
|  | hsa05212:Pancreatic cancer | 23 | 2.69E-20 | 15.10694614 | 9.68E-19 |
|  | hsa04064:NF-kappa B signaling pathway | 13 | 9.34E-07 | 6.239825581 | 1.53E-06 |
|  | hsa05215:Prostate cancer | 23 | 8.81E-18 | 11.83637018 | 1.59E-16 |
|  | hsa04668:TNF signaling pathway | 23 | 3.44E-16 | 10.07129743 | 5.50E-15 |
|  | hsa04510:Focal adhesion | 23 | 7.15E-11 | 5.655802497 | 2.02E-10 |
|  | hsa05207:Chemical carcinogenesis - receptor activation | 23 | 1.68E-10 | 5.415697674 | 4.49E-10 |
|  | hsa05135:Yersinia infection | 22 | 2.22E-13 | 8.016126294 | 1.28E-12 |
|  | hsa04062:Chemokine signaling pathway | 22 | 1.68E-10 | 5.719840116 | 4.49E-10 |
|  | hsa04932:Non-alcoholic fatty liver disease | 21 | 2.28E-11 | 6.763165791 | 7.65E-11 |
|  | hsa05160:Hepatitis C | 21 | 3.27E-11 | 6.634751251 | 1.05E-10 |
|  | hsa05164:Influenza A | 21 | 1.40E-10 | 6.130354957 | 3.89E-10 |
|  | hsa05171:Coronavirus disease - COVID-19 | 21 | 3.01E-08 | 4.518494387 | 6.20E-08 |
|  | hsa05168:Herpes simplex virus 1 infection | 21 | 0.002867528 | 2.047442769 | 0.003176338 |
|  | hsa01521:EGFR tyrosine kinase inhibitor resistance | 20 | 5.26E-16 | 12.63762143 | 6.89E-15 |
|  | hsa05210:Colorectal cancer | 20 | 3.09E-15 | 11.60897783 | 2.47E-14 |
|  | hsa01522:Endocrine resistance | 20 | 3.94E-14 | 10.18747034 | 2.70E-13 |
|  | hsa04620:Toll-like receptor signaling pathway | 20 | 2.53E-13 | 9.244186047 | 1.40E-12 |
|  | hsa04066:HIF-1 signaling pathway | 20 | 3.01E-13 | 9.159377 | 1.61E-12 |
|  | hsa04722:Neurotrophin signaling pathway | 20 | 1.56E-12 | 8.389681454 | 6.19E-12 |
|  | hsa04919:Thyroid hormone signaling pathway | 20 | 2.12E-12 | 8.251009033 | 8.05E-12 |
|  | hsa04218:Cellular senescence | 20 | 2.07E-10 | 6.399821109 | 5.31E-10 |
|  | hsa05142:Chagas disease | 19 | 1.05E-12 | 9.298563611 | 4.75E-12 |
|  | hsa04071:Sphingolipid signaling pathway | 19 | 2.14E-11 | 7.838458582 | 7.35E-11 |
|  | hsa05226:Gastric cancer | 19 | 7.29E-10 | 6.365459654 | 1.69E-09 |
|  | hsa05202:Transcriptional misregulation in cancer | 19 | 4.63E-08 | 4.914266779 | 9.27E-08 |
|  | hsa05220:Chronic myeloid leukemia | 18 | 7.57E-14 | 11.82282742 | 4.96E-13 |
|  | hsa04012:ErbB signaling pathway | 18 | 5.42E-13 | 10.57099863 | 2.60E-12 |
|  | hsa05235:PD-L1 expression and PD-1 checkpoint pathway in cancer | 18 | 1.20E-12 | 10.09589757 | 5.24E-12 |
|  | hsa04659:Th17 cell differentiation | 18 | 3.15E-11 | 8.319767442 | 1.03E-10 |
|  | hsa05145:Toxoplasmosis | 18 | 4.95E-11 | 8.094908862 | 1.48E-10 |
|  | hsa04660:T cell receptor signaling pathway | 18 | 2.02E-10 | 7.42590813 | 5.29E-10 |
|  | hsa04926:Relaxin signaling pathway | 18 | 5.64E-10 | 6.965386696 | 1.38E-09 |
|  | hsa04068:FoxO signaling pathway | 18 | 7.20E-10 | 6.859044914 | 1.69E-09 |
|  | hsa04936:Alcoholic liver disease | 18 | 2.56E-09 | 6.327710449 | 5.77E-09 |
|  | hsa04613:Neutrophil extracellular trap formation | 18 | 2.21E-07 | 4.704371119 | 3.98E-07 |
|  | hsa04015:Rap1 signaling pathway | 18 | 8.55E-07 | 4.278737542 | 1.42E-06 |
|  | hsa04024:cAMP signaling pathway | 18 | 2.23E-06 | 3.993488372 | 3.49E-06 |
|  | hsa04810:Regulation of actin cytoskeleton | 18 | 2.84E-06 | 3.923733117 | 4.40E-06 |
|  | hsa01524:Platinum drug resistance | 17 | 5.96E-13 | 11.62488054 | 2.77E-12 |
|  | hsa04657:IL-17 signaling pathway | 17 | 3.61E-11 | 9.027832756 | 1.11E-10 |
|  | hsa05224:Breast cancer | 17 | 3.08E-08 | 5.772899858 | 6.24E-08 |
|  | hsa04217:Necroptosis | 17 | 9.38E-08 | 5.337209302 | 1.83E-07 |
|  | hsa05225:Hepatocellular carcinoma | 17 | 2.02E-07 | 5.051287375 | 3.69E-07 |
|  | hsa04360:Axon guidance | 17 | 6.06E-07 | 4.662726808 | 1.01E-06 |
|  | hsa05219:Bladder cancer | 16 | 6.51E-16 | 19.48043108 | 7.81E-15 |
|  | hsa05120:Epithelial cell signaling in Helicobacter pylori infection | 16 | 4.63E-12 | 11.40996678 | 1.71E-11 |
|  | hsa05133:Pertussis | 16 | 1.64E-11 | 10.50917993 | 5.77E-11 |
|  | hsa05222:Small cell lung cancer | 16 | 2.88E-10 | 8.681496461 | 7.14E-10 |
|  | hsa04110:Cell cycle | 16 | 4.75E-07 | 5.087246334 | 8.15E-07 |
|  | hsa04630:JAK-STAT signaling pathway | 16 | 9.76E-07 | 4.811431774 | 1.58E-06 |
|  | hsa05415:Diabetic cardiomyopathy | 16 | 1.19E-05 | 3.934471303 | 1.69E-05 |
|  | hsa05230:Central carbon metabolism in cancer | 15 | 6.64E-11 | 10.69684385 | 1.95E-10 |
|  | hsa04380:Osteoclast differentiation | 15 | 4.26E-07 | 5.546511628 | 7.40E-07 |
|  | hsa04915:Estrogen signaling pathway | 15 | 5.12E-07 | 5.465540655 | 8.67E-07 |
|  | hsa04140:Autophagy - animal | 15 | 4.80E-06 | 4.538054968 | 7.28E-06 |
|  | hsa04020:Calcium signaling pathway | 15 | 5.10E-04 | 2.959601066 | 6.17E-04 |
|  | hsa05211:Renal cell carcinoma | 14 | 7.21E-10 | 10.12841254 | 1.69E-09 |
|  | hsa04370:VEGF signaling pathway | 13 | 1.31E-09 | 10.99901458 | 3.01E-09 |
|  | hsa05321:Inflammatory bowel disease | 13 | 4.27E-09 | 9.98372093 | 9.45E-09 |
|  | hsa04917:Prolactin signaling pathway | 13 | 1.03E-08 | 9.270598007 | 2.22E-08 |
|  | hsa05223:Non-small cell lung cancer | 13 | 1.44E-08 | 9.013081395 | 3.01E-08 |
|  | hsa04931:Insulin resistance | 13 | 1.41E-06 | 6.00872093 | 2.22E-06 |
|  | hsa04072:Phospholipase D signaling pathway | 13 | 3.69E-05 | 4.3847423 | 5.01E-05 |
|  | hsa04144:Endocytosis | 13 | 0.004173836 | 2.595767442 | 0.004485317 |
|  | hsa05020:Prion disease | 13 | 0.008058778 | 2.385815663 | 0.00847054 |
|  | hsa05016:Huntington disease | 13 | 0.019072277 | 2.120725034 | 0.019617199 |
|  | hsa05014:Amyotrophic lateral sclerosis | 13 | 0.05935151 | 1.782807309 | 0.05935151 |
|  | hsa05213:Endometrial cancer | 12 | 1.43E-08 | 10.32798717 | 3.01E-08 |
|  | hsa05221:Acute myeloid leukemia | 12 | 6.93E-08 | 8.940645609 | 1.37E-07 |
|  | hsa04115:p53 signaling pathway | 12 | 2.00E-07 | 8.094908862 | 3.69E-07 |
|  | hsa05214:Glioma | 12 | 2.30E-07 | 7.986976744 | 4.09E-07 |
|  | hsa05140:Leishmaniasis | 12 | 3.03E-07 | 7.779522803 | 5.33E-07 |

**Table 8｜Top 30 results of GO enrichment**

| No. | Term | Enrichment | P Value | Count | CLASS |
| --- | --- | --- | --- | --- | --- |
|  | negative regulation of apoptotic process | 23.93617021 | 2.19E-28 | 45 | BP |
|  | protein phosphorylation | 21.27659574 | 2.27E-26 | 40 | BP |
|  | inflammatory response | 19.14893617 | 2.03E-22 | 36 | BP |
|  | cellular response to lipopolysaccharide | 13.82978723 | 5.64E-21 | 26 | BP |
|  | positive regulation of transcription from RNA polymerase II promoter | 27.12765957 | 7.63E-19 | 51 | BP |
|  | positive regulation of cell proliferation | 18.61702128 | 2.33E-18 | 35 | BP |
|  | protein autophosphorylation | 12.23404255 | 3.69E-18 | 23 | BP |
|  | positive regulation of gene expression | 18.08510638 | 4.41E-18 | 34 | BP |
|  | positive regulation of transcription, DNA-templated | 20.74468085 | 8.09E-18 | 39 | BP |
|  | positive regulation of apoptotic process | 14.89361702 | 2.02E-17 | 28 | BP |
|  | cytosol | 60.63829787 | 1.13E-21 | 114 | CC |
|  | macromolecular complex | 20.21276596 | 2.33E-18 | 38 | CC |
|  | cytoplasm | 57.9787234 | 1.03E-17 | 109 | CC |
|  | nucleoplasm | 46.80851064 | 6.83E-17 | 88 | CC |
|  | nucleus | 56.38297872 | 2.68E-14 | 106 | CC |
|  | receptor complex | 8.510638298 | 2.22E-09 | 16 | CC |
|  | membrane raft | 8.510638298 | 8.77E-09 | 16 | CC |
|  | plasma membrane | 45.74468085 | 1.46E-08 | 86 | CC |
|  | chromatin | 15.95744681 | 2.75E-07 | 30 | CC |
|  | cell surface | 11.70212766 | 5.92E-07 | 22 | CC |
|  | protein serine/threonine/tyrosine kinase activity | 23.40425532 | 2.04E-30 | 44 | MF |
|  | protein kinase activity | 19.14893617 | 6.70E-24 | 36 | MF |
|  | ATP binding | 32.44680851 | 2.27E-21 | 61 | MF |
|  | protein serine/threonine kinase activity | 17.55319149 | 4.45E-20 | 33 | MF |
|  | enzyme binding | 17.0212766 | 1.51E-19 | 32 | MF |
|  | identical protein binding | 32.9787234 | 1.65E-19 | 62 | MF |
|  | protein binding | 93.08510638 | 4.16E-18 | 175 | MF |
|  | protein tyrosine kinase activity | 8.510638298 | 4.06E-13 | 16 | MF |
|  | protein kinase binding | 13.82978723 | 6.85E-11 | 26 | MF |
|  | RNA polymerase II transcription factor activity, ligand-activated sequence-specific DNA binding | 5.85106383 | 1.12E-10 | 11 | MF |
